# Small but Powerful, the Primary Endosymbiont of Moss Bugs, *Candidatus* Evansia muelleri, Holds a Reduced Genome with Large Biosynthetic Capabilities

**DOI:** 10.1093/gbe/evu149

**Published:** 2014-07-10

**Authors:** Diego Santos-Garcia, Amparo Latorre, Andrés Moya, George Gibbs, Viktor Hartung, Konrad Dettner, Stefan Martin Kuechler, Francisco J. Silva

**Affiliations:** ^1^Institut Cavanilles de Biodiversitat i Biologia Evolutiva, Universitat de València, Spain; ^2^Unidad Mixta de Investigación en Genómica y Salud (FISABIO-Salud Pública and Universitat de València), Spain; ^3^School of Biological Science, Victoria University, Wellington, New Zealand; ^4^Museum für Naturkunde, Leibniz-Institute for Research on Evolution and Biodiversity, Berlin, Germany; ^5^Department of Animal Ecology II, University of Bayreuth, Germany

**Keywords:** genome reduction, endosymbiont, Halomonadaceae, mutualism, metabolic complementation

## Abstract

Moss bugs (Coleorrhyncha: Peloridiidae) are members of the order Hemiptera, and like many hemipterans, they have symbiotic associations with intracellular bacteria to fulfill nutritional requirements resulting from their unbalanced diet. The primary endosymbiont of the moss bugs*, Candidatus* Evansia muelleri, is phylogenetically related to *Candidatus* Carsonella ruddii and *Candidatus* Portiera aleyrodidarum, primary endosymbionts of psyllids and whiteflies, respectively. In this work, we report the genome of *Candidatus* Evansia muelleri Xc1 from *Xenophyes cascus,* which is the only obligate endosymbiont present in the association. This endosymbiont possesses an extremely reduced genome similar to *Carsonella* and *Portiera*. It has crossed the borderline to be considered as an autonomous cell, requiring the support of the insect host for some housekeeping cell functions. Interestingly, in spite of its small genome size, *Evansia* maintains enriched amino acid (complete or partial pathways for ten essential and six nonessential amino acids) and sulfur metabolisms, probably related to the poor diet of the insect, based on bryophytes, which contains very low levels of nitrogenous and sulfur compounds. Several facts, including the congruence of host (moss bugs, whiteflies, and psyllids) and endosymbiont phylogenies and the retention of the same ribosomal RNA operon during genome reduction in *Evansia*, *Portiera,* and *Carsonella*, suggest the existence of an ancient endosymbiotic Halomonadaceae clade associated with Hemiptera. Three possible scenarios for the origin of these three primary endosymbiont genera are proposed and discussed.

## Introduction

Most members of the hemipteran suborders, Sternorrhyncha (psyllids, whiteflies, aphids, and coccoids), Auchenorrhyncha (Cicadomorpha: Cicadas, spittlebugs, leafhoppers, and treehoppers; Fulgoromorpha: Planthoppers), Heteroptera (lygaeoid, coreoid, and pentatomoid bugs), and Coleorrhyncha (moss bugs), are known to be associated with one or more obligate primary endosymbiont bacteria (Buchner 1965; Baumann 2005). They include representatives from several classes such as Gammaproteobacteria, Betaproteobacteria, Alphaproteobacteria, or Bacteroidetes, with the former being to date the most frequently reported (Moya et al*.* 2008).

All these hemipteran hosts are nutrient specialists and feed exclusively on restricted diets like xylem/phloem plant sap (Auchenorrhyncha and Sternorrhyncha) or seeds (Heteroptera), which are rich in carbohydrates, mostly sugars, but are deficient in most essential amino acids and many cofactors, like vitamins (Hansen and Moran 2014). Consequently, the lack of essential elements is compensated by the metabolic abilities of the symbionts. The frequent coexistence of obligate primary endosymbionts (P-endosymbionts) with facultative secondary ones may lead to the replacement of the ancestral P-endosymbiont or to the emergence of a coprimary obligate endosymbiosis, with both partners sharing the biosynthesis of the essential compounds required for nutrient provisioning (McCutcheon et al. 2009; Lamelas et al. 2011).

All P-endosymbionts display a large genome reduction, because of their accommodation to an intracellular lifestyle (Moya et al*.* 2008; McCutcheon and Moran 2012). As a consequence of their small population size and strict vertical transmission (bottleneck effect), they accumulate deleterious mutations, resulting in the loss of gene functions (Moran 1996; Allen et al. 2009), and finally in a complete gene disintegration (Silva et al*.* 2001). Particularly, functions related to DNA replication, transcription, and translation are subjected to an extreme decline, which in some cases imply that the genome is no longer compatible with its role as a mutualistic endosymbiont and that the endosymbiont has crossed the line between a cell and an organelle (Tamames et al*.* 2007). Because of the fact that some important differences among extreme symbionts and organelles still remain, the term symbionelle has been proposed recently to name the former (Reyes-Prieto et al*.* 2013). P-endosymbionts having crossed this critical threshold may include *Candidatus* Tremblaya princeps in mealybugs (139–171 kb), *Candidatus* Hodgkinia cicadicola in cicadas (144 kb), *Candidatus* Carsonella ruddii (158–166 kb) (hereafter referred to as *Carsonella*) in psyllids or *Candidatus* Portiera aleyrodidarum (281–358 kb) (hereafter referred to as *Portiera*) in whiteflies.

As described for the exceptional number of gene losses in *Carsonella* (Sloan et al. 2014), the loss of essential genes in endosymbionts with extremely reduced genome sizes may be compensated by at least three mechanisms: 1) modification of highly conserved cellular processes or selection of multifunctional proteins (Kelkar and Ochman 2013), 2) complementation by a second endosymbiont (Hansen and Moran 2014), and 3) substitution of the lost functions by host proteins of either eukaryotic or bacterial origins (Husnik et al. 2013).

The enigmatic suborder Coleorrhyncha is represented by only one extant family, called Peloridiidae. The peloridiids or moss bugs, consisting of 17 genera with 36 species, are very small, strange-looking insects (body length 2–5 mm) with a very cryptic life style, resulting in a limited representation in collections and even less information about their biology. However, recent studies have offered some new data in respect of vibrational signaling (Hoch et al. 2006), jumping behavior (Burrows et al. 2007), or cephalic morphology (Spangenberg et al*.* 2013).

Coleorrhyncha existed 250 Ma, since the late Permian and Cretaceous period, as demonstrated by numerous fossils mostly from the Northern hemisphere (Popov and Shcherbakov 1996). However, the present distribution of Peloridiidae is restricted to the temperate and subantartic rainforests (along with *Nothofagus*) or sphagnum bogs of the Southern hemisphere (Argentina, Chile, New Zealand, New Caledonia, and eastern Australia from North Queensland to Tasmania), reflecting a typical Gondwana biogeography (Burckhardt 2009). Peloridiids live in wet moss or leaf litter and feed on sap of different mosses and liverworts, preferring bryophyte species that provide a stable wet environment and possess conductive tissue (Hartung V, unpublished data). This kind of nutrition is quite special, in that most animals, especially insects, avoid feeding on mosses because of their poor nutrient content, on the one hand, and the high amount of toxic secondary metabolites, on the other hand (Klavina et al*.* 2012; Asakawa et al*.* 2013).

On the basis of this restricted kind of nutrition, moss bugs possess, like many other plant-sap sucking Hemiptera, endosymbiotic microorganisms, which are located in specific organ-like structures called bacteriomes. This observation was first recorded by Evans (1948) in a *Hemiodoecus fidelis* larva and a more detailed morphological overview was later given by Müller (1951) and Pendergrast (1962). In almost all host species examined, the endosymbionts are harbored inside a type of evolutionarily adapted insect cells called bacteriocytes, located in a pair of slightly orange-colored bacteriomes, subdivided into three partial bacteriomes of spherical shape on each side of the abdomen. Molecular characterization revealed that the P-endosymbionts of the Peloridiidae, called *Candidatus* Evansia muelleri (Oceanospirillales: Halomonadaceae), are closely related to *Carsonella* and *Portiera*, P-endosymbionts of psyllids (Hemiptera: Sternorrhyncha: Psyllidae) and whiteflies (Hemiptera: Sternorrhyncha: Aleyrodidae), respectively (Kuechler et al. 2013). Phylogenetic analysis indicated that the monophyletic group of P-endosymbiont peloridiids is strictly subdivided into well-formed subgroups in accord with their geographical distribution (e.g., endosymbionts of Australia and New Zealand species are clearly separated from each other). Furthermore, a phylogenetic concordance between the P-endosymbionts and their moss bugs hosts exists (Kuechler et al. 2013).

Here, we report the complete genome of the P-endosymbiont “*Ca.* Evansia muelleri” (hereafter referred to as *Evansia*) strain Xc1 (Gammaproteobacteria: Oceanospirillales: Halomonadaceae) from *Xenophyes cascus* strain OF (Hemiptera: Coleorrhyncha: Peloridiidae) (hereafter referred to as *Xenophyes* OF)*.* Its genome displays an extreme reduction, a low GC content, a high coding density, and the loss of several genes that are essentials for housekeeping functions. Despite its small genome size, *Evansia* retains one of the most complete gene sets for amino acid biosynthesis and sulfur metabolism. A comparative genomic analysis with the other Halomonadaceae endosymbionts of the order Hemiptera, *Portiera* and *Carsonella*, led us to propose a possible scenario for the origin of this new endosymbiotic clade.

## Materials and Methods

### Sampling and Genome Sequencing

Adult individuals of *Xenophyes* OF were collected in the field (Otaki Forks, Tararua Ranges, Tararua Forest Park, New Zealand), in January 2013. Subsequently, an ultrastructural analysis using transmission electron microscopy (for experimental procedure see Kuechler et al. 2013) and DNA isolation were carried out. Bacteriomes were dissected from 17 adult females and used for DNA extraction following manufacturer's instructions (PureLink Genomic DNA Mini Kit, Invitrogen). Six independent whole-genome amplifications runs (GenomiPhi v2, GE Healthcare) starting with 8 ng DNA were performed following the manufacturer’s instructions. Reaction was left for 1.5 h at 30 °C and inactivated at 65 °C for 10 min. Because DNA chimeric amplification seems to be a random process, samples were mixed for maintaining possible chimeras at a low ratio in relation to nonchimeric-amplified DNA. Amplified DNA was used for sequencing with Roche 454 GSFLX Titanium (single-end shotgun with 800 bp of average read length) and Illumina HiSeq2000 (350-bp paired-end library and 2 × 100 bp) platforms.

### Assembly and Annotation

Sequence read quality filtering was performed with custom python scripts and Perl scripts, whereas quality was checked with FastQC v0.10 after and before filtering. Approximately, 283,000 (454) and 29.3 million (Illumina) reads were generated after filtering.

An initial hybrid assembly with both kinds of reads was performed with MIRA v4.0 (Chevreux et al. 1999), and *Evansia* contigs were selected based on GC content, read coverage, BLAST similarities, and PhymmBL (Brady and Salzberg 2011). A single contig (150× 454 and 330× Illumina read coverages) containing the mitogenome of *Xenophyes* OF was also detected by a BLAST search against the published mitogenome of *X. cascus* (JF323862). Illumina and 454 reads were mapped against the assembled *Xenophyes* OF mitochondrion and excluded for the subsequent assembly steps. Initial *Evansia* selected contigs were used for a second round of PhymmBL training, and 454 reads were selected based on GC content, coverage, and PhymmBL results. Illumina reads were mapped against initial *Evansia* contigs. Selected 454 and Illumina reads were assembled with MIRA, and polisher (Foster et al. 2012) was used to correct the remaining homopolymers also with Illumina reads. Corrected assembly was used as input for SSPACE v2.0 for scaffolding (Boetzer et al. 2011), and gaps were filled with GapFiller v1.0 (Boetzer and Pirovano 2012) using Illumina reads for both programs. Finally, an iterative mapping approach with MIRA v4.0 and manually editing with gap4 (Staden et al. 2000) were used for closing the remaining gaps (Sloan and Moran 2012b). Polisher was run to correct possible homopolymers and indel errors in the finished genome.

RAST (Aziz et al. 2008) was used for a first step annotation. InterProScan (Quevillon et al. 2005) was used for annotation refinement followed by a manual curation of the genome. Rfam (Burge et al. 2013) was used to predict noncoding RNA genes, and TFAM (Ardell and Andersson 2006) was employed to predict tRNA genes. Cluster of orthologous clusters (COG) (Tatusov et al. 2000) were assigned with a set of custom Perl scripts (BLASTP *e*-value cutoff of 1e^−^^03^), and Circos (Krzywinski et al*.* 2009) was used to plot the genome overview. *Xenophyes* OF mitochondrion was annotated with MITOS (Bernt et al*.* 2013) and manually curated.

Pathway tools (Karp et al. 2002) in combination with BioCyc (Karp et al. 2005) and BRENDA (Schomburg et al. 2013) databases were used to infer and curate *Evansia* metabolic capabilities. The curated metabolism of *Evansia muelleri Xc1* in pathway tools (Ocelot format) can be supplied under request. Metabolism graph was done with yEd Graph Editor.

### Phylogenomics Analyses

Proteomes from completely sequenced Halomonadaceae were downloaded from GenBank database, including three *Carsonella* (DC, HC, and PV strains) and three *Portiera* genomes (BT-QVLC, BT-B, and TV strains). *Pseudomonas aeruginosa* B136-33 was also downloaded and used as outgroup. Proteomes were fed into PhyloPhlAn (Segata et al. 2013) for a preliminary phylogenomic reconstruction. Because symbionts lack most of the 400 markers genes used by PhyloPhlAn, only 23 of these genes that were present in all the Halomonadaceae genomes (including symbionts) were selected for a curated phylogenomic reconstruction. The 23 proteins were aligned with MAFFT (L-INS-i algorithm) (Katoh et al. 2002) and concatenated. Gblocks (Castresana 2000) was used to prune the concatenated alignment and used as input for ProtTest3 (Darriba et al. 2011). The Improved General Amino Acid Replacement Matrix, with gamma distributed rates across sites using empirical base frequencies (LG+G+F) was scored as the best evolutionary model by ProtTest3. RaxML (Stamatakis 2006) with branch length optimization and 1,000 rapid bootstrap replicates was used for maximum likelihood (ML) tree reconstruction. PhyloBayes3 (Lartillot et al. 2009) was used to perform Bayesian analysis of the ML tree under the specified model for the concatenated proteins. PhyloBayes3 was left to run 2,868 cycles (328,604 trees) in five simultaneous chains, and 250 cycles were discarded as “burn in,” giving a final number of 2,618 trees (with sample frequency of 500). Effective sizes for all inferred parameters were higher than 1,300, and convergence of the chains was checked. A majority rule consensus tree was recovered from selected trees.

Phylogenetic analyses under ML were also performed for each separate protein alignment as described above, but adjusting the evolutionary model for each case.

Proteomes of complete mitochondria from Heteroptera, Coleorrhyncha, Auchenorrhyncha, and Sternorrhyncha were downloaded from GenBank database. Proteins encoded by ten genes (*COX1-3*, *ND1-4*, *ND5*, *cytB,* and *atp6*) for all mitochondria, including *Xenophyes* OF, were selected, and the best evolutionary model for each one was predicted with ProtTest3. MtArt model with gamma distribution was the best model for all the proteins under the Bayesian Information Criterion. RaxML and PhyloBayes3 were run as described above, adjusting the evolutionary model and partitions for each protein. PhyloBayes3 was run with five independent chains and stopped at 1,000 cycles (228,952 trees). Convergence of the chains and valid effective sizes were checked. First 250 cycles were discarded as “burn in.” Finally, a majority rule consensus tree was obtained.

### COG Clustering Analysis

COG (cluster of orthologous groups) profiles for different symbionts were downloaded from the IMG (Markowitz et al. 2014) database (supplementary table S1, Supplementary Material online). COG categories for *Portiera* TV strain were assigned as described above. A count matrix was generated from COG profiles, and clustering for different COG categories was performed with R (R Development Core Team 2014), under a binary distance model and a complete clustering method. Heatmap and clustering dendograms were plotted with gplots (Warnes et al. 2013).

### Orthologous Gene Identification

*Chromohalobacter salexigens* DSM 3043, *Halomonas elongata* DSM 2581, *Carsonella* (strains *DC, HC,* and *PV*), *Portiera* (strains BT-B, BT-QVLC, and TV), and *Evansia* proteomes were used as input for OrthoMCL (Li et al. 2003) and run as described previously (Manzano-Marín et al. 2012). OrthoMCL output clusters were manually inspected and refined due to the failure to assign some proteins from *Carsonella* to the correct cluster (based on BLASTP results and gene name or protein function). An Euler diagram for orthologous clusters was plotted with gplots package from R.

### Genome Rearrangements

A total of 150 orthologous clusters shared by all genomes were selected for rearrangement analysis. MGR (Bourque and Pevzner 2002) was used to calculate the minimum number of rearrangements needed to explain the differences in the genomic architecture between the selected genomes. Phylogenetic reconstruction based on rearrangements were made with TIBA (Lin et al. 2012), using a neighbor joining method with 100 bootstrap replicates.

## Results

### Genomic Features

**Fig. 1.—:**
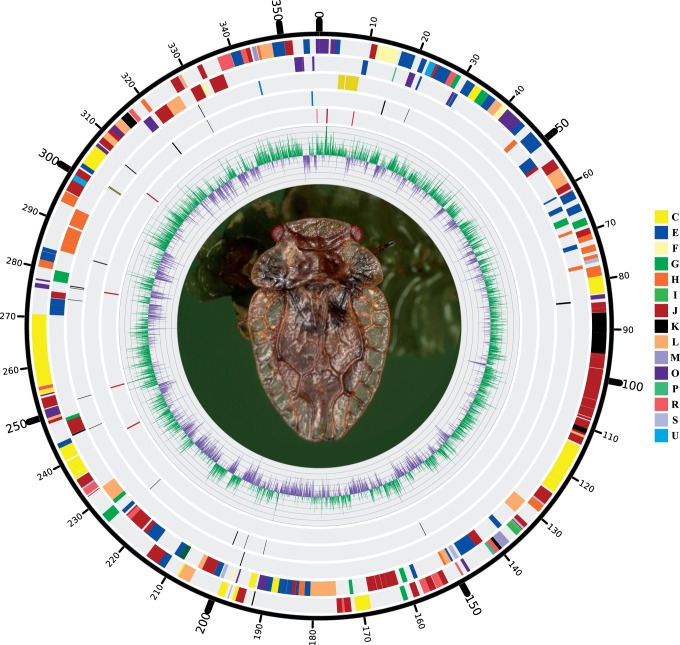
Circular view of *Candidatus* Evansia muelleri genome*.* From inner to outer tracks: (I) Positive (green) and negative (purple) GC skew across the genome. (II) Tandem repeats (red lines). (III) Complementary strand noncoding RNA genes: rRNA genes (yellow), transfer RNA genes (black), other RNA genes (blue). (IV) Direct strand noncoding RNA genes: rRNA genes (yellow), transfer RNA genes (black), other RNA genes (blue). (V) Complementary strand CDS. (VI) Direct strand CDS. CDS were colored according to cluster of orthologous groups (COG) classification. The background image is a picture of *Evansia*'s host *Xenophyes cascus*.

**Fig. 2.—:**
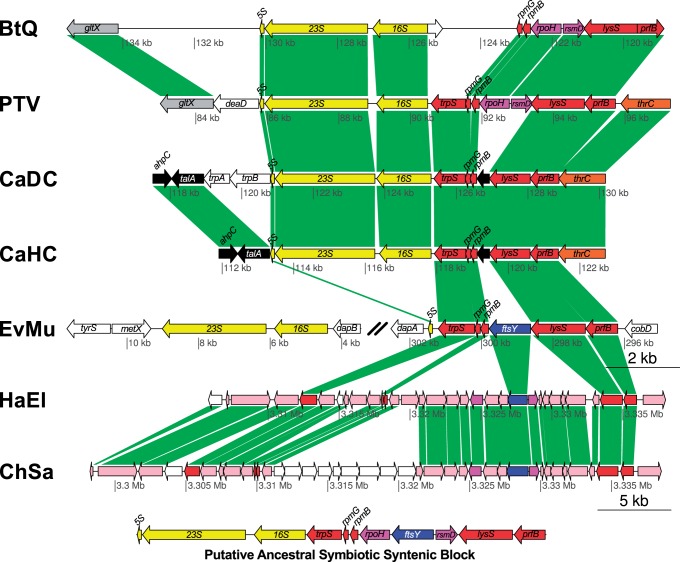
Genome context of rRNA operons. The single complete rRNA operon of *Portiera* strains BT-QVLC (BtQ) and TV (PTV) and *Carsonella* strains DC (CaDC) and HC (CaHC), and the two rRNA transcription units in *Evansia* (EvMu) are shown. The putative ancestral symbiotic syntenic block that includes the rRNA operon retained in *Carsonella* and *Portiera* is shown at the bottom. The genome context of the upstream genes in *Chromohalobacter salexigens* (ChSa) and *Halomonas elongata* (HaEl) is also shown. Homologous genes are tagged with several colors. White genes represent genes without homologs in the displayed chromosomal region. The chromosomal positions of the displayed segments are shown under each drawing.

After quality filtering, symbiont read selection, assembly (253× 454 and 557× Illumina coverages), and correction of homopolymers, a single contig for the *Evansia* chromosome was obtained. *Evansia* presents a small genome of 357,498 bp composed of a single circular chromosome, a low GC content, close to 25%, and a coding density of 93.7% (fig. 1, table 1). A total of 369 genes were detected (330 protein coding and 39 noncoding RNA genes), which is a higher number than those of its closest symbiont relatives *Portiera* and *Carsonella* (table 1 and supplementary table S1, Supplementary Material online). The genome contains a complete set of tRNA genes (able to translate all codons, which include the initiator formyl methionine [MET] and the lysylated isoleucine [ILE] tRNA), three noncoding RNA genes (*rnpB*, a putative sRNA sX4, and *ssrA*), and one ribosomal RNA (rRNA) gene of each type. In contrast to *Portiera* and *Carsonella*, the rRNA operon was split in two segments, one containing the 16S and 23S rRNAs and the other the 5S rRNA. The rRNA operons of *Carsonella* and *Portiera,* as well as the 5S rRNA from *Evansia,* seem to be orthologous because they have the same upstream genomic context, suggesting an ancestral syntenic block, which includes the *trpS, rpmG*, *rpmB*, *rpoH*, *ftsY*, *rsmD*, *lysS,* and *prfB* genes (fig. 2).

**Table 1:** Main Genomic Features of Halomonadaceae Endosymbionts

Symbiont	Genome Size (bp)	GC (%)	Genes	CDS	Coding Density (%)	rRNA	tRNA	Other RNA	Pseudo
*Candidatus* Carsonella ruddii PV	159,662	17	213	182	97	3	28	0	0
*Ca.* Carsonella ruddii HC	166,163	14	223	192	98	3	28	0	0
*Ca.* Portiera aleyrodidarum TV	280,663	25	307	269	92	3	34	1	0
*Ca.* Portiera aleyrodidarum BT-QVLC	357,472	26	284	246	68	3	33	2	8
*Ca.* Evansia muelleri Xc1	357,498	25	369	330	94	3	33	3	0
*Buchnera aphidicola* Cc^a^	422,434	20	403	365	87	3	31	4	3
*Bu. aphidicola* 5A	642,122	27	592	555	87	3	32	2	7

Note.—For comparative purposes, two strains of the endosymbiont of aphids, *Bu. aphidicola* were included.

^a^Plasmid pLeu-BCc is included in the summary statistics.

*Evansia* also contains eight tandem repeats, ranging from 100 to 330 bp, distributed along the genome (fig. 1). This type of repeat has also been reported and proposed as a possible explanation for the genomic instability of *Portiera* from *Bemisia tabaci* (Sloan and Moran 2013).

The genome of *Evansia* does not encode all the proteins required for the basic DNA and RNA metabolisms (Gil et al*.* 2004). It lacks the DNA polymerase subunit encoding genes *holA, holB,* and *dnaX* from the basic replication machinery and the *argS* gene from the aminoacyl-tRNA synthetases*.* It also lacks *asnS* (asparaginyl-tRNA synthetase), but the synthesis of Asn-tRNA could be performed by a nondiscriminating aspartyl tRNA synthetase (*aspS*) (Charron et al. 2003) coupled to GatABC aspartyl/glutamyl-tRNA amidotransferase reaction.

### Cell Morphology

**Fig. 3.—:**
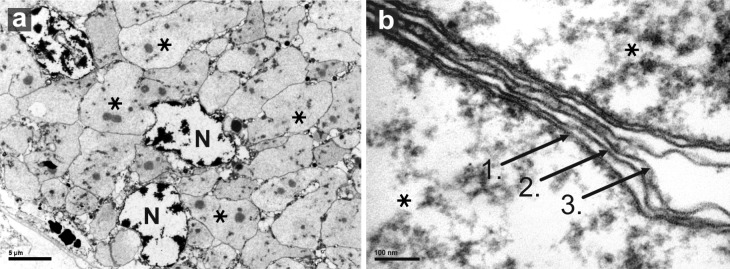
Transmission electron microscopy micrographs of *Candidatus* Evansia muelleri membranes. (*a*) Bacteriome overview. (*b*) Three membrane system in *Evansia*: 1. inner membrane; 2. outer membrane; and 3. host-derived membrane. *Evansia* cells (*). Host nuclei (N).

*Evansia* cells are large, pleomorphic, and has nucleoid-like masses (electron-dense granules) similar to *Carsonella* and *Portiera* (Costa et al. 1993; Baumann 2005; Kuechler et al. 2013) occupying most of the bacteriocyte cytosol. Interestingly, although *Evansia* does not encode genes for the fatty acid metabolism (only *fabF* is present), it shows a three-membrane system (fig. 3), one derived from the host and the two typical Gram-negative bacterial membranes (inner and outer membranes).

### Phylogenomic Reconstruction

**Fig. 4.—:**
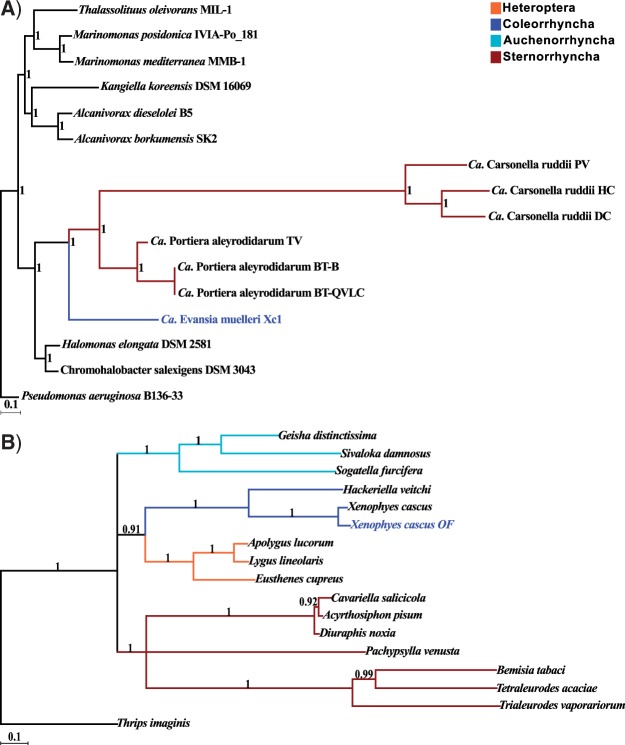
Host and symbiont phylogenies. (*A*) Phylogenomic reconstruction for sequenced Halomonadaceae genomes. Majority rule consensus tree for the 23 concatenated genes from Bayesian analysis is displayed. Posterior distributions are displayed at each node. *Pseudomonas aeruginosa* was used as outgroup. *Evansia* is displayed in blue. (*B*) Phylogenomic reconstruction for different hemipteran mitochondrial genomes. Majority rule consensus tree for the 23 concatenated genes from Bayesian analysis is displayed. Posterior distributions are displayed at each node. *Thrips imaginis* (Thysanoptera) was used as outgroup. *Evansia* host (*Xenophyes cascus* OF) is displayed in blue.

Initial ML phylogenomic reconstruction performed with PhyloPhlAn included *Evansia* within a symbiont clade with *Portiera* and *Carsonella* as recently proposed based on a 16S rRNA phylogeny (Kuechler et al. 2013). The phylogenomic reconstruction gave a different topology, with *Evansia* as the first divergent lineage and *Portiera* and *Carsonella* more closely related (data not shown). To solve the disagreement with the previously published phylogeny, ML phylogeny reconstructions were performed with a subsample of 23 groups of orthologous proteins and with its concatenate alignment (supplementary file 1: supplementary fig. S1, Supplementary Material online). Sixteen single-protein phylogenies placed *Evansia* outside the *Portiera/Carsonella* cluster (100% median bootstrap), whereas seven located *Evansia* as the closest relative of *Carsonella* (76% median bootstrap confidence) (as in the 16S rRNA phylogeny) (see supplementary file 2, Supplementary Material online). Concatenated protein ML phylogeny supported also the most frequent gene phylogeny with a 100% bootstrap value (supplementary file 1: supplementary fig. S1, Supplementary Material online). This phylogeny was also reconstructed with a Bayesian analysis performed on the concatenated alignment with same results (fig. 4*A*). The fact that two topologies were obtained using 23 genes could suggest that *Evansia* has some genome heterogeneity. The presence of ancestral duplicated genes and the differential loss of paralogs in each lineage could be a reason for this heterogeneity, but an artifactual topology due to the rapid sequence evolution in extreme reduced genomes could not be ruled out. Indeed, rRNA genes in *Evansia* seem to derive from different rRNA clusters if we look at their genomic contexts (fig. 2). The above considerations could be the reason our updated phylogeny differs from the previously published one. Also, a phylogenetic Bayesian inference was performed for the mitochondrial proteomes of different Heteroptera, Coleorrhyncha, Auchenorrhyncha, and Sternorrhyncha (fig. 4*B*). Although only three rooted topologies are possible with equal probabilities, it should be noted that the *Evansia*, *Portiera,* and *Carsonella* topology is concordant with the one of their hosts.

### *Comparative Analysis of* Evansia *Proteins Based on COG Functional Categories*

**Fig. 5.—:**
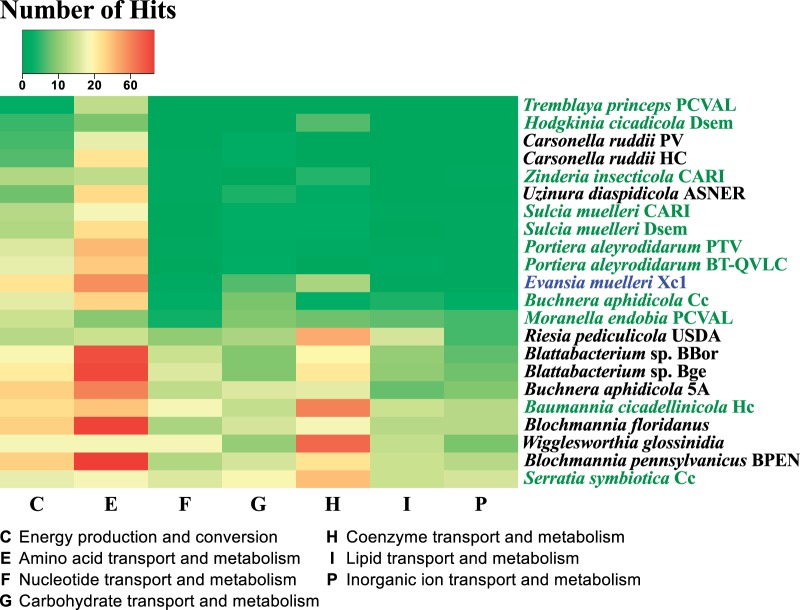
Heatmap of selected COG categories from different endosymbionts. Endosymbionts are sorted by genome size from the smallest (*Tremblaya princeps* PCVAL) to the biggest (*Serratia symbiotica* Cc). For each genome, the numbers of hits in each COG category are shown. P-endosymbionts living with another co-P-endosymbiont are displayed in green. *Evansia muelleri* Xc1, which has no co-P-endosymbiont, is displayed in blue. *Serratia symbiotica* Cc is an example of a coprimary symbiont of large genome. COG descriptions are showed in the bottom.

The distribution of proteins in COG categories was compared with those of other insect P-endosymbionts (fig. 5). *Evansia* falls into the category of P-endosymbionts that do not require another coprimary symbiont for its mutualistic action. The **C** (energy production) category showed more hits in *Evansia* than in other endosymbionts with small genome sizes like *Portiera*, *Buchnera aphidicola* BCc, *Moranella,* or *Hodgkinia.* In fact, clustering analysis for this category showed that *Evansia* was closer to *Blochmannia* spp., the endosymbionts of carpenter ants with genomes of 700–800 kb (supplementary file 1: supplementary fig. S2, Supplementary Material online). E (amino acids biosynthesis) category also showed a slight increase in *Evansia* when compared with endosymbionts with similar genome size and the clustering analysis confirmed that *Evansia* was closer to *Blattabacterium*, *Bu. aphidicola* strain 5A, and *Blochmannia* that are able to almost completely synthesize all the essential amino acids (supplementary file 1: supplementary fig. S3, Supplementary Material online). Finally, H (coenzyme metabolism) category also showed an increase when it was compared with the extreme reduced endosymbionts with the exception of *Hodgkinia*. In this case, *Hodgkinia* and *Evansia* clustered together, pointing to a similar role in vitamin biosynthesis (supplementary file 1: supplementary fig. S4, Supplementary Material online). The remaining COG categories did not show clear differences with the endosymbionts of similar genome sizes. These data suggest that even though *Evansia* possesses a much reduced genome, it has some interesting features similar to endosymbionts with larger genomes.

### *Metabolic Capabilities of* Evansia

**Fig. 6.—:**
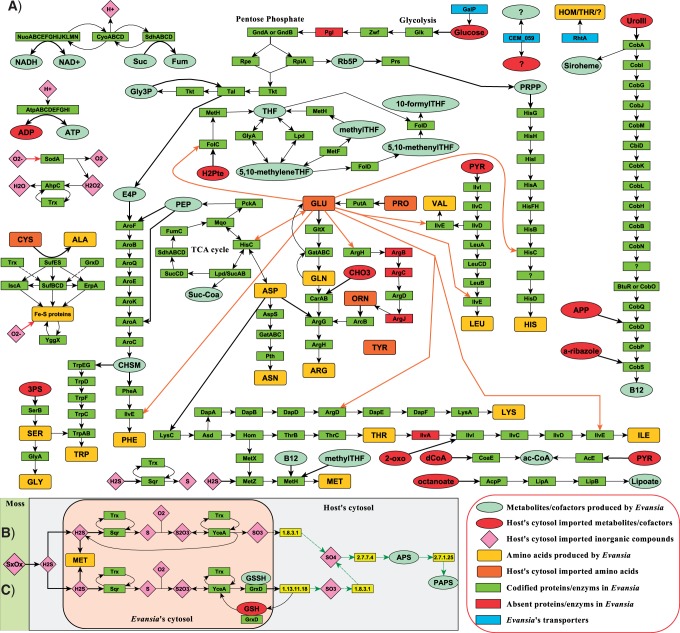
Biosynthetic capabilities of *Evansia*. (*A*) Simplified graph showing the metabolic reconstruction for *Evansia*. (*B*) Putative H_2_S recycling pathway. (*C*) Putative H_2_S detoxification pathway. Amino acids are identified by the standard three letter code. Black arrows point the direction of the reaction. Black thick arrows represent inputs for a reaction. Orange thick arrows indicate reactions that need glutamate as input. Red arrows represent ROS interactions. Green arrows denote reactions produced by the host. Dashed arrows indicate poorly characterized or putative reactions. “?” denotes an unidentified protein/metabolite/compound. APP, (R)-1-amino-2-propanol O-2-phosphate; UroII, uroporphyrinogen-III; CHO3, bicarbonate; PYR, pyruvate; CHSM, chorismate; E4P, D-erythrose 4-phosphate; Rb5P, D-ribose 5-phosphate; PRPP, 5-phospho-α-D-ribose 1-diphosphate; Gly3P, D-glyceraldehyde 3-phosphate- H2Pte, 7,8-dihydropteroate; THF, tetrahydrofolate; CoA, coenzyme A; dCoA, 3'-dephospho-CoA; Ac-CoA, acetyl-CoA; Suc-Coa, succinyl-CoA; PEP, phosphoenolpyruvate; 3PS, 3-phospho-L-serine; O2−, reactive oxygen species; 2-oxo, 2-oxoglutarate; GSH, glutathione; GSSH, glutathione sulfide; APS, adenosine 5'-phosphosulfate; PAPS, phosphoadenosine-5'-phosphosulfate; SxOx, Sulfur oxidized forms.

The full metabolism of *Evansia* was inferred, and its biosynthetic capabilities were represented as a simplified graph (fig. 6*A*–*C*). *Evansia* showed an incomplete glycolytic pathway and an almost complete pentose phosphate pathway (the function of the absent *pgl* gene could be complemented by the host). The presence of a functional pentose phosphate pathway would permit *Evansia* to produce different metabolites, which are used as input for other pathways and reducing power (NADPH). *Evansia* also possesses a reduced TCA cycle, able to produce all necessary metabolites, with HisC producing a bypass from oxalacetate to 2-oxoglutarate and making dispensable the three first steps of the cycle (encoded by *gltA, acnAB,* and *icd*) as it occurs in some *Blattabacterium* strains (González-Domenech et al. 2012).

Regarding the synthesis of amino acids, *Evansia* is able to produce alone, or complemented by the host, the ten essential ones. It harbors the full pathways for the seven essential amino acids leucine, valine, lysine, tryptophan, phenylalanine, threonine (THR), and arginine (ARG). It contains almost complete pathways for ILE and histidine. In the case of ILE, the pathway lacks IlvA and the regulatory subunit IlvH, but IlvI maintains a reduced catalytic activity in its absence (Vyazmensky et al. 1996). Finally, the MET biosynthetic pathway is very unusual regarding other P-endosymbionts. The synthesis is carried out by a sulfhydrylation step (encoded by *metX* and *metZ*) and finished by the cobalamin-dependent MET synthase (encoded by *metH*). This alternative pathway is probably of ancestral origin because it is also found in species of the genus *Pseudomonas* (Alaminos and Ramos 2001). *Evansia* needs to import *S*-adenosyl-MET from the host. Concerning nonessential amino acids, *Evansia* is able to synthesize alanine, asparagine, aspartate, glutamine, glycine, and serine, whereas it needs to import glutamate, proline, ornithine, cysteine (CYS), and tyrosine from the host.

*Evansia* can also produce different cofactors and intermediate metabolites importing different compounds from the host's cytosol. Among these cofactors, we can found acetyl-CoA, lipoate, and different folate forms. *Evansia* almost maintains the full cobalamin (B12) biosynthesis pathway, but although one enzyme was not identified, the presence of the remaining enzymes points to this enzyme activity is encoded in the genome. The missing enzyme, which produces the reduction from Cob(II)yrinic acid a,c-diamide to Cob(I)yrinic acid a,c-diamide, is still under some controversy (revised in Mera and Escalante-Semerena 2010), and some candidates could be present in *Evansia*.

Reductive power (NADH) and energy (ATP) can be produced by the NADH-quinone oxidoreductase and the ATP synthase complexes, respectively. In contrast, *Evansia* is not able to produce purines and pyrimidines, so they would have to be imported from the host. *Evansia* only conserves the *ribH* gene from the flavin biosynthesis pathway, so all its derivatives (riboflavin, FMN, and FAD) would also have to be imported from the host cytosol. In addition, *Evansia* also needs to import NAD.

Regarding the sulfur metabolism, *Evansia* seems to have the ability to transform hydrogen sulfide (H_2_S) to intracellular sulfur (S) and vice versa by a sulfide:quinone oxidoreductase (SQR; encoded by *sqr*) coupled to thioredoxin (encoded by *trx*). Fe-S proteins could be assembled by the A-type carriers (encoded by *erpA* and *iscA*), the CYS desulfurase (SufES), and the SufBCD complex, whereas YggX is in charge of repair Fe-S proteins from superoxide damages (Gralnick and Downs 2001).

Reactive oxygen species (ROS) and their derivatives (O_2_-) can be reduced to water by the combination of *sodA*, *ahpC,* and *trx* gene products (Parsonage et al. 2008).

Finally, *Evansia* genome encodes two transporters involved in the metabolism: GalP that is involved in the glucose import and RhtA, a homoserine/theonine efflux transporter that can also export other amino acids (Hernández-Montalvo et al. 2003; Livshits et al. 2003). A third transporter (encoded by *CEM_059*), which seems to be distantly related to DitE transporter from *P. abietaniphila*, was identified (Martin and Mohn 2000). This transporter may have a different function, because no diterpenoids degrading enzymes were found in *Evansia*. As it belongs to the major facilitator superfamily, it could act on a wide number of substrates but its function remains unknown.

### Comparative Genomics between *Evansia* and Its Relatives

Orthologous clusters were generated for *C. salexigens* DSM 3043, *H. elongata* DSM 2581, *Carsonella* pangenome (genes present in any of the strains DC, HC, and PV), *Portiera* pangenome (genes present in any of the strains BT-B, BT-QVLC, and TV), and *Evansia*. If the free-living relatives (*Chromohalobacter* and *Halomonas*) were included, the Euler diagram subspaces of the orthologous clusters gave a core genome of 158 clusters (supplementary file 1: supplementary fig. S5, Supplementary Material online). *Chromohalobacter* and *Halomonas* showed 884 and 1,026 strain-specific genes, respectively, and 1,978 shared genes. Based on the Euler diagram, it seems that endosymbiont gene repertoires are just a reduced subset from the free-living ones without new genes acquired after the divergence of symbiotic lineages. Although *Carsonella* pangenome contains 54 strain-specific genes (most of them hypothetical proteins), this number could be an artifact due to the problematic assignment to orthologous clusters of several *Carsonella* genes owing to its high evolutionary divergence. Similarly, in *Portiera* pangenome, 16 genes encoding small hypothetical proteins could be artifacts (supplementary file 1: supplementary fig. S5, Supplementary Material online). The specific carotenoid biosynthetic genes, although absent in *Chromohalobacter* and *Halomonas,* are encoded in the genomes of other free-living Halomonadaceae, ruling out a case of horizontal gene transfer (HGT) in the symbiotic lineage. Finally, also discarding some hypothetical proteins without recognizable domains in *Evansia*, the two other remaining genes were probably of ancestral origin because they are present in other Oceanospirillales species.

**Fig. 7.—:**
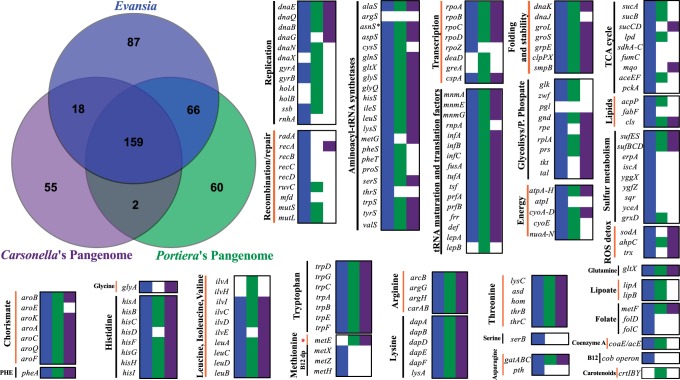
Orthologous coding gene clusters from *Evansia*, *Carsonella*'s Pangenome, and *Portiera*'s Pangenome. Euler diagram displaying the number of clusters found on each subspace of the pangenome (top left). Gene contents for the most relevant cell functions, and metabolic capabilities are plotted for each endosymbiont. Filled squares indicate the presence of genes in *Evansia* (blue), *Carsonella*’s pangenome (purple), and *Portiera*’s pangenome (green). Absent genes (white). *Carsonella*'s and *Portiera*'s pangenomes include the CDS from *Carsonella* strains DC, HC, and PV and *Portiera* strains BT-B, BT-QVLC, and TV, respectively. B12: cobalamin. Red star denotes the MET cobalamin-independent pathway. B12 dp denotes the MET cobalamin-dependent pathway. The asterisk besides *asnS* denotes that, although none of the endosymbiont genomes harbors this gene, they may produce l-asparaginyl-tRNA with a nondiscriminating *aspS* in combination with *gatABC*. Each gene was plotted only once, although it was involved in two or more categories.

An Euler diagram was also generated for the three endosymbionts (fig. 7). In this case, the core genome is composed of 159 genes: Replication machinery*,* transcription machinery*,* part of the Fe-S cluster assembly proteins (and other chaperones/protein turnover), energy production, most of the essential amino acid metabolism, ribosomal genes (45 genes), and aminoacyl-tRNA synthetases, among other genes (fig. 7). *Carsonella*'s pangenome and *Evansia* present a more complete TCA cycle and pentose phosphate pathway than *Portiera,* whereas *Evansia* has retained a more complex energy production machinery. *Evansia* shows a different pathway for MET synthesis (*metHXZ* and cobalamin biosynthetic genes) than *Portiera* and *Carsonella* (*metE*). Although *Carsonella* is not able to produce cofactors, *Evansia* and *Portiera* are able to synthesize some of them. In contrast, only *Portiera* is able to synthesize carotenoids. The homologous recombination machinery is only complete, and probably active as can be seen by the absence of a clear GC skew, in *Evansia* (fig. 1) that is an unusual characteristic in extreme reduced endosymbionts (fig. 7). Although the three symbionts maintain membrane proteins such as the ATPase machinery and some transporters, related to the Fe-S cluster assembly (*sufBC*), or chaperones (*clpX*), *Evansia* presents three additional transporters, two shared with *Portiera* (GalP and the DitE related transporter *CEM_059*) and one related to the extrusion of different amino acids (encoded by *rthA*). *Carsonella* does not present any other transporter. *Evansia* also conserves three Sec-dependent pathway proteins (encoded by *ftsY, ffh,* and *secE*, the latter perhaps a pseudogene) and a protein in charge to remove signal peptides (*lspA*). Two of them are also present in *Portiera* (*secE* and *lspA*).

Among the outstanding gene sets of *Evansia* are those involved in sulfur metabolism, including the common Fe-S cluster assembly proteins SufBCES and the SufD protein shared with *Portiera*. Also, *Evansia* encodes four proteins directly related to the Fe-S cluster assembly (*iscA*, *yggX*, *ygfZ,* and *erpA*) and three proteins probably involved in sulfur mobilization (*sqr, yceA,* and *grxD*).

Lastly, *Evansia* and *Carsonella* seem to be able to reduce ROS to water (*sodA* and *ahpC*). See supplementary table S2, Supplementary Material online, for a detailed list of the endosymbiont Euler diagram (fig. 7).

### Genome Rearrangements Analysis

**Fig. 8.—:**
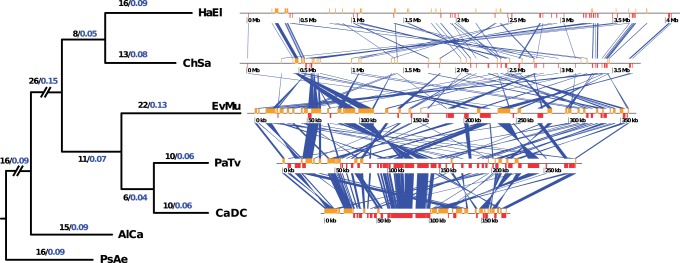
Evolution of genome rearrangements in *Evansia* and its relatives. Rooted tree showing the neighbor joining rearrangement phylogeny (TIBA) (left). Black numbers at each node are the minimum number of rearrangements needed for explaining the genomic order at each node (MGR), whereas blue numbers are relative values (the number of rearrangements in each branch/total number of rearrangements in the tree). Branches out of the study scope were shortened (slashes). Branch lengths are proportional to the number of rearrangements. On the right, blue bands connect orthologous genes between analyzed genomes. HaEl, *Halomonas elongata* DSM 2581; ChSa, *Chromohalobacter salexigens* DSM 3043; EvMu, *Candidatus* Evansia muelleri Xc1; PaTv, *Ca.* Portiera aleyrodidarum strain TV; CaDC, *Ca.* Carsonella ruddii strain DC; AlCa, *Alcanivorax borkumensis* SK2; PsAe, *Pseudomonas aeruginosa* B136-33.

The analysis performed with 150 core clusters showed a total of 27–33 rearrangements required to explain the changes in the genomic architecture from the ancestral free-living relative (the most recent common ancestor with *H. elongata* and *C. salexigens*) to the three endosymbionts (fig. 8). The lineage of *Portiera* from *B. tabaci* was excluded from the analysis because the high number of rearrangements occurred in this strain after its divergence from *Portiera* of *Trialeurodes vaporariorum* (Sloan and Moran 2013). Our rearrangement-based phylogeny is congruent with the sequence-based phylogeny (fig. 4), thus corroborating the topology of the symbiotic clade with other phylogenetic approach.

During the same evolutionary time, the numbers of rearrangements in the branches leading to the free-living *C. salexigens* and *H. elongata* were only slightly smaller than those leading to the symbiotic clade, revealing that in comparison with some other symbionts such as *Bu. aphidicola* or *Wigglesworthia glossinidia* (Belda et al*.* 2005), there was not a high rearrangement rate during the initial steps of symbiosis.

## Discussion

### A Common Origin for Halomonadaceae Endosymbionts

The close phylogenetic relationship of P-endosymbionts of whiteflies and psyllids was previously observed by several authors (Thao and Baumann 2004; Sloan and Moran 2012b; Sloan and Moran 2013). In this work, we report the first genome of a Peloridiidae (Coleorrhyncha) P-endosymbiont, *Ca.* Evansia muelleri, which was recently proposed as a close relative of *Portiera* and *Carsonella* (Kuechler et al. 2013)*.* Our phylogenomic reconstruction showed that the three endosymbionts form a well-supported monophyletic group included in the family Halomonadaceae (Gammaproteobacteria) (fig. 4*A*) as reported in a previous study based on 16S rRNA gene sequences (Kuechler et al*.* 2013). However, the relative position of the three endosymbionts was different with *Evansia* being the basal lineage when a phylogenomic approach was performed as explained above.

Although the obtained endosymbiont topology is concordant with the topology of their insect hosts (Coleorrhyncha, Psylloidea, and Aleyrodoidea) (fig. 4*B*), it is necessary to take this result with caution because the low number of host and endosymbiont taxa included. Moreover, the phylogenetic relationships with other members of the order Hemiptera are still under discussion (Cryan and Urban 2012; Song et al*.* 2012). However, paleontological evidence has served to propose the group Psyllinea, which would include superfamilies Psylloidea and Aleyrodoidea in opposition to Aphidinea (including Aphidoidea and Coccoidea) (Shcherbakov 2000).

When reconciling the evolutionary histories of P-endosymbionts and their hosts, three scenarios are possible: 1) a single event of host–symbiont association in the ancestor of all Hemiptera (>250 Ma); 2) two infection events, one at the base of Psyllinea clade and another at the base of the Peloridiidae; 3) three infection events, one for Psylloidea, one for Aleyrodoidea, and one for Peloridiidae followed by the coevolution of host and endosymbiont in each lineage

There are some clues that can help us to understand the origin of this endosymbiotic clade: 1) The concordance between the host and P-endosymbiont phylogenies (fig. 4), 2) the retention of the same rRNA operon in *Portiera* and *Carsonella* and partially in *Evansia* (fig. 2), 3) the extremely reduced genomes of these P-endosymbionts, indicative of a very long endosymbiotic relationship (table 1), 4) the ancient origin of all the endosymbiont retained genes (discarding those encoding for hypothetical proteins). They are subsets of the Halomonadaceae free-living bacteria, and HGT events are not found in the symbiotic lineages (supplementary fig. S5, Supplementary Material online), and 5) the fixation of different MET biosynthetic pathways in their reduced genomes.

**Fig. 9.—:**
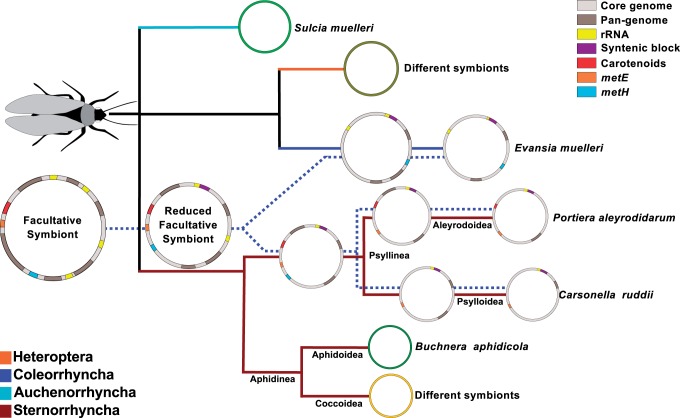
Evolutionary histories of P-endosymbionts and insect hosts. Model of reconciliation of host and P-endosymbiont phylogenies based on the hypothesis of two events of host–symbiont association followed by coevolution. Each Hemiptera suborder is marked by a different solid colored line. Endosymbiont phylogeny is shown with a dotted line. In Sternorrhyncha suborder, phylogenetic relationships are based in Shcherbakov (2000). Parallel dotted and solid lines mean host–symbiont coevolution.

As free-living bacteria usually harbor multiple rRNA operons (four and five in *Halomonas* and *Chromohalobacter*, respectively), the observation that the only rRNA operon retained in *Carsonella* and *Portiera* is ortholog suggests that their most recent common ancestor already had a partially reduced genome (fig. 2). To explain the formation of this syntenic block, a chromosomal rearrangement moving an rRNA operon adjacent to the *trpS*/*prfB* block, besides several gene losses during the process of genome reduction, is required (fig. 2). This rearrangement took place before the divergence of *Evansia* from *Carsonela/Portiera* as shown by the presence of the *5S rRNA* gene in *Evansia*. The distribution of rRNA genes in two transcription units in *Evansia* may be explained by one or more rearrangement events moving the *16S* and *23S rRNA* genes outside the *rRNA*/*prfB* block or by the presence of two rRNA operons at the time when *Evansia* and *Portiera-Carsonella* lineages diverged. None of the two hypotheses may be discarded. However, both would suggest the idea of a facultative symbiont with a genome at the beginning steps of the genome reduction process, because to explain the present genomic features of the three endosymbionts, either rRNA gene losses and/or rearrangements are required. Because a genomic stability is commonly obtained once a stable obligate endosymbiont has evolved (Tamas et al*.* 2002; Silva et al*.* 2003), the common origin of *Portiera* and *Carsonella* implies that their last common ancestor, although reduced in genome size, was already able to rearrange its genes (figs. 8 and 9). Although genomic architecture favors two infection events as the most likely scenario, the possibility of the single or the three infection events scenarios cannot be fully discarded.

The metabolism of the three endosymbionts also would support the two infection events hypothesis. Although *Carsonella* and *Portiera* (as many other endosymbionts) have maintained the cobalamin-independent MET biosynthesis pathway (*metE*) (Hansen and Moran 2014), *Evansia* has retained the dependent one (*metH*). Because the genes encoding these functions were present in the symbiotic ancestor, we can speculate that, at the time of the divergence of *Evansia* from the two other symbionts, the ancestral genome was partially reduced, and the fixation of different MET biosynthetic systems took place during the last steps of the reductive process together with the events of obligate associations of *Evansia* by one way, and the ancestor of *Portiera*/*Carsonella* by the other.

The single-event hypothesis would have implied the replacement of the ancestral symbiont in several lineages (Heteroptera, Auchenorrhyncha, and Aphidoidea), because some hemipterans harbor other primary endosymbionts (Baumann 2005; Hansen and Moran 2014). However, the replacement of an ancient obligate symbiont by a new more efficient one has been reported several times in insects (Conord et al*.* 2008; Koga et al. 2013; Oakeson et al*.* 2014). Overall, the single-event hypothesis seems less plausible than the two infection events from a parsimonious point of view.

In support of the alternative three event hypothesis, it could be argued that concordant phylogenies and retention of the same rRNA operon could have been produced by chance, and that, although extremely reduced genomes require a long coevolutionary relationship with their hosts, they might be produced in a shorter period of time (i.e., <200 Myr). In this three event hypothesis, the bacteria that started the obligate associations with the ancestors of each of the three insect lineages would come from a facultative endosymbiont clade able to infect different Hemiptera (or arthropod) lineages, as it has been reported for *Wolbachia*, *Hamiltonella*, *Rickettsia*, *Cardinium*, etc. (White 2011). This reasoning leads us to consider the two infection events hypothesis as the more plausible (fig. 9).

In conclusion, there is no doubt that an ancestral insect endosymbiont clade was able to infect ancestral Hemiptera, but the remaining question is whether the shift to only vertical transmission and to the obligate association took place one, two, or three times. To distinguish between them, the sequencing of the endosymbionts of other lineages and a better understanding of the phylogeny of Hemiptera will be required.

### Differences and Similarities between Halomonadaceae Endosymbionts

The genomic features of *Evansia*, *Portiera,* and *Carsonella* reveal the consequences of a long obligate endosymbiosis. Recently, the term symbionelle (Reyes-Prieto et al*.* 2013) was proposed to describe bacteria that fail to reach the minimal gene set (Gil et al*.* 2004). Although the number of genes in *Portiera* (circa 290) and *Evansia* (369) was a little higher that the threshold proposed for symbionelle, both may be considered symbionelles because they have lost some of the gene products required for a minimal cell to be autonomous. In the case of *Evansia*, the loss of *argS*, the gene encoding ARG-tRNA synthetase, but the retention of the noncoding gene *tRNA-Arg* suggests that a nuclear-encoded protein of unknown origin should be targeted to the bacterial cytosol to charge with ARG the tRNA-Arg. This *argS* gene could have been acquired through an HGT event either from *Evansia* or from a secondary endosymbiont. It could also be that the protein imported by mitochondria could also be imported by *Evansia*. HGT events from multiple bacteria to the nuclear genome have been recently observed in the mealybug *Planococcus citri* (Husnik et al*.* 2013). Also, recently, the import of several proteins of the photosystem I into the nascent photosynthetic organelles of the amoeba *Paulinella chromatophora* has been demonstrated (Nowack and Grossman 2012) suggesting that these systems may evolve frequently.

The cell structure of the three endosymbionts displays some important differences. Although *Carsonella* and *Evansia* present the usual three-membrane system (host–outer membrane-inner membrane) (fig. 3), *Portiera* lacks the outer membrane (Costa et al*.* 1993; Baumann 2005). In contrast, *Portiera* pangenome presents the largest number of transporters (11), whereas *Evansia* only presents three and *Carsonella* pangenome none. According to the Transporter Classification Database (www.tcdb.org, last accessed April 20, 2014), four (PalTV_015, 099, 174, and 204) out of the nine transporters found in *Portiera* TV are usually localized in both parts of the membrane (outer and inner). Thus, a closer look to *Portiera* cell structure needs to be revisited, because even *Carsonella*, the most reduced endosymbiont, also presents the three-membrane system. In addition, the way of membrane assembly in *Evansia* and *Carsonella* is still poorly understood. However, it is possible that these genes have been transferred to the host genome (Husnik et al. 2013; Sloan et al*.* 2014) and that the cytoplasm-synthesized proteins were incorporated to the bacterial membrane.

The most relevant characteristic of the metabolism of *Evansia* is that, in spite of its small genome size, it has retained, comparatively to other endosymbionts of similar or bigger sizes, a large set of the genes required for the synthesis of amino acids and cofactors, the metabolism of sulfur, and the production of energy (fig. 6*A*). This feature could be related to the fact that no other obligate endosymbiont partner was detected in the host (*X. cascus*) bacteriome or surrounding tissues, and all the amino acid biosynthetic roles rely on *Evansia* (Kuechler et al. 2013). Because *Evansia*, *Portiera,* and *Carsonella* seem to hold a subset of the gene repertoire of their free-living relatives (supplementary fig. S5, Supplementary Material online), evolution of their respective gene repertories could be related to the diet of each insect. Thus, psyllids could have access to a more enriched diet than other sap-sucking insects, and this could explain that some *Carsonella* strains have lost some amino acid biosynthetic pathways without an endosymbiotic partner. In contrast, whiteflies feed on phloem and usually harbor one or more secondary symbionts that share the same bacteriocyte with *Portiera* and probably complement the lost biosynthetic pathways (Gottlieb et al. 2008; Santos-Garcia et al. 2012; Sloan and Moran 2012a). In this context, *X. cascus* (and Peloridiidae in general) feeds on bryophytes (mosses and liverworts) that appear much poorer in nitrogen and sulfur compounds when compared with tracheophytes (higher plants) (Huneck 1983; Klavina et al. 2012). The analysis of *Evansia* metabolism showed that it has a large ability to synthetize most of proteinogenic amino acids, including ten essentials, six nonessentials, and N-formyl-MET (figs. 6 and 7). This seems to be related with the poor nitrogen content found in bryophytes (Klavina et al*.* 2012). There are also other gene sets whose retention in the small *Evansia* genome seems to be associated with the maintenance of any metabolic piece required for the synthesis of amino acids. For example, the pentose phosphate pathway, TCA cycle, and respiratory machinery are connected with the requirement of this huge amino acid biosynthetic machinery. The retention of the cobalamin biosynthetic pathway was also associated with the synthesis of amino acids, because this cofactor is required for the last step of MET synthesis. In contrast to *Carsonella* and *Portiera* and other primary endosymbionts, *Evansia* has maintained the MetH enzyme (cobalamin dependent) in opposition to MetE (cobalamin independent). The cobalamin biosynthetic pathway in *Evansia* lacks a nitroreductase that is present in *H. elongata* (and other Halomonadaceae) but not in several *Pseudomonas* (both able to synthesize cobalamin), suggesting that another enzyme can supply this step in *Evansia*. The retention of the ability to synthesize 5-methyltetrahydrofolate is also associated to the synthesis of MET (fig. 6*A*). The retention of gene *rthA*, which encodes a transporter able to export THR outside the bacterial cell, seems to be associated with the insect-endosymbiont shared biosynthesis of ILE. The interrupted pathway from THR to ILE may be bypassed in a similar way at the one observed in the aphid*-Bu. aphidicola* system (Wilson et al*.* 2010) by exporting THR to the insect cell, which will convert it in 2-oxobutanoate, compound that would enter the bacterium to continue the ILE biosynthetic pathway. However, because this enzyme is absent in the family Halomonadaceae, and a D-alpha,beta-D-heptose 1,7-bisphosphate phosphatase has been proposed to fill this gap by the gapfiller tool from Ecocyc. Nevertheless, it is possible that another phosphatase (i.e., *serB*) could do the function as well. Finally, in contrast to *Bu. aphidicola,* the gene encoding the last step (*ilvE*) is present in *Evansia*.

### Sulfur Metabolism in *Evansia*

Sulfur metabolism is another intriguing feature in *Evansia* when it is compared with other endosymbionts. Most of the extreme reduced endosymbionts usually retain the *sufBCDES* operon, which is required for the formation of the Fe-S proteins. This system has been proposed as an indicative of an oxidative stress environment in endosymbionts because it is more resistant to ROS than the *isc* operon (Lee et al. 2004; Lill 2009; Py and Barras 2010) but seems more an adaptation of the first stages of pathogenesis/endosymbiosis (Toft and Andersson 2010). In these stages, the host's immune system are fighting against the symbiont/pathogen, and one known reaction is to increase the oxidative stress, and it is reasonable that pathogenic/endosymbiotic bacteria have retained a Fe-S machinery that can work under these conditions (Huet et al*.* 2005). In addition, *Evansia* has retained two related A-type carriers, IscA and ErpA (Lill 2009; Py and Barras 2010). These proteins have been proposed as alternative routes for Fe-S assembly or as scaffolder proteins that carry the formed Fe-S cluster to the target apoprotein (Lill 2009; Py and Barras 2010). It also contains a glutaredoxin 4 homolog (*grxD*) that has been recently involved in an alternative Fe-S pathway (Li and Outten 2012) and YggX protein that is involved in Fe-S cluster repair after ROS damage. This suggests an oxidative environment potentially related to some toxins present in bryophytes (revised in Asakawa et al*.* 2013) although it could also be related to the endogenous detox pathway because *Evansia* seems to have a very active metabolism. It is also possible that the low disposition of sulfur in bryophytes is the clue for the maintenance of this enriched sulfur machinery, including the recycling and protection of the Fe-S clusters.

Surprisingly, an SQR, which allows the oxidations of H_2_S to sulfur or polysulfide chains, is present in *Evansia* (Marcia et al*.* 2010) (fig. 6*A*–*C*)*.* This enzyme has been described in phototrophic sulfur bacteria and in symbionts from marine vent hosts and armored snails, but the sulfur oxidation machinery is much more complex in these organisms than in *Evansia* (Frigaard and Dahl 2009; Harada et al*.* 2009; Nakagawa et al*.* 2014). Likewise, SQR may be part of a mitochondrial detoxification machinery against H_2_S in combination with a rhodanese (RHOD) and a sulfur dioxygenase (SDO) (Hildebrandt and Grieshaber 2008). This system also seems to be a common mechanism for sulfur oxidation in a wide range of heterotrophic bacteria (Liu et al*.* 2014). Because of the low amount of inorganic sulfur (usually uptaken from the soil in the form of sulfate or sulfite) in bryophytes, it is possible that the only source of organic sulfur for the system *Xenophyes–Evansia* comes from *Evansia.* Oxidized sulfur, present in the insect diet, could be reduced by the insect to H_2_S. This reduced form is used by *Evansia* to produce MET that could be exported to the host's cytosol where it would be used for the synthesis of CYS, one of the organic forms of sulfur. If some H_2_S could not be used for MET biosynthesis, SQR could be involved in the storage of sulfur (S) for its use when required. Intracellular sulfur (S) is then oxidized spontaneously to thiosulfate. At this point, two hypotheses arise: 1) SQR and YceA proteins are part of a recycling mechanism used to ensure maximum usage of H_2_S or 2) a combination of SQR, YceA, and GrxD transfers the leftover H_2_S to the host's cytosol as a detoxifying mechanism (fig. 6*B* and *C*).

If the SQR is working in a recycling manner, it is possible that the YceA protein (as other rhodaneses) could regenerate one H_2_S and to produce a sulfite molecule to be exported to the host for excretion because no sulfite oxidase has been detected in *Evansia* (Cheng et al*.* 2008) (fig. 6*B*). Furthermore, and taking into account that sulfur is a limiting factor, it is possible that a sulfite oxidase (present in most of the sequenced arthropods) could use the sulfite to produce other intermediate metabolites as a way of *Evansia*'s “waste recycling” like nitrogen metabolism in *Blattabacterium cuenotti* (López-Sánchez et al*.* 2009; González-Domenech et al*.* 2012) (fig. 6*B*). On the other hand, H_2_S is a toxic compound (similar to cyanide) that, even at low concentrations, can interfere with the electron transport chain. In this context, and in combination with the rhodanese YceA and the glutaredoxin 4 GrxD, SQR could be involved in the elimination of the remaining hydrogen sulphide, which is not used in the MET biosynthesis (fig. 6*C*). In this case, SQR would oxidize H_2_S to thiosulfate that is combined with glutathione (GSH) by YceA to form glutathione sulfide (GSSH). GSSH could be transferred to the host's cytosol by GrxD where SDO would oxidize it, releasing a glutathione molecule and a sulfite. Finally, sulfite could be excreted or used in the host metabolism (PAPS biosynthesis). Because both pathways share most of the enzymes, it is possible that both are co-occurring in *Evansia*–*Xenophyes* system to ensure the optimization of sulfur utilization.

## Conclusion

In summary, in spite of the long-term endosymbiotic life associated with moss bugs, *Evansia* has retained almost any gene required for the biosynthesis of amino acids including those involved in the synthesis of the enzyme cofactors, sulfur metabolism, and central metabolism. No special gene for detoxification of biologically active compounds (e.g., sesquiterpenoids) could be found in *Evansia*, so far. This indicates that moss bugs possibly do not come into contact with any of these toxic secondary metabolites by sucking only on the conductive tissue (that should contain only a minor amount of antifeedant) or that possible toxic metabolites will be degraded by gut microbiota or the host detoxification system (Hansen and Moran 2014), for example, via the extremely huge Malpighian tubules in moss bugs. Further studies about the insect (symbiont)–plant interaction will bring more light into this fascinating system.

## Supplementary Material

Supplementary tables S1 and S2, figures S1–S5 (Supplementary file 1), and Supplementary file 2 are available at *Genome Biology and Evolution* online (http://www.gbe.oxfordjournals.org/).

Supplementary Data

## References

[evu149-B1] Alaminos M, Ramos J (2001). The methionine biosynthetic pathway from homoserine in *Pseudomonas putida* involves the *metW*, *metX*, *metZ*, *metH* and *metE* gene products. Arch Microbiol..

[evu149-B2] Allen JM, Light JE, Perotti MA, Braig HR, Reed DL (2009). Mutational meltdown in primary endosymbionts: selection limits Muller’s ratchet. PLoS One.

[evu149-B3] Ardell DH, Andersson SGE (2006). TFAM detects co-evolution of tRNA identity rules with lateral transfer of histidyl-tRNA synthetase. Nucleic Acids Res..

[evu149-B4] Asakawa Y, Ludwiczuk A, Nagashima F (2013). Phytochemical and biological studies of bryophytes. Phytochemistry.

[evu149-B5] Aziz RK (2008). The RAST server: rapid annotations using subsystems technology. BMC Genomics.

[evu149-B6] Baumann P (2005). Biology bacteriocyte-associated endosymbionts of plant sap-sucking insects. Annu Rev Microbiol..

[evu149-B7] Belda E, Moya A, Silva FJ (2005). Genome rearrangement distances and gene order phylogeny in gamma-Proteobacteria. Mol Biol Evol..

[evu149-B8] Bernt M (2013). MITOS: improved de novo metazoan mitochondrial genome annotation. Mol Phylogenet Evol..

[evu149-B9] Boetzer M, Henkel CV, Jansen HJ, Butler D, Pirovano W (2011). Scaffolding pre-assembled contigs using SSPACE. Bioinformatics.

[evu149-B10] Boetzer M, Pirovano W (2012). Toward almost closed genomes with GapFiller. Genome Biol..

[evu149-B11] Bourque G, Pevzner PA (2002). Genome-scale evolution: reconstructing gene orders in the ancestral species. Genome Res..

[evu149-B12] Brady A, Salzberg S (2011). PhymmBL expanded: confidence scores, custom databases, parallelization and more. Nat Methods..

[evu149-B13] Buchner P (1965). Endosymbiosis of animals with plant microorganisms.

[evu149-B14] Burckhardt D (2009). Taxonomy and phylogeny of the Gondwanan moss bugs or Peloridiidae (Hemiptera, Coleorrhyncha). Dtsch Entomol Z..

[evu149-B15] Burge SW (2013). Rfam 11.0: 10 years of RNA families. Nucleic Acids Res..

[evu149-B16] Burrows M, Hartung V, Hoch H (2007). Jumping behaviour in a Gondwanan relict insect (Hemiptera: Coleorrhyncha: Peloridiidae). J Exp Biol..

[evu149-B17] Castresana J (2000). Selection of conserved blocks from multiple alignments for their use in phylogenetic analysis. Mol Biol Evol..

[evu149-B18] Charron C, Roy H, Blaise M, Giegé R, Kern D (2003). Non-discriminating and discriminating aspartyl-tRNA synthetases differ in the anticodon-binding domain. EMBO J..

[evu149-B19] Cheng H, Donahue JL, Battle SE, Ray WK, Larson TJ (2008). Biochemical and genetic characterization of PspE and GlpE, two single-domain sulfurtransferases of *Escherichia coli*. Open Microbiol. J..

[evu149-B20] Chevreux B, Wetter T, Suhai S (1999). Genome sequence assembly using trace signals and additional sequence information. Comput Sci Biol Proc Ger Conf Bioinform..

[evu149-B21] Conord C (2008). Long-term evolutionary stability of bacterial endosymbiosis in curculionoidea: additional evidence of symbiont replacement in the dryophthoridae family. Mol Biol Evol..

[evu149-B22] Costa HS, Westcot DM, Ullman DE, Johnson MW (1993). Ultrastructure of the endosymbionts of the whitefly, *Bemisia tabaci* and *Trialeurodes vaporariorum*. Protoplasma.

[evu149-B23] Cryan JR, Urban JM (2012). Higher-level phylogeny of the insect order Hemiptera: is Auchenorrhyncha really paraphyletic?. Syst Entomol.

[evu149-B24] Darriba D, Taboada GL, Doallo R, Posada D (2011). ProtTest 3: fast selection of best-fit models of protein evolution. Bioinformatics.

[evu149-B25] Evans JW (1948). Some former inhabitants of Antarctica. The illustrated London News, February 21..

[evu149-B26] Foster B (2012). POLISHER: a tool for using ultra short reads in genome sequence improvement. http://jgi.doe.gov/data-and-tools/polisher/.

[evu149-B27] Frigaard NU, Dahl C (2009). Sulfur metabolism in phototrophic sulfur bacteria. Adv Microb Physiol..

[evu149-B28] Gil R, Silva FJ, Peretó J, Moya A (2004). Determination of the Core of a Minimal Bacterial Gene Set. Microbiol Mol Biol Rev..

[evu149-B29] González-Domenech CM (2012). Metabolic stasis in an ancient symbiosis: genome-scale metabolic networks from two *Blattabacterium cuenoti* strains, primary endosymbionts of cockroaches. BMC Microbiol..

[evu149-B30] Gottlieb Y (2008). Inherited intracellular ecosystem: symbiotic bacteria share bacteriocytes in whiteflies. FASEB J..

[evu149-B31] Gralnick J, Downs D (2001). Protection from superoxide damage associated with an increased level of the YggX protein in *Salmonella enterica*. Proc Natl Acad Sci U S A..

[evu149-B32] Hansen AK, Moran NA (2014). The impact of microbial symbionts on host plant utilization by herbivorous insects. Mol Ecol..

[evu149-B33] Harada M (2009). Expression of genes for sulfur oxidation in the intracellular chemoautotrophic symbiont of the deep-sea bivalve *Calyptogena okutanii*. Extremophiles.

[evu149-B34] Hernández-Montalvo V (2003). Expression of galP and glk in a *Escherichia coli* PTS mutant restores glucose transport and increases glycolytic flux to fermentation products. Biotechnol Bioeng..

[evu149-B35] Hildebrandt TM, Grieshaber MK (2008). Three enzymatic activities catalyze the oxidation of sulfide to thiosulfate in mammalian and invertebrate mitochondria. FEBS J..

[evu149-B36] Hoch H, Deckert J, Wessel A (2006). Vibrational signalling in a Gondwanan relict insect (Hemiptera: Coleorrhyncha: Peloridiidae). Biol Lett..

[evu149-B37] Huet G, Daffé M, Saves I (2005). Identification of the *Mycobacterium tuberculosis* SUF machinery as the exclusive mycobacterial system of [Fe-S] cluster assembly: evidence for its implication in the pathogen’s survival. J Bacteriol..

[evu149-B38] Huneck S, Schuster RM (1983). Chemistry and biochemistry of bryophytes. New manual of bryology.

[evu149-B39] Husnik F (2013). Horizontal gene transfer from diverse bacteria to an insect genome enables a tripartite nested mealybug symbiosis. Cell.

[evu149-B40] Karp PD, Paley S, Romero P (2002). The pathway tools software. Bioinformatics.

[evu149-B41] Karp PD (2005). Expansion of the BioCyc collection of pathway/genome databases to 160 genomes. Nucleic Acids Res..

[evu149-B42] Katoh K, Misawa K, Kuma K, Miyata T (2002). MAFFT: a novel method for rapid multiple sequence alignment based on fast Fourier transform. Nucleic Acids Res..

[evu149-B43] Kelkar YD, Ochman H (2013). Genome reduction promotes increase in protein functional complexity in bacteria. Genetics.

[evu149-B44] Klavina L, Bikovens O, Steinberga I, Maksimova V, Eglite L (2012). Characterization of chemical composition of some bryophytes common in Latvia. Environ Exp Biol..

[evu149-B45] Koga R, Bennett GM, Cryan JR, Moran NA (2013). Evolutionary replacement of obligate symbionts in an ancient and diverse insect lineage. Environ Microbiol..

[evu149-B46] Kuechler SM, Gibbs G, Burckhardt D, Dettner K, Hartung V (2013). Diversity of bacterial endosymbionts and bacteria-host co-evolution in Gondwanan relict moss bugs (Hemiptera: Coleorrhyncha: Peloridiidae). Environ Microbiol..

[evu149-B47] Krzywinski M (2009). Circos: an information aesthetic for comparative genomics. Genome Res..

[evu149-B48] Lamelas A (2011). *Serratia symbiotica* from the aphid *Cinara cedri*: a missing link from facultative to obligate insect endosymbiont. PLoS Genet..

[evu149-B49] Lartillot N, Lepage T, Blanquart S (2009). PhyloBayes 3: a Bayesian software package for phylogenetic reconstruction and molecular dating. Bioinformatics.

[evu149-B50] Lee JH, Yeo WS, Roe JH (2004). Induction of the sufA operon encoding Fe-S assembly proteins by superoxide generators and hydrogen peroxide: involvement of OxyR, IHF and an unidentified oxidant-responsive factor. Mol Microbiol..

[evu149-B51] Li H, Outten CE (2012). Monothiol CGFS glutaredoxins and BolA-like proteins: [2Fe-2S] binding partners in iron homeostasis. Biochemistry.

[evu149-B52] Li L, Stoeckert CJ, Roos DS (2003). OrthoMCL: identification of ortholog groups for eukaryotic genomes. Genome Res..

[evu149-B53] Lill R (2009). Function and biogenesis of iron-sulphur proteins. Nature.

[evu149-B54] Lin Y, Rajan V, Moret BM (2012). TIBA: a tool for phylogeny inference from rearrangement data with bootstrap analysis. Bioinformatics.

[evu149-B55] Liu H, Xin Y, Xun L (2014). Distribution, diversity, and activities of sulfur dioxygenases in heterotrophic bacteria. Appl Environ Microbiol..

[evu149-B56] Livshits VA, Zakataeva NP, Aleshin VV, Vitushkina MV (2003). Identification and characterization of the new gene *rhtA* involved in threonine and homoserine efflux in *Escherichia coli*. Res Microbiol..

[evu149-B57] López-Sánchez MJ (2009). Evolutionary convergence and nitrogen metabolism in *Blattabacterium* strain Bge, primary endosymbiont of the cockroach *Blattella germanica*. PLoS Genet..

[evu149-B58] Manzano-Marín A, Lamelas A, Moya A, Latorre A (2012). Comparative genomics of *Serratia spp*.: two paths towards endosymbiotic life. PLoS One.

[evu149-B59] Marcia M, Ermler U, Peng G, Michel H (2010). A new structure-based classification of sulfide:quinone oxidoreductases. Proteins.

[evu149-B60] Markowitz VM (2014). IMG 4 version of the integrated microbial genomes comparative analysis system. Nucleic Acids Res..

[evu149-B61] Martin VJ, Mohn WW (2000). Genetic investigation of the catabolic pathway for degradation of abietane diterpenoids by *Pseudomonas abietaniphila* BKME-9. J Bacteriol..

[evu149-B62] McCutcheon JP, McDonald BR, Moran NA (2009). Convergent evolution of metabolic roles in bacterial co-symbionts of insects. Proc Natl Acad Sci U S A..

[evu149-B63] McCutcheon JP, Moran NA (2012). Extreme genome reduction in symbiotic bacteria. Nat Rev Microbiol..

[evu149-B64] Mera PE, Escalante-Semerena JC (2010). Dihydroflavin-driven adenosylation of 4-coordinate Co(II) corrinoids: are cobalamin reductases enzymes or electron transfer proteins?. J Biol Chem..

[evu149-B65] Moran NA (1996). Accelerated evolution and Muller’s rachet in endosymbiotic bacteria. Proc Natl Acad Sci U S A..

[evu149-B66] Moya A, Peretó J, Gil R, Latorre A (2008). Learning how to live together: genomic insights into prokaryote-animal symbioses. Nat Rev Genet..

[evu149-B67] Müller HJ (1951). Über die intrazellulare symbiose der peloridiidae *Hemiodoecus fidelis* evans und ihre stellung unter den homopterensymbiosen. Zool Anz..

[evu149-B68] Nakagawa S (2014). Allying with armored snails: the complete genome of gammaproteobacterial endosymbiont. ISME J..

[evu149-B69] Nowack ECM, Grossman AR (2012). Trafficking of protein into the recently established photosynthetic organelles of *Paulinella chromatophora*. Proc Natl Acad Sci U S A..

[evu149-B70] Oakeson KF (2014). Genome degeneration and adaptation in a nascent stage of symbiosis. Genome Biol Evol..

[evu149-B71] Parsonage D, Karplus PA, Poole LB (2008). Substrate specificity and redox potential of AhpC, a bacterial peroxiredoxin. Proc Natl Acad Sci U S A..

[evu149-B72] Pendergrast JG (1962). The internal anatomy of the Peloridiidae. Ecol Entomol..

[evu149-B73] Popov YA, Shcherbakov DE, Schaefer CW (1996). Origin and evolution of the Coleorrhyncha as shown by the fossil record. Studies on Hemipteran phylogeny.

[evu149-B74] Py B, Barras F (2010). Building Fe-S proteins: bacterial strategies. Nat Rev Microbiol..

[evu149-B75] Quevillon E (2005). InterProScan: protein domains identifier. Nucleic Acids Res..

[evu149-B76] R Development Core Team (2014). R: a language and environment for statistical computing [Internet]. http://www.r-project.org/.

[evu149-B77] Reyes-Prieto M, Latorre A, Moya A (2013). Scanty microbes, the “symbionelle” concept. Environ Microbiol..

[evu149-B78] Santos-Garcia D (2012). Complete genome sequence of “*Candidatus* Portiera aleyrodidarum” BT-QVLC, an obligate symbiont that supplies amino acids and carotenoids to *Bemisia tabaci*. J Bacteriol..

[evu149-B79] Schomburg I (2013). BRENDA in 2013: integrated reactions, kinetic data, enzyme function data, improved disease classification: new options and contents in BRENDA. Nucleic Acids Res..

[evu149-B80] Segata N, Börnigen D, Morgan XC, Huttenhower C (2013). PhyloPhlAn is a new method for improved phylogenetic and taxonomic placement of microbes. Nat Commun..

[evu149-B81] Shcherbakov D (2000). The most primitive whiteflies (Hemiptera; Aleyrodidae; Bernaeinae subfam. nov.) from the Mesozoic of Asia and Burmese amber, with an overview of Burmese amber hemipterans. Bull Nat Hist Mus Lond (Geol.)..

[evu149-B82] Silva FJ, Latorre A, Moya A (2001). Genome size reduction through multiple events of gene disintegration in *Buchnera* APS. Trends Genet..

[evu149-B83] Silva FJ, Latorre A, Moya A (2003). Why are the genomes of endosymbiotic bacteria so stable?. Trends Genet..

[evu149-B84] Sloan DB, Moran NA (2012a). Endosymbiotic bacteria as a source of carotenoids in whiteflies. Biol Lett..

[evu149-B85] Sloan DB, Moran NA (2012b). Genome reduction and co-evolution between the primary and secondary bacterial symbionts of psyllids. Mol Biol Evol..

[evu149-B86] Sloan DB, Moran NA (2013). The evolution of genomic instability in the obligate endosymbionts of whiteflies. Genome Biol Evol..

[evu149-B87] Sloan DB (2014). Parallel Histories of horizontal gene transfer facilitated extreme reduction of endosymbiont genomes in sap-feeding insects. Mol Biol Evol..

[evu149-B88] Song N, Liang AP, Bu CP (2012). A molecular phylogeny of Hemiptera inferred from mitochondrial genome sequences. PLoS One.

[evu149-B89] Spangenberg R (2013). The cephalic morphology of the Gondwanan key taxon Hackeriella (Coleorrhyncha, Hemiptera). Arthropod Struct Dev..

[evu149-B90] Staden R, Beal KF, Bonfield JK (2000). The Staden package, 1998. Methods Mol Biol..

[evu149-B91] Stamatakis A (2006). RAxML-VI-HPC: maximum likelihood-based phylogenetic analyses with thousands of taxa and mixed models. Bioinformatics.

[evu149-B92] Tamames J (2007). The frontier between cell and organelle: genome analysis of *Candidatus* Carsonella ruddii. BMC Evol Biol..

[evu149-B93] Tamas I (2002). 50 Million years of genomic stasis in endosymbiotic bacteria. Science.

[evu149-B94] Tatusov RL, Galperin MY, Natale DA, Koonin EV (2000). The COG database: a tool for genome-scale analysis of protein functions and evolution. Nucleic Acids Res..

[evu149-B95] Thao MLL, Baumann P (2004). Evolutionary relationships of primary prokaryotic endosymbionts of whiteflies and their hosts. Appl Environ Microbiol..

[evu149-B96] Toft C, Andersson SGE (2010). Evolutionary microbial genomics: insights into bacterial host adaptation. Nat Rev Genet..

[evu149-B97] Vyazmensky M, Sella C, Barak Z, Chipman DM (1996). Isolation and characterization of subunits of acetohydroxy acid synthase isozyme III and reconstitution of the holoenzyme. Biochemistry.

[evu149-B98] Warnes GR (2013). gplots: various R programming tools for plotting data. http://cran.r-project.org/package=gplots.

[evu149-B99] White JA (2011). Caught in the act: rapid, symbiont-driven evolution. BioEssays.

[evu149-B100] Wilson ACC (2010). Genomic insight into the amino acid relations of the pea aphid, *Acyrthosiphon pisum*, with its symbiotic bacterium *Buchnera aphidicola*. Insect Mol Biol..

