# Supplementary material for: Small but Powerful, the Primary Endosymbiont of Moss Bugs, Candidatus Evansia muelleri, Holds a Reduced Genome with Large Biosynthetic Capabilities
Source: Genome Biol Evol. 2014 Jul 10;6(7):1875–93. doi: 10.1093/gbe/evu149 (PMC4122945; doi:10.1093/gbe/evu149)
Supplement: Supplementary Data [file supp_6_7_1875__index.html]

Small but Powerful, the Primary Endosymbiont of Moss Bugs, Candidatus Evansia muelleri, Holds a Reduced Genome with Large Biosynthetic Capabilities — Supplementary Data 

# Small but Powerful, the Primary Endosymbiont of Moss Bugs, *Candidatus* Evansia muelleri, Holds a Reduced Genome with Large Biosynthetic Capabilities

## Supplementary Data

files

**Files in this Data Supplement:**

- Supplementary Data - pdf file
- Supplementary Data - pdf file
- Supplementary Data - xls file
- Supplementary Data - xls file
