# Supplementary material for: Small but Powerful, the Primary Endosymbiont of Moss Bugs, Candidatus Evansia muelleri, Holds a Reduced Genome with Large Biosynthetic Capabilities
Source: Genome Biol Evol. 2014 Jul 10;6(7):1875–93. doi: 10.1093/gbe/evu149 (PMC4122945; doi:10.1093/gbe/evu149)
Supplement: Supplementary Data [file supp_evu149_Supplementary_file_1.pdf]

Figure S1

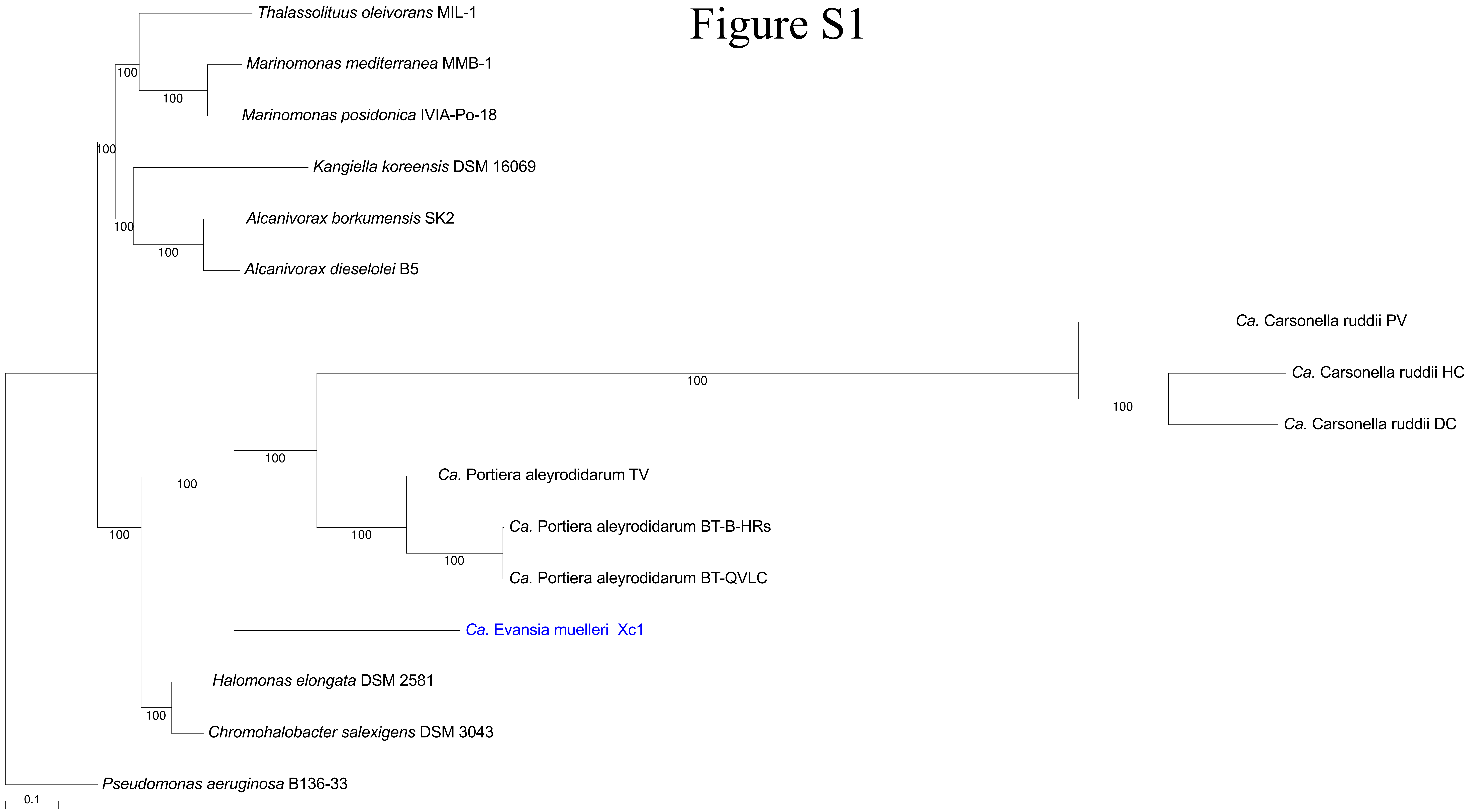

Thalassolituus oleivorans MIL-1
Marinomonas mediterranea MMB-1
Marinomonas posidonica IVIA-Po-18
Kangiella koreensis DSM 16069
Alcanivorax borkumensis SK2
Alcanivorax dieselolei B5
Ca. Carsonella ruddii PV
Ca. Carsonella ruddii HC
Ca. Carsonella ruddii DC
Ca. Portiera aleyrodidarum TV
Ca. Portiera aleyrodidarum BT-B-HRs
Ca. Portiera aleyrodidarum BT-QVLC
Ca. Evansia muelleri Xc1
Halomonas elongata DSM 2581
Chromohalobacter salexigens DSM 3043
Pseudomonas aeruginosa B136-33
100
0.1

Figure S2

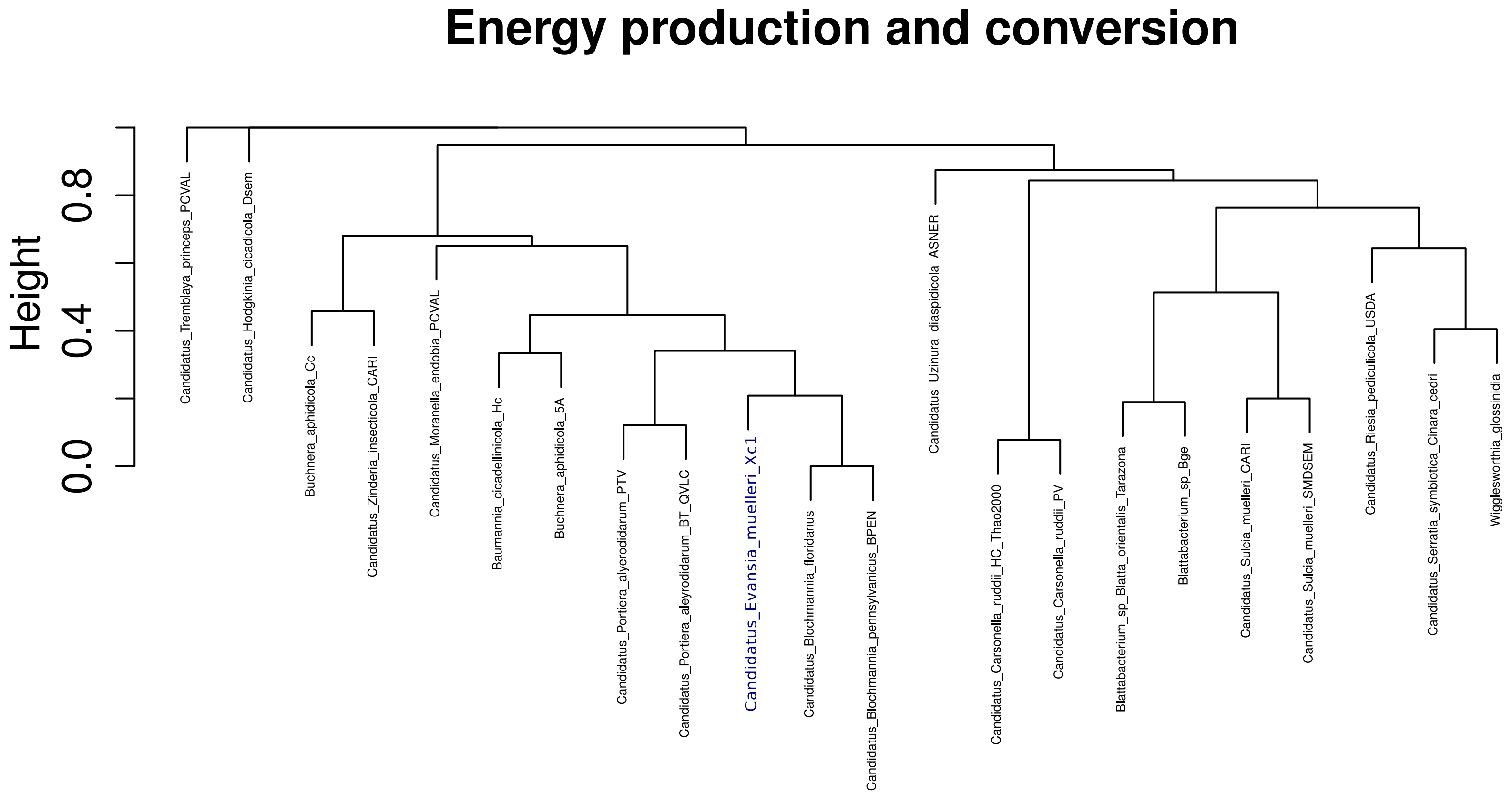
Energy production and conversion
Height
0.0
0.4
0.8
Candidatus_Tremblaya_princeps_PCVAL
Candidatus_Hodgkinia_cicadicola_Dsem
Buchnera_aphidicola_Cc
Candidatus_Zinderia_insecticola_CARI
Candidatus_Moranella_endobia_PCVAL
Baumannia_cicadellinicola_Hc
Buchnera_aphidicola_5A
Candidatus_Portiera_alyerodidarum_PTV
Candidatus_Portiera_aleyrodidarum_BT_QVLC
Candidatus_Evansia_muelleri_Xc1
Candidatus_Blochmannia_floridanus
Candidatus_Blochmannia_pennsylvanicus_BPEN
Candidatus_Uzinura_diaspidicola_ASNER
Candidatus_Carsonella_ruddii_HC_Thao2000
Candidatus_Carsonella_ruddii_PV
Blattabacterium_sp_Blatta_orientalis_Tarazona
Blattabacterium_sp_Bge
Candidatus_Sulcia_muelleri_CARI
Candidatus_Sulcia_muelleri_SMDSEM
Candidatus_Riesia_pediculicola_USDA
Candidatus_Serratia_symbiotica_Cinara_cedri
Wigglesworthia_glossinidia

Figure S3

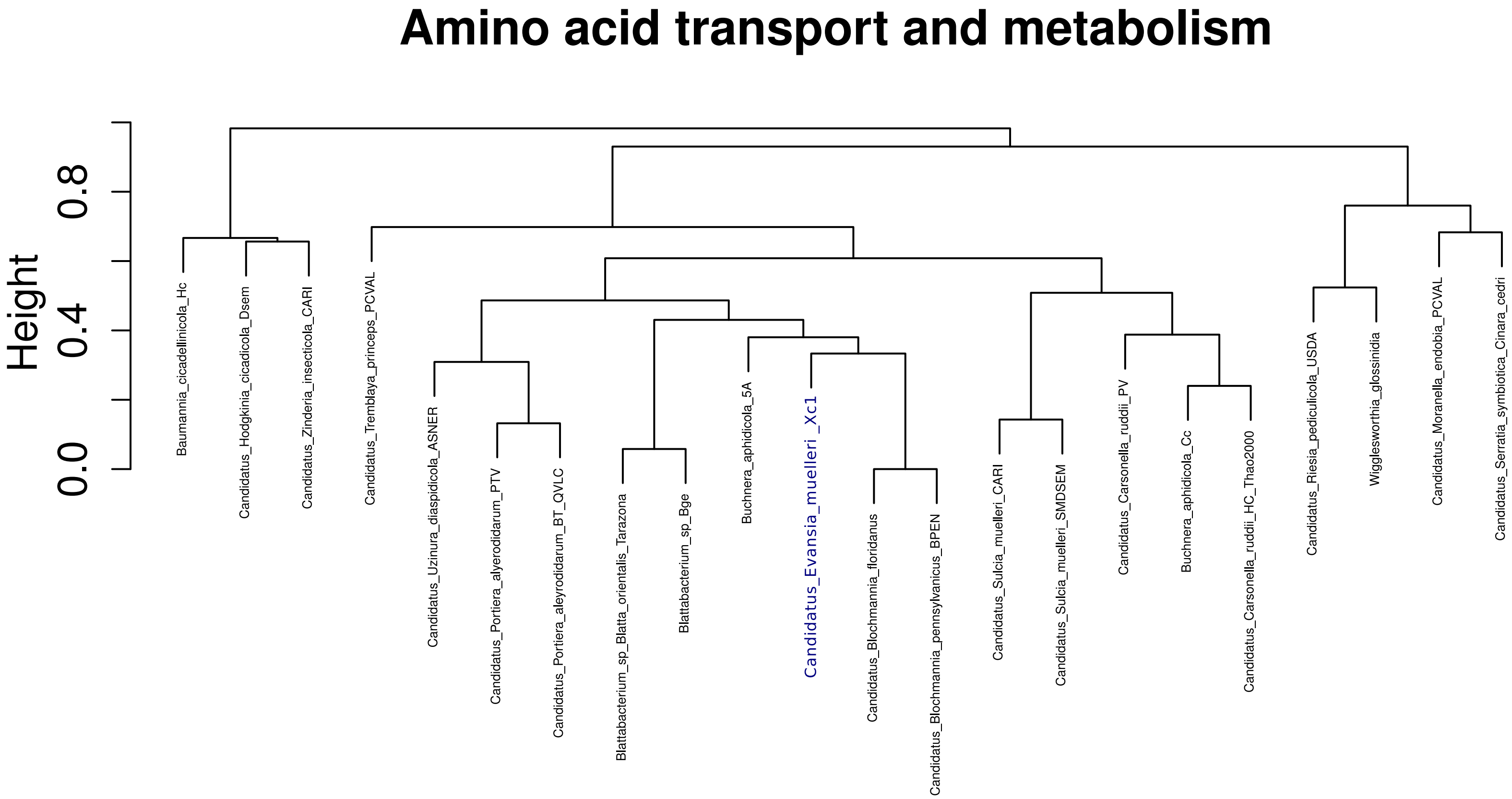

Amino acid transport and metabolism
Height
0.0
0.4
0.8
Baumannia_cicadellinicola_Hc
Candidatus_Hodgkinia_cicadicola_Dsem
Candidatus_Zinderia_insecticola_CARI
Candidatus_Tremblaya_princeps_PCVAL
Candidatus_Uzinura_diaspidicola_ASNER
Candidatus_Portiera_alyerodidarum_PTV
Candidatus_Portiera_aleyrodidarum_BT_QVLC
Blattabacterium_sp_Blatta_orientalis_Tarazona
Blattabacterium_sp_Bge
Buchnera_aphidicola_5A
Candidatus_Evansia_muelleri_Xc1
Candidatus_Blochmannia_floridanus
Candidatus_Blochmannia_pennsylvanicus_BPEN
Candidatus_Sulcia_muelleri_CARI
Candidatus_Sulcia_muelleri_SMDSEM
Candidatus_Carsonella_ruddii_PV
Buchnera_aphidicola_Cc
Candidatus_Carsonella_ruddii_HC_Thao2000
Candidatus_Riesia_pediculicola_USDA
Wigglesworthia_glossinidia
Candidatus_Moranella_endobia_PCVAL
Candidatus_Serratia_symbiotica_Cinara_cedri

Figure S4

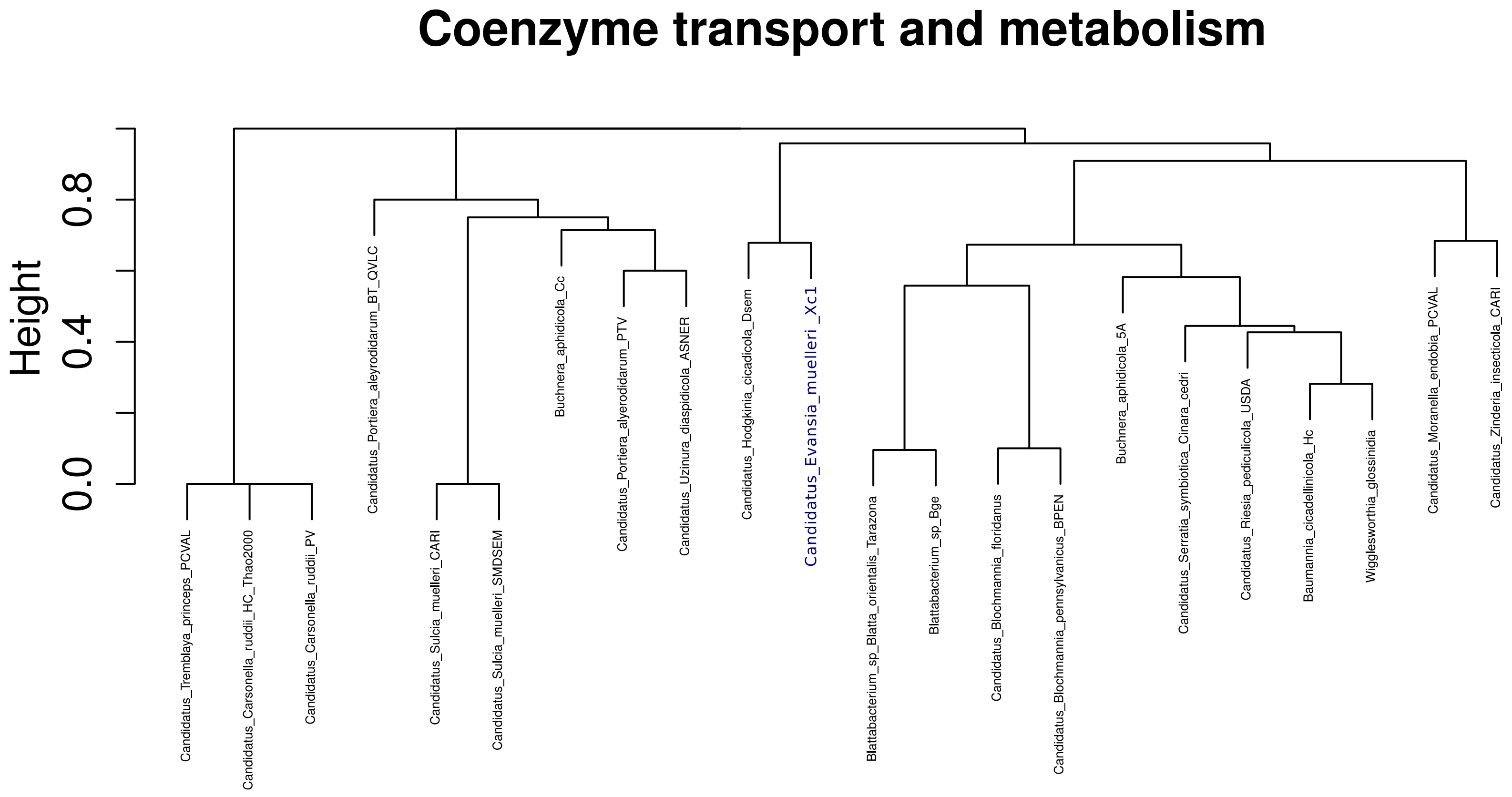
Coenzyme transport and metabolism
Height
0.0
0.4
0.8
Candidatus_Tremblaya_princeps_PCVAL
Candidatus_Carsonella_ruddii_HC_Thao2000
Candidatus_Carsonella_ruddii_PV
Candidatus_Portiera_aleyrodidarum_BT_QVLC
Candidatus_Sulcia_muelleri_CARI
Candidatus_Sulcia_muelleri_SMDSEM
Buchnera_aphidicola_Cc
Candidatus_Portiera_alyerodidarum_PTV
Candidatus_Uzinura_diaspidicola_ASNER
Candidatus_Hodgkinia_cicadicola_Dsem
Candidatus_Evansia_muelleri _Xc1
Blattabacterium_sp_Blatta_orientalis_Tarazona
Blattabacterium_sp_Bge
Candidatus_Blochmannia_floridanus
Candidatus_Blochmannia_pennsylvanicus_BPEN
Buchnera_aphidicola_5A
Candidatus_Serratia_symbiotica_Cinara_cedri
Candidatus_Riesia_pediculicola_USDA
Baumannia_cicadellinicola_Hc
Wigglesworthia_glossinidia
Candidatus_Moranella_endobia_PCVAL
Candidatus_Zinderia_insecticola_CARI

Figure S5

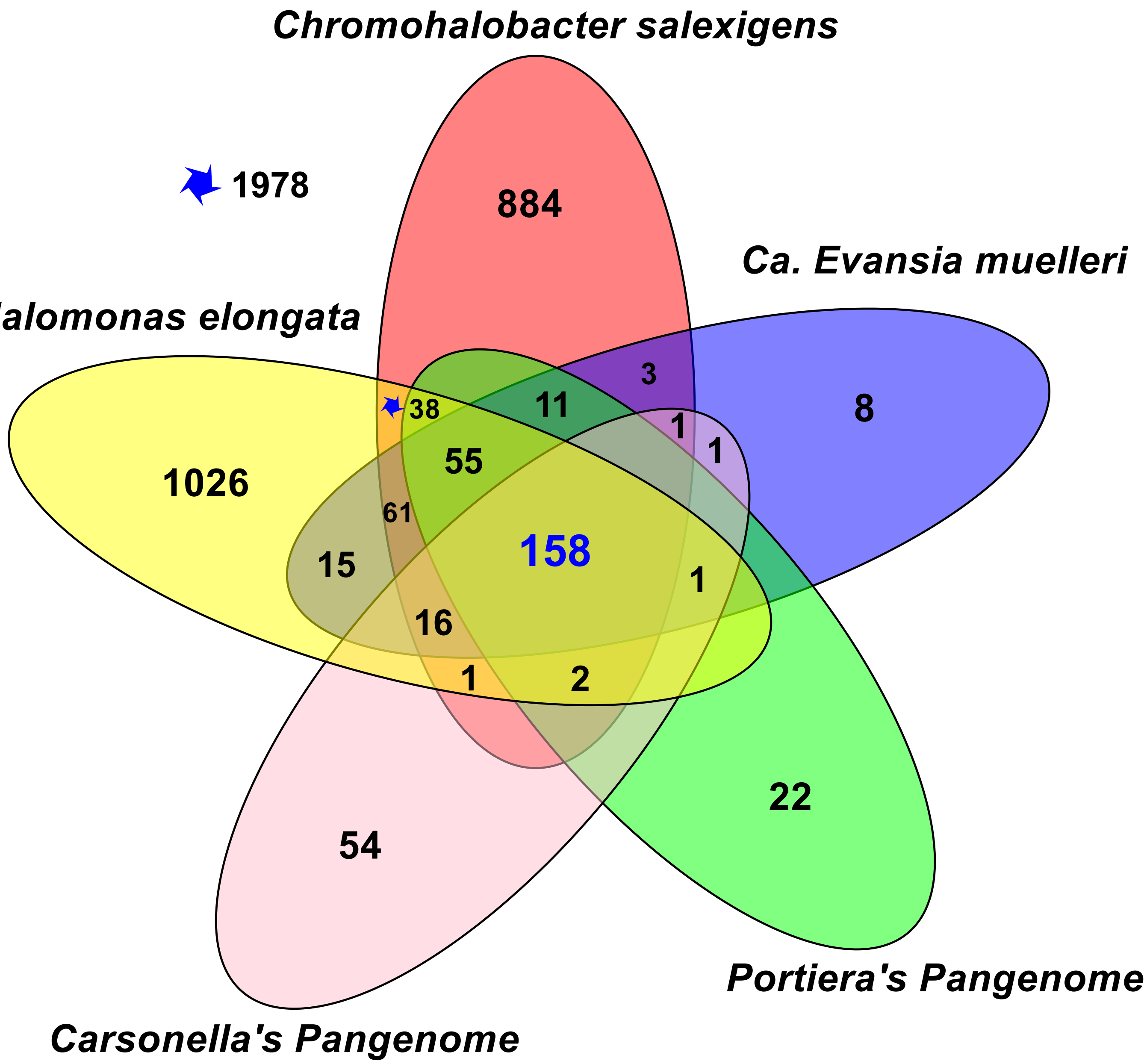
Chromohalobacter salexigens
Ca. Evansia muelleri
Halomonas elongata
Portiera's Pangenome
Carsonella's Pangenome
1978
884
8
1026
3
11
38
1
1
55
61
15
158
1
16
1
2
22
54
