# Supplementary material for: Small but Powerful, the Primary Endosymbiont of Moss Bugs, Candidatus Evansia muelleri, Holds a Reduced Genome with Large Biosynthetic Capabilities
Source: Genome Biol Evol. 2014 Jul 10;6(7):1875–93. doi: 10.1093/gbe/evu149 (PMC4122945; doi:10.1093/gbe/evu149)
Supplement: Supplementary Data [file supp_evu149_Supplementary_file_2.pdf]

ArgG

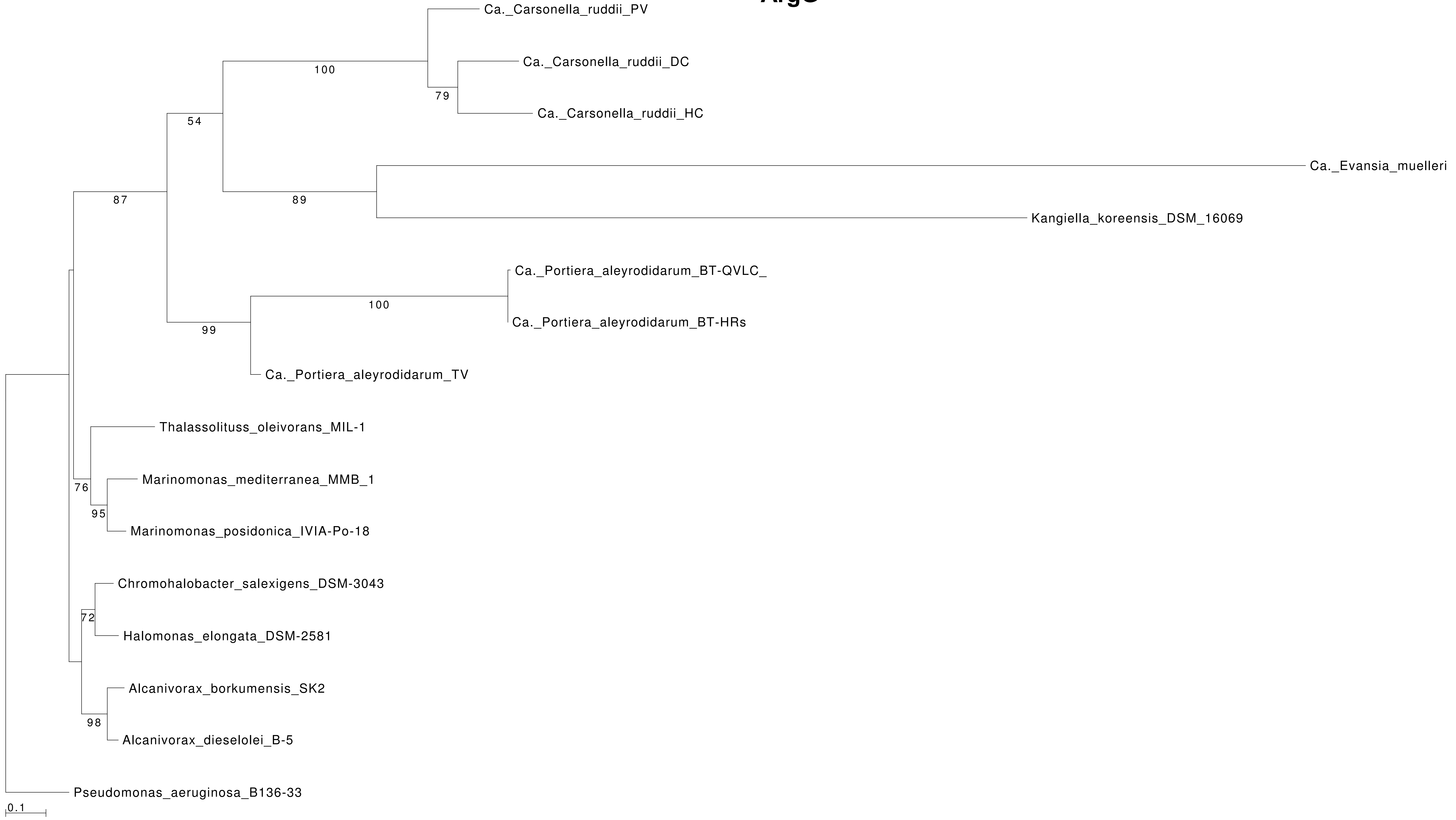

Ca._Carsonella_ruddii_PV
Ca._Carsonella_ruddii_DC
Ca._Carsonella_ruddii_HC
Ca._Evansia_muelleri
Kangiella_koreensis_DSM_16069
Ca._Portiera_aleyrodidarum_BT-QVLC_
Ca._Portiera_aleyrodidarum_BT-HRs
Ca._Portiera_aleyrodidarum_TV
Thalassolituss_oleivorans_MIL-1
Marinomonas_mediterranea_MMB_1
Marinomonas_posidonica_IVIA-Po-18
Chromohalobacter_salexigens_DSM-3043
Halomonas_elongata_DSM-2581
Alcanivorax_borkumensis_SK2
Alcanivorax_dieselolei_B-5
Pseudomonas_aeruginosa_B136-33
100
79
54
87
89
100
99
76
95
72
98
0.1

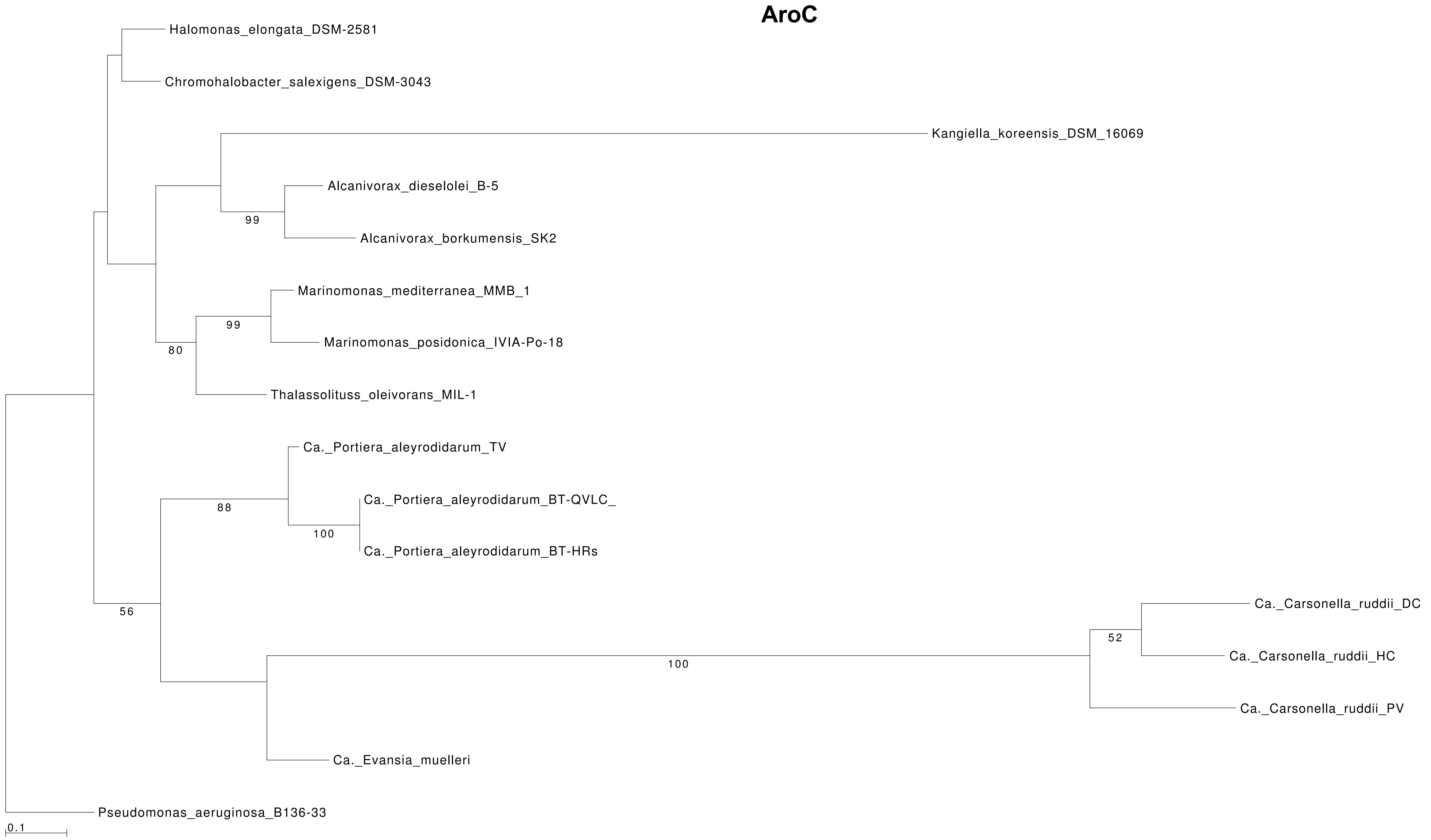
AroC
Halomonas_elongata_DSM-2581
Chromohalobacter_salexigens_DSM-3043
Kangiella_koreensis_DSM_16069
Alcanivorax_dieselolei_B-5
99
Alcanivorax_borkumensis_SK2
Marinomonas_mediterranea_MMB_1
99
Marinomonas_posidonica_IVIA-Po-18
80
Thalassolituss_oleivorans_MIL-1
Ca._Portiera_aleyrodidarum_TV
Ca._Portiera_aleyrodidarum_BT-QVLC_
88
100
Ca._Portiera_aleyrodidarum_BT-HRs
Ca._Carsonella_ruddii_DC
56
52
Ca._Carsonella_ruddii_HC
100
Ca._Carsonella_ruddii_PV
Ca._Evansia_muelleri
Pseudomonas_aeruginosa_B136-33
0.1

AtpA

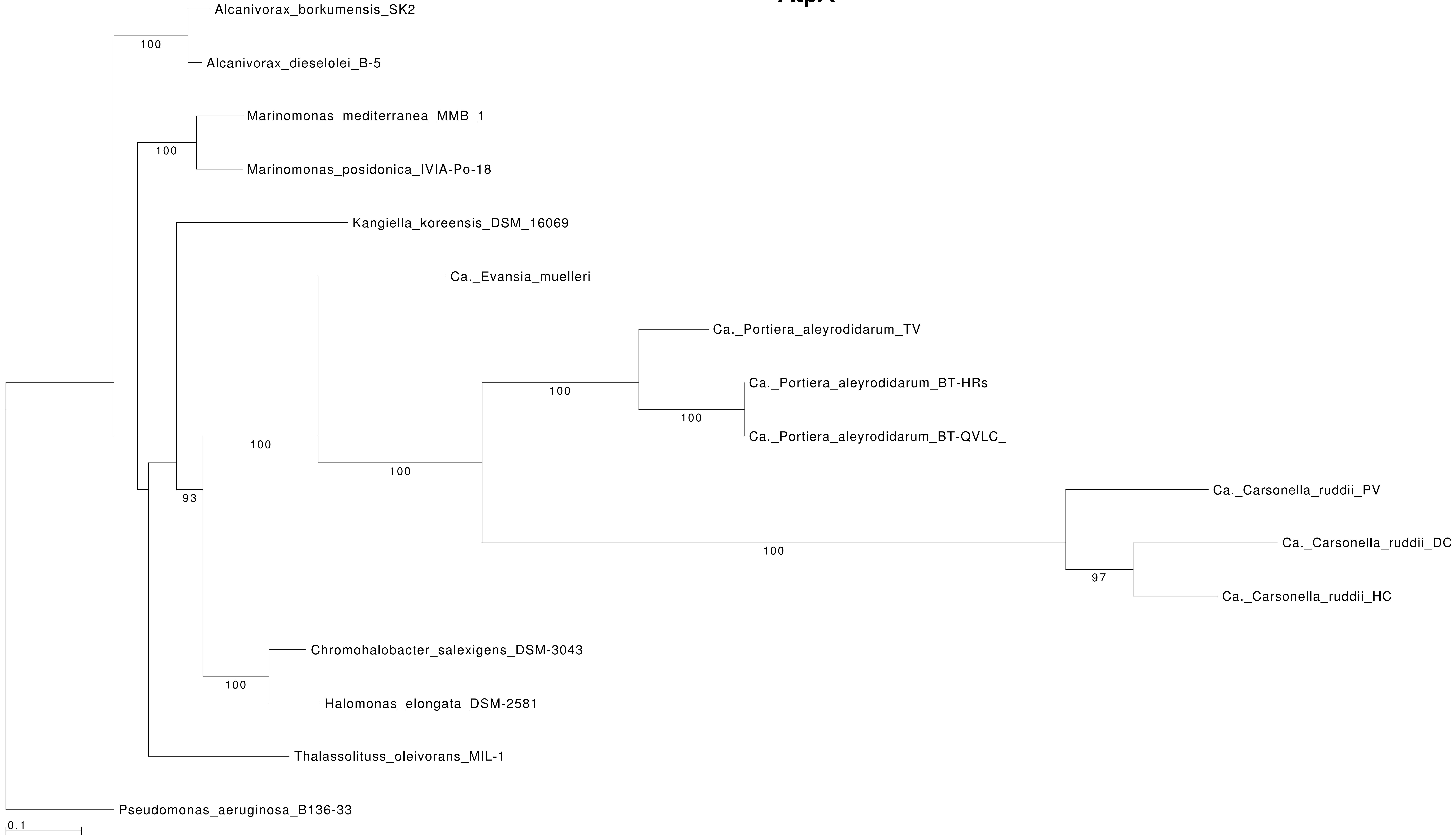
Alcanivorax_borkumensis_SK2
Alcanivorax_dieselolei_B-5
Marinomonas_mediterranea_MMB_1
Marinomonas_posidonica_IVIA-Po-18
Kangiella_koreensis_DSM_16069
Ca._Evansia_muelleri
Ca._Portiera_aleyrodidarum_TV
Ca._Portiera_aleyrodidarum_BT-HRs
Ca._Portiera_aleyrodidarum_BT-QVLC_
Ca._Carsonella_ruddii_PV
Ca._Carsonella_ruddii_DC
Ca._Carsonella_ruddii_HC
Chromohalobacter_salexigens_DSM-3043
Halomonas_elongata_DSM-2581
Thalassolituss_oleivorans_MIL-1
Pseudomonas_aeruginosa_B136-33
100
100
100
100
100
100
100
97
93
100
0.1

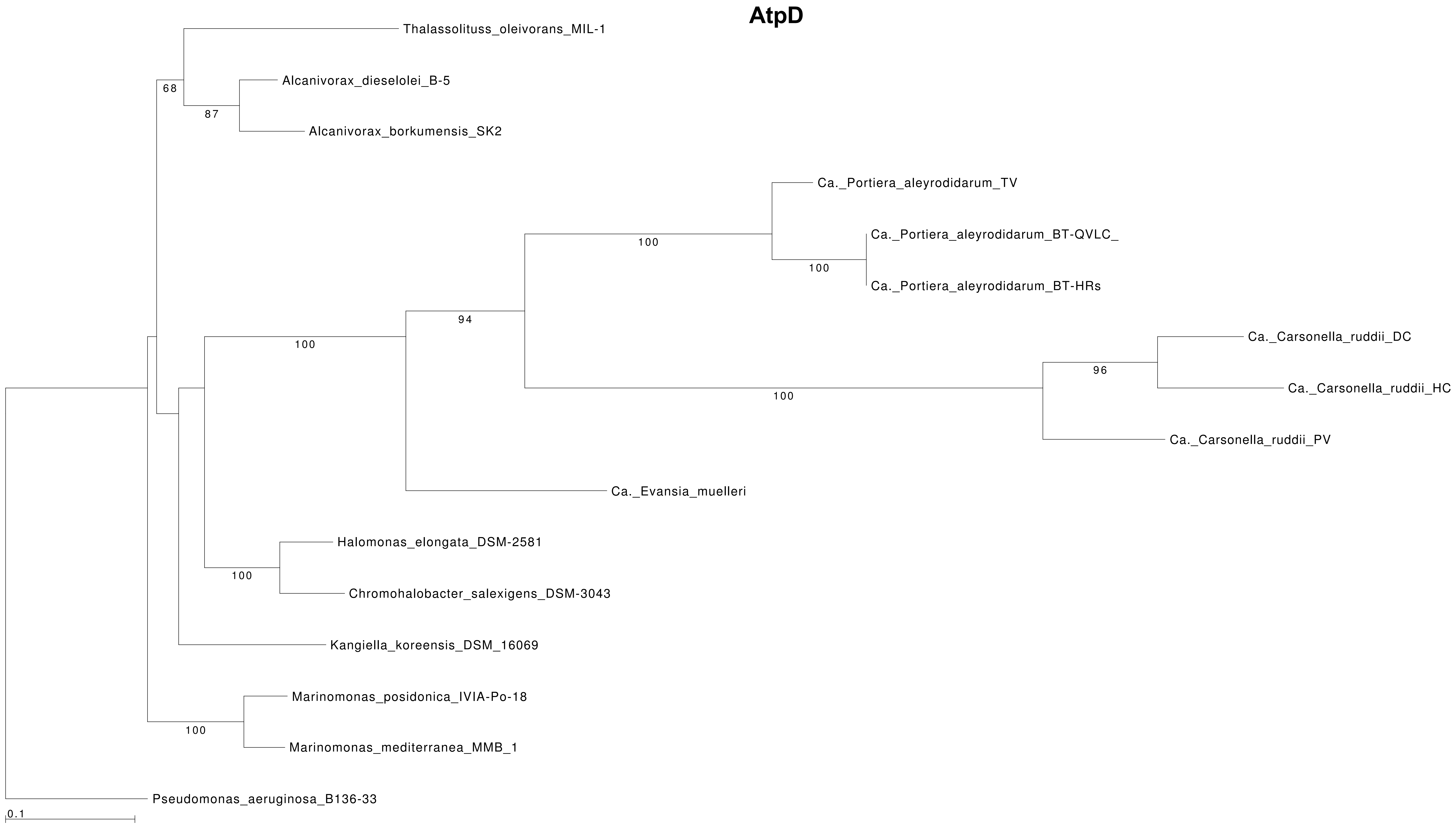

AtpD
Thalassolituss_oleivorans_MIL-1
Alcanivorax_dieselolei_B-5
Alcanivorax_borkumensis_SK2
Ca._Portiera_aleyrodidarum_TV
Ca._Portiera_aleyrodidarum_BT-QVLC_
Ca._Portiera_aleyrodidarum_BT-HRs
Ca._Carsonella_ruddii_DC
Ca._Carsonella_ruddii_HC
Ca._Carsonella_ruddii_PV
Ca._Evansia_muelleri
Halomonas_elongata_DSM-2581
Chromohalobacter_salexigens_DSM-3043
Kangiella_koreensis_DSM_16069
Marinomonas_posidonica_IVIA-Po-18
Marinomonas_mediterranea_MMB_1
Pseudomonas_aeruginosa_B136-33
68
87
100
100
94
100
100
96
100
100
0.1

CarB

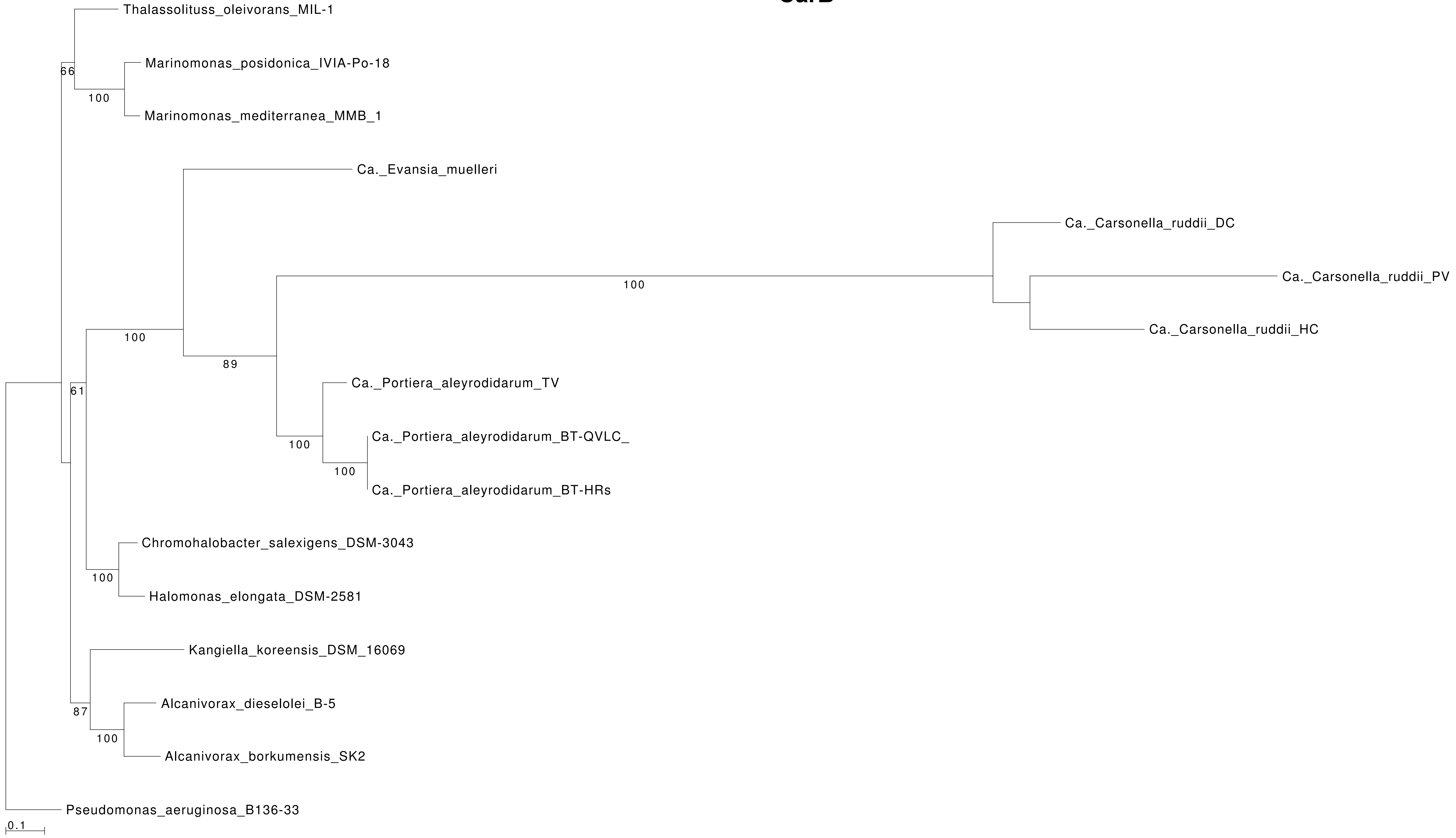
Thalassolituss_oleivorans_MIL-1
Marinomonas_posidonica_IVIA-Po-18
Marinomonas_mediterranea_MMB_1
Ca._Evansia_muelleri
Ca._Carsonella_ruddii_DC
Ca._Carsonella_ruddii_PV
Ca._Carsonella_ruddii_HC
Ca._Portiera_aleyrodidarum_TV
Ca._Portiera_aleyrodidarum_BT-QVLC_
Ca._Portiera_aleyrodidarum_BT-HRs
Chromohalobacter_salexigens_DSM-3043
Halomonas_elongata_DSM-2581
Kangiella_koreensis_DSM_16069
Alcanivorax_dieselolei_B-5
Alcanivorax_borkumensis_SK2
Pseudomonas_aeruginosa_B136-33
66
100
100
100
89
61
100
100
100
87
100
0.1

DnaB

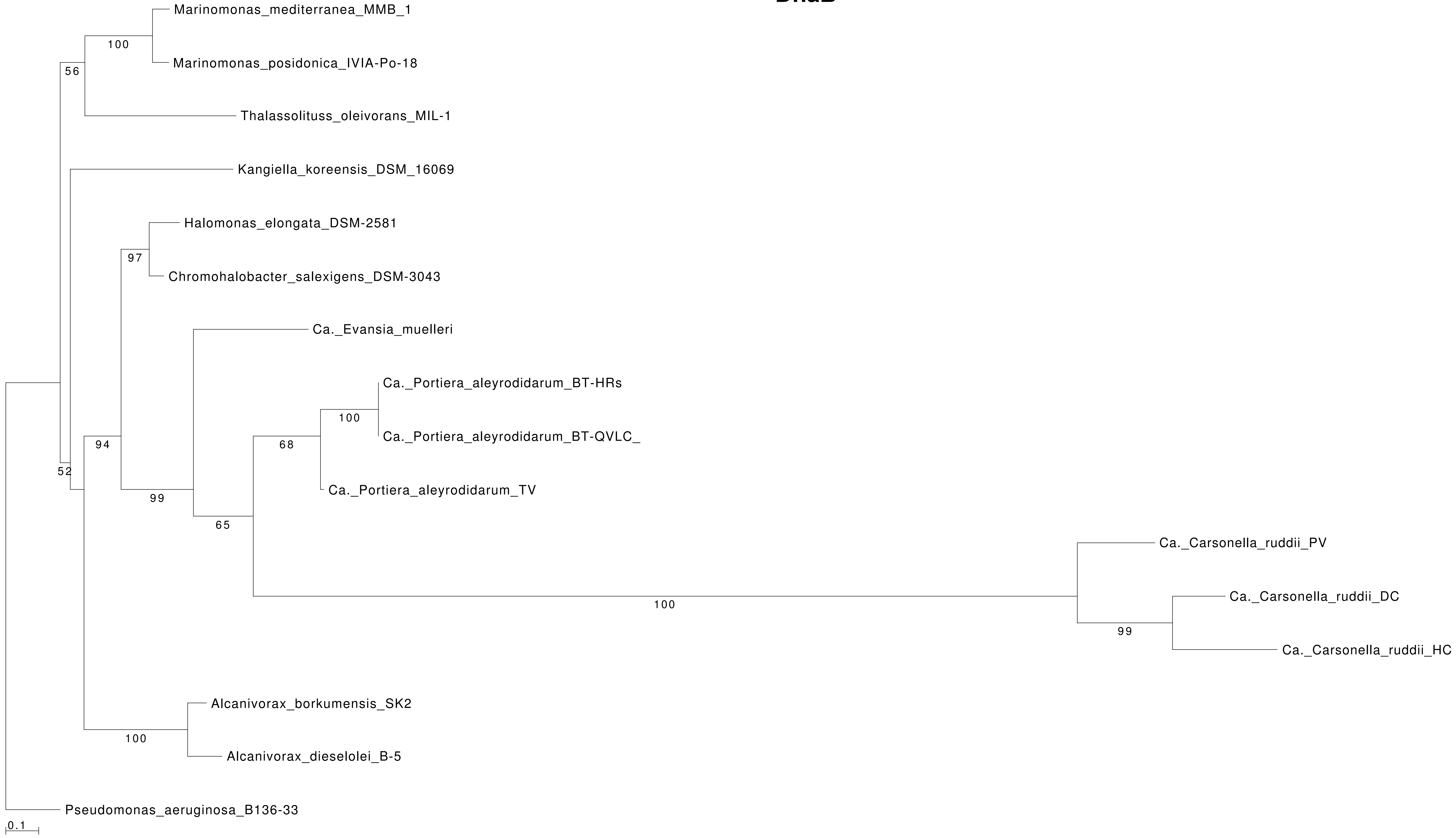
Marinomonas_mediterranea_MMB_1
100
Marinomonas_posidonica_IVIA-Po-18
56
Thalassolituss_oleivorans_MIL-1
Kangiella_koreensis_DSM_16069
Halomonas_elongata_DSM-2581
97
Chromohalobacter_salexigens_DSM-3043
Ca._Evansia_muelleri
Ca._Portiera_aleyrodidarum_BT-HRs
100
Ca._Portiera_aleyrodidarum_BT-QVLC_
94
68
52
Ca._Portiera_aleyrodidarum_TV
99
65
Ca._Carsonella_ruddii_PV
Ca._Carsonella_ruddii_DC
100
99
Ca._Carsonella_ruddii_HC
Alcanivorax_borkumensis_SK2
100
Alcanivorax_dieselolei_B-5
Pseudomonas_aeruginosa_B136-33
0.1

DnaE

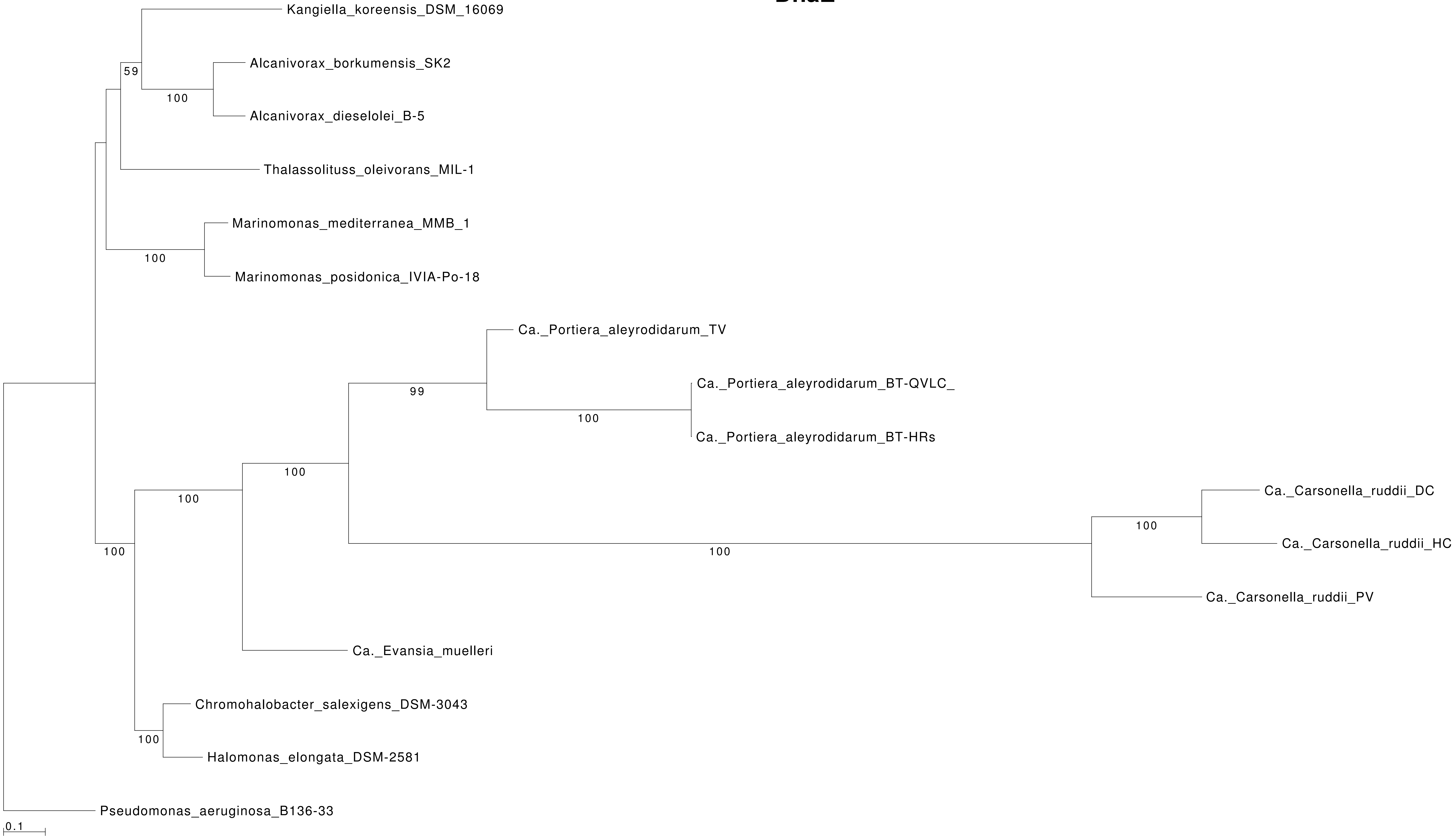

Kangiella_koreensis_DSM_16069
59
Alcanivorax_borkumensis_SK2
100
Alcanivorax_dieselolei_B-5
Thalassolituss_oleivorans_MIL-1
Marinomonas_mediterranea_MMB_1
100
Marinomonas_posidonica_IVIA-Po-18
Ca._Portiera_aleyrodidarum_TV
99
Ca._Portiera_aleyrodidarum_BT-QVLC_
100
Ca._Portiera_aleyrodidarum_BT-HRs
100
Ca._Carsonella_ruddii_DC
100
100
Ca._Carsonella_ruddii_HC
100
100
Ca._Carsonella_ruddii_PV
Ca._Evansia_muelleri
Chromohalobacter_salexigens_DSM-3043
100
Halomonas_elongata_DSM-2581
Pseudomonas_aeruginosa_B136-33
0.1

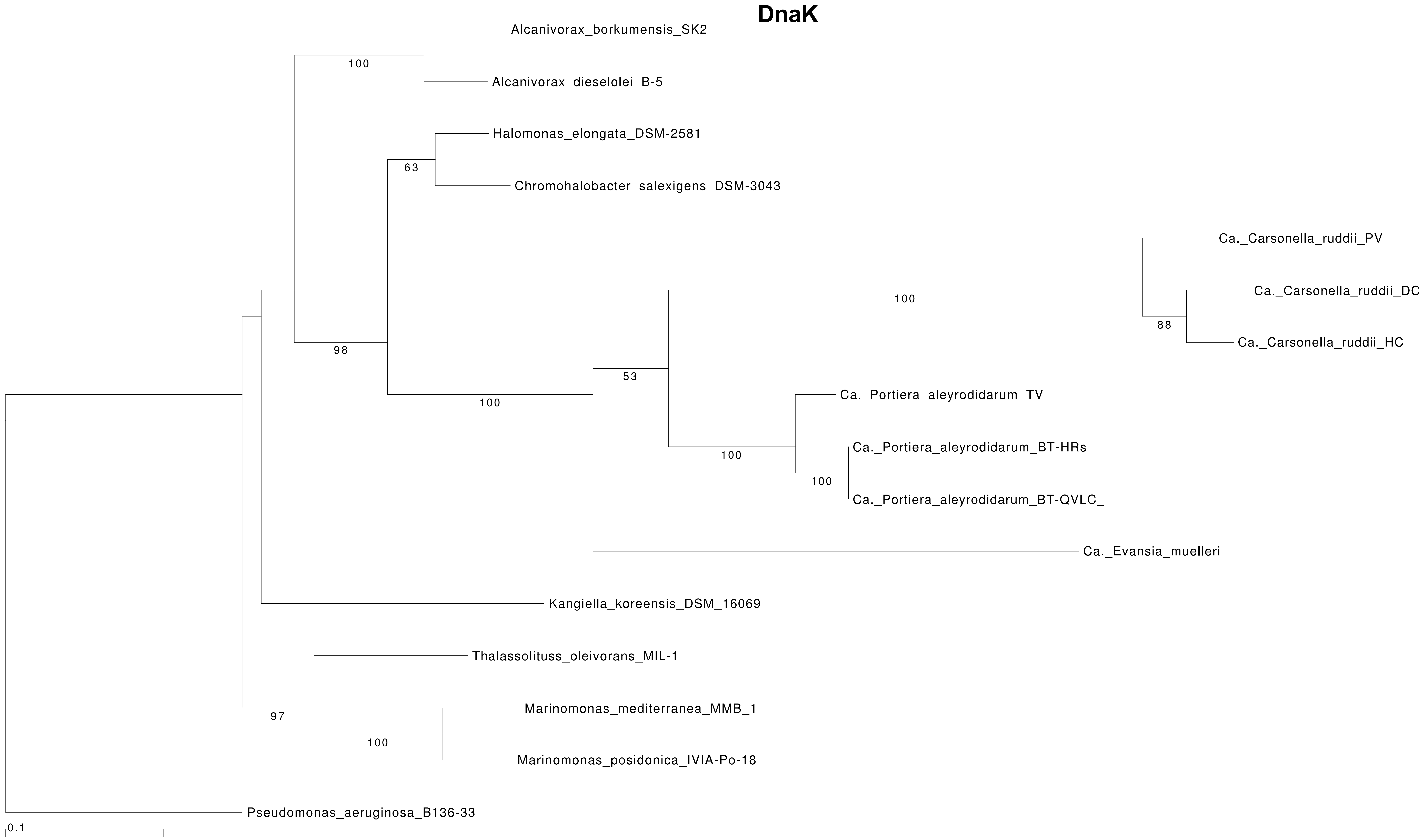

DnaK
Alcanivorax_borkumensis_SK2
100
Alcanivorax_dieselolei_B-5
Halomonas_elongata_DSM-2581
63
Chromohalobacter_salexigens_DSM-3043
Ca._Carsonella_ruddii_PV
100
Ca._Carsonella_ruddii_DC
88
Ca._Carsonella_ruddii_HC
98
53
Ca._Portiera_aleyrodidarum_TV
100
Ca._Portiera_aleyrodidarum_BT-HRs
100
100
Ca._Portiera_aleyrodidarum_BT-QVLC_
Ca._Evansia_muelleri
Kangiella_koreensis_DSM_16069
Thalassolituss_oleivorans_MIL-1
97
Marinomonas_mediterranea_MMB_1
100
Marinomonas_posidonica_IVIA-Po-18
Pseudomonas_aeruginosa_B136-33
0.1

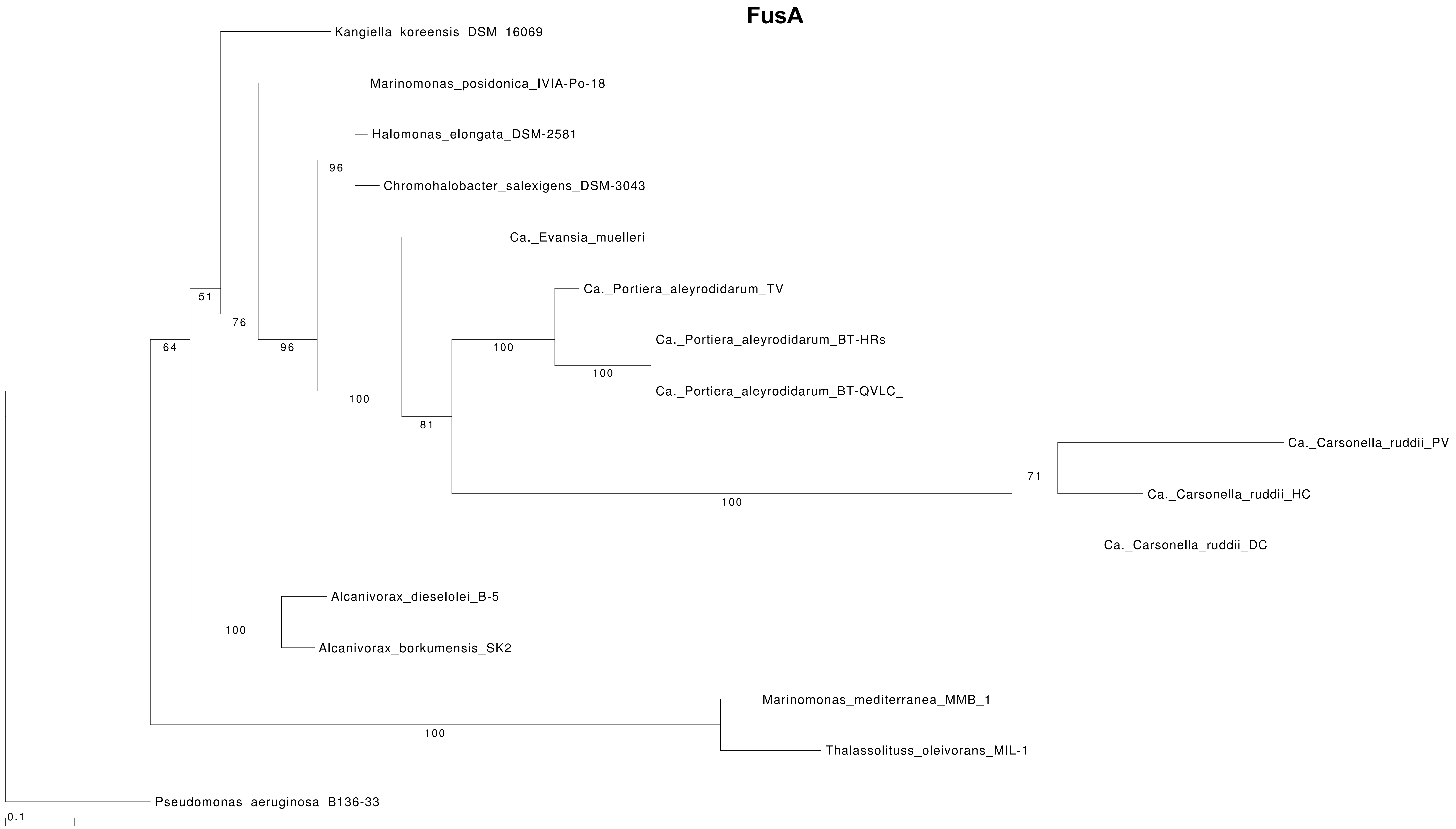
FusA
Kangiella_koreensis_DSM_16069
Marinomonas_posidonica_IVIA-Po-18
Halomonas_elongata_DSM-2581
Chromohalobacter_salexigens_DSM-3043
Ca._Evansia_muelleri
Ca._Portiera_aleyrodidarum_TV
Ca._Portiera_aleyrodidarum_BT-HRs
Ca._Portiera_aleyrodidarum_BT-QVLC_
Ca._Carsonella_ruddii_PV
Ca._Carsonella_ruddii_HC
Ca._Carsonella_ruddii_DC
Alcanivorax_dieselolei_B-5
Alcanivorax_borkumensis_SK2
Marinomonas_mediterranea_MMB_1
Thalassolituss_oleivorans_MIL-1
Pseudomonas_aeruginosa_B136-33
96
51
76
96
64
100
100
100
81
71
100
100
100
0.1

GroEL

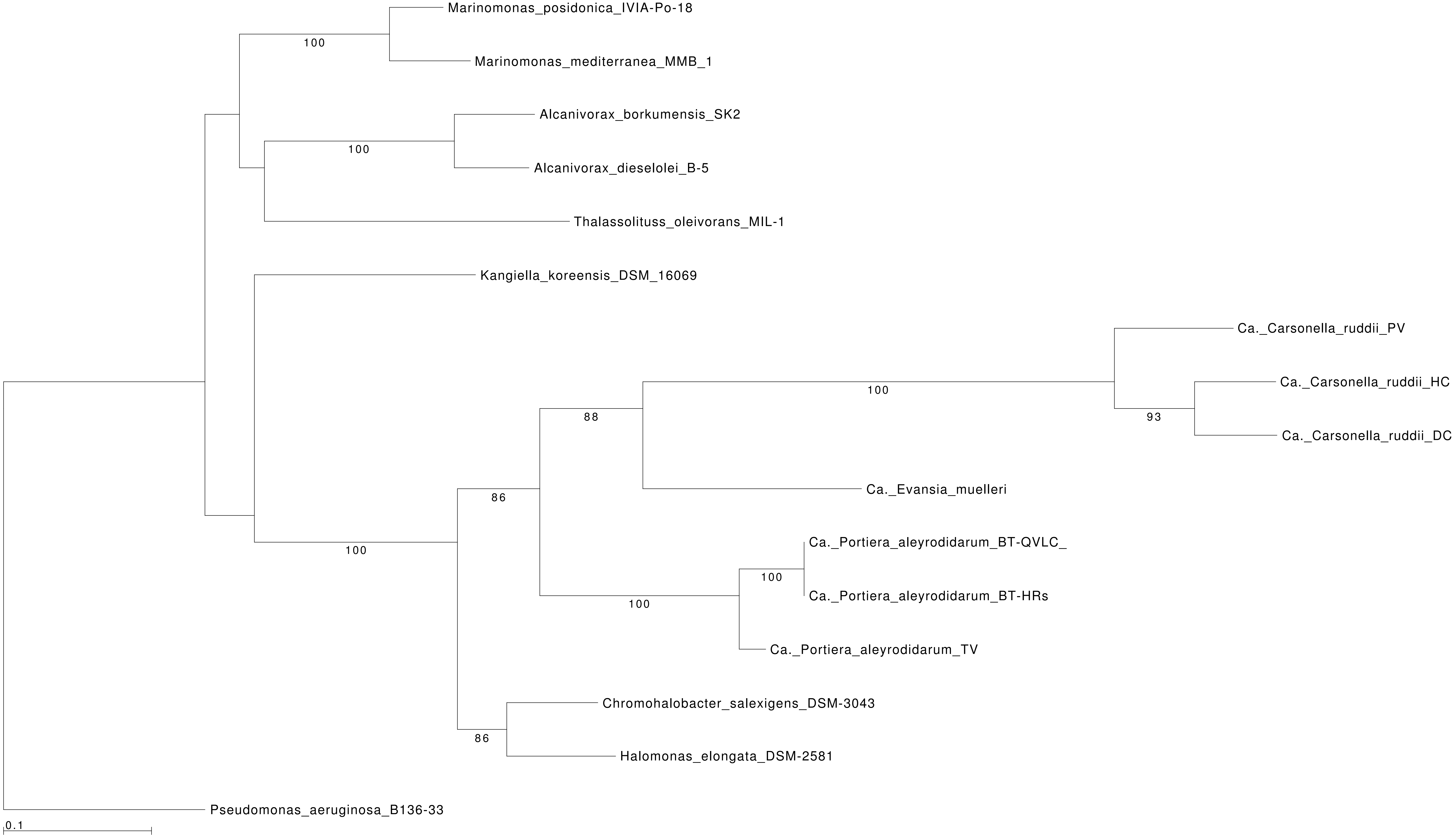

Marinomonas_posidonica_IVIA-Po-18
100
Marinomonas_mediterranea_MMB_1
Alcanivorax_borkumensis_SK2
100
Alcanivorax_dieselolei_B-5
Thalassolituss_oleivorans_MIL-1
Kangiella_koreensis_DSM_16069
Ca._Carsonella_ruddii_PV
100
Ca._Carsonella_ruddii_HC
88
93
Ca._Carsonella_ruddii_DC
Ca._Evansia_muelleri
86
Ca._Portiera_aleyrodidarum_BT-QVLC_
100
100
Ca._Portiera_aleyrodidarum_BT-HRs
100
Ca._Portiera_aleyrodidarum_TV
Chromohalobacter_salexigens_DSM-3043
86
Halomonas_elongata_DSM-2581
Pseudomonas_aeruginosa_B136-33
0.1

IleS

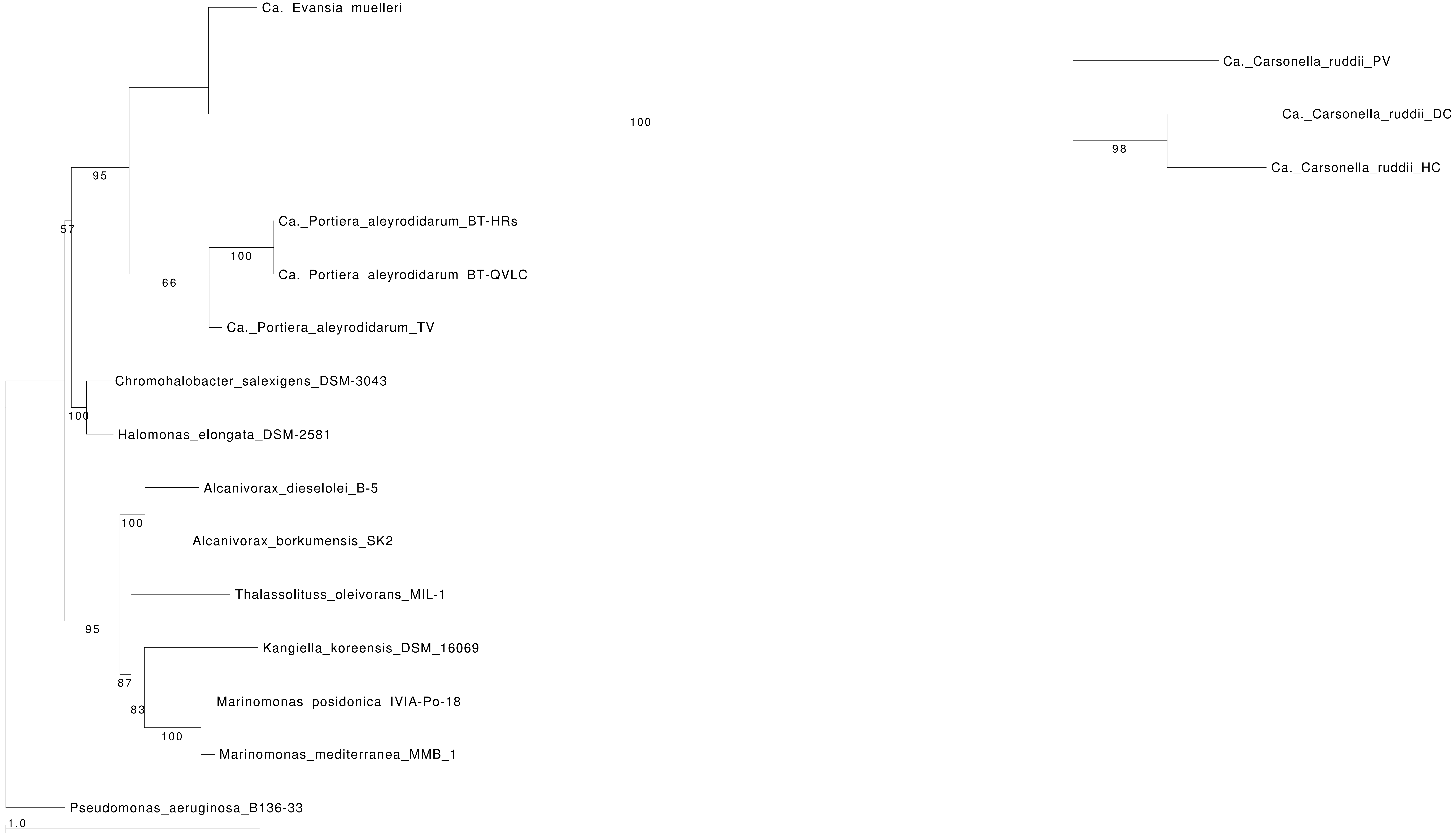

Ca._Evansia_muelleri
Ca._Carsonella_ruddii_PV
Ca._Carsonella_ruddii_DC
Ca._Carsonella_ruddii_HC
100
98
95
57
Ca._Portiera_aleyrodidarum_BT-HRs
Ca._Portiera_aleyrodidarum_BT-QVLC_
100
66
Ca._Portiera_aleyrodidarum_TV
Chromohalobacter_salexigens_DSM-3043
100
Halomonas_elongata_DSM-2581
Alcanivorax_dieselolei_B-5
100
Alcanivorax_borkumensis_SK2
Thalassolituss_oleivorans_MIL-1
95
Kangiella_koreensis_DSM_16069
87
83
Marinomonas_posidonica_IVIA-Po-18
100
Marinomonas_mediterranea_MMB_1
Pseudomonas_aeruginosa_B136-33
1.0

LeuB

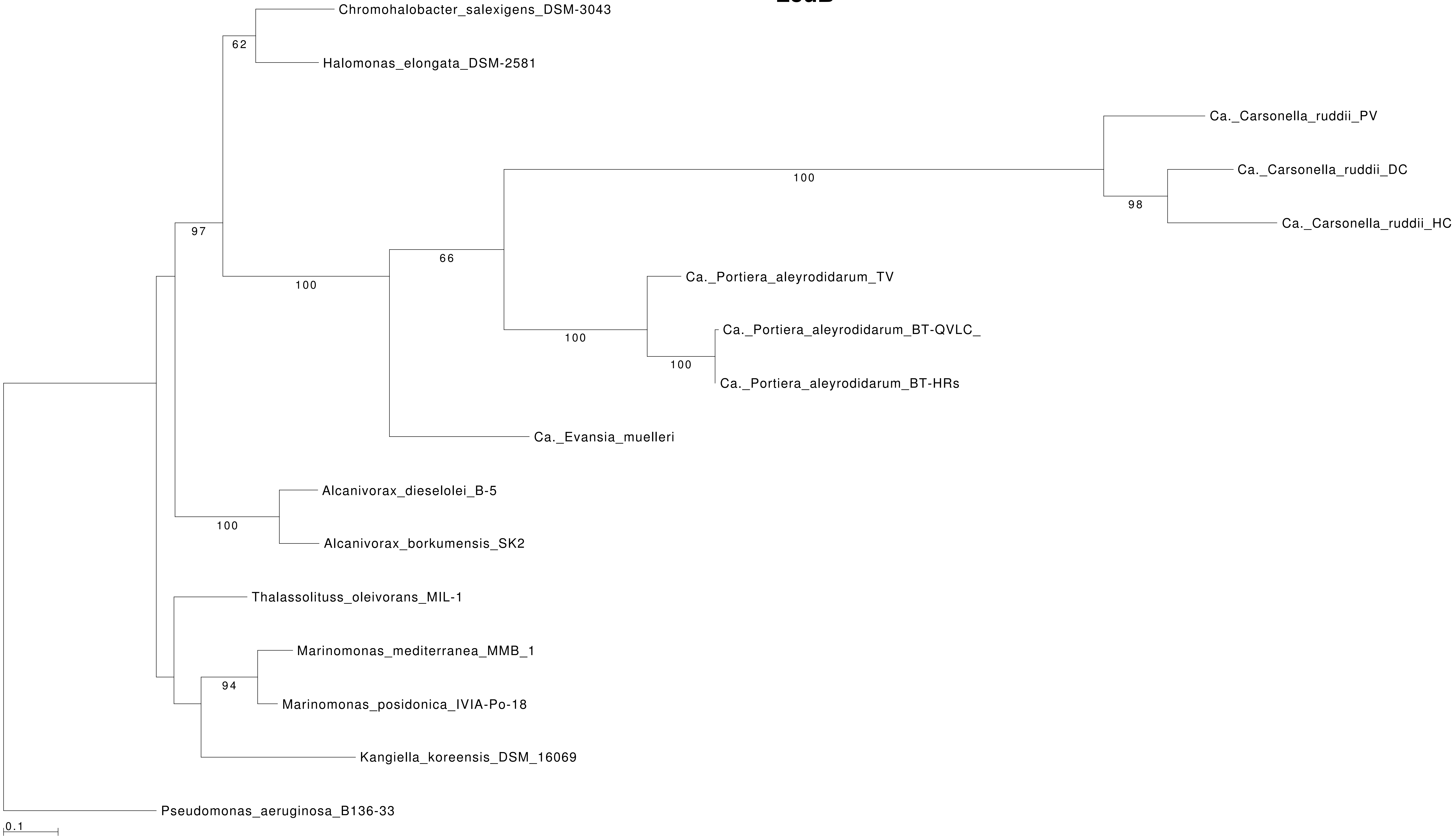

Chromohalobacter_salexigens_DSM-3043
Halomonas_elongata_DSM-2581
Ca._Carsonella_ruddii_PV
Ca._Carsonella_ruddii_DC
Ca._Carsonella_ruddii_HC
Ca._Portiera_aleyrodidarum_TV
Ca._Portiera_aleyrodidarum_BT-QVLC_
Ca._Portiera_aleyrodidarum_BT-HRs
Ca._Evansia_muelleri
Alcanivorax_dieselolei_B-5
Alcanivorax_borkumensis_SK2
Thalassolituss_oleivorans_MIL-1
Marinomonas_mediterranea_MMB_1
Marinomonas_posidonica_IVIA-Po-18
Kangiella_koreensis_DSM_16069
Pseudomonas_aeruginosa_B136-33
62
97
100
66
100
98
100
100
100
94
0.1

LeuC

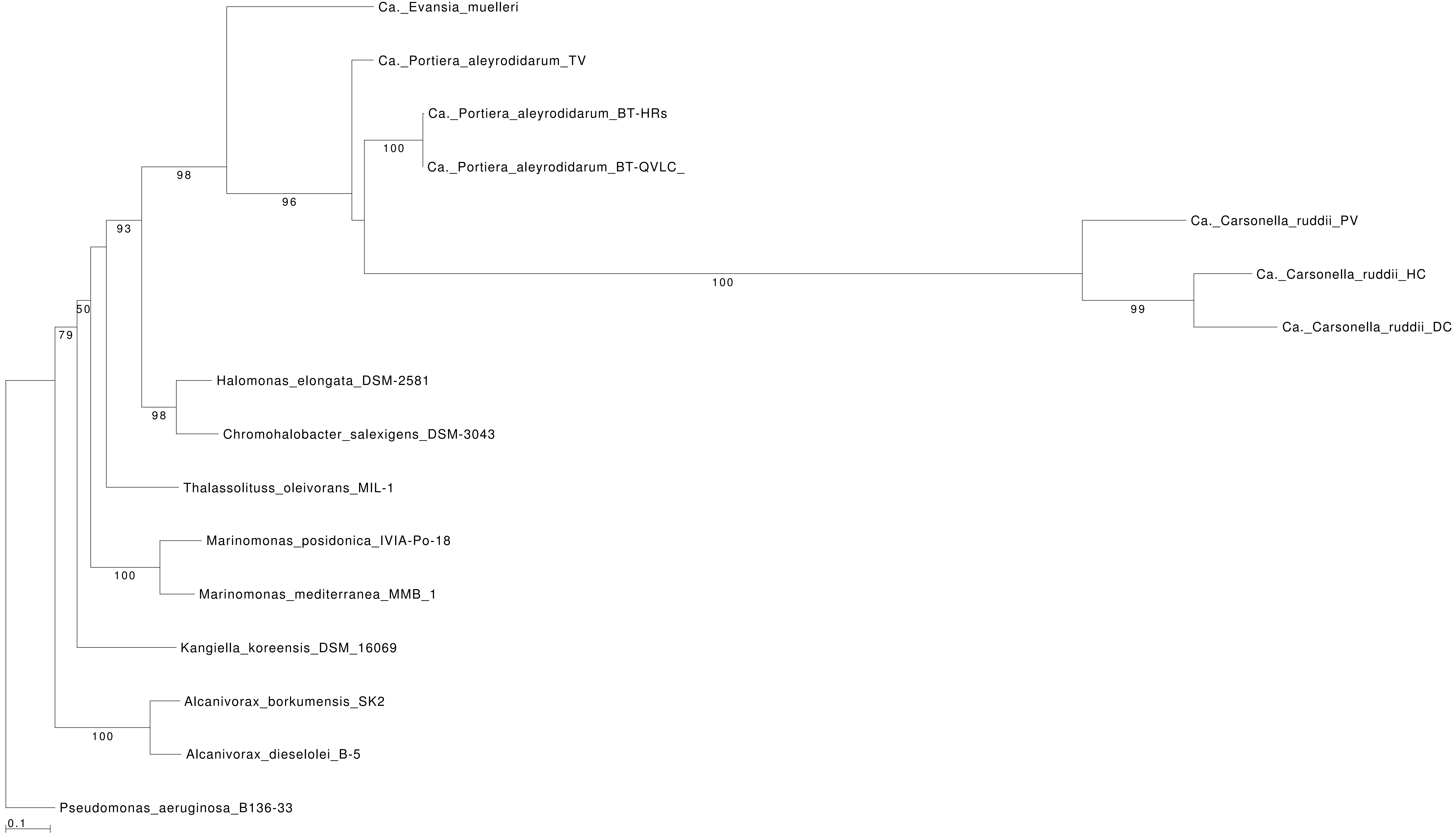

Ca._Evansia_muelleri
Ca._Portiera_aleyrodidarum_TV
Ca._Portiera_aleyrodidarum_BT-HRs
Ca._Portiera_aleyrodidarum_BT-QVLC_
Ca._Carsonella_ruddii_PV
Ca._Carsonella_ruddii_HC
Ca._Carsonella_ruddii_DC
Halomonas_elongata_DSM-2581
Chromohalobacter_salexigens_DSM-3043
Thalassolituss_oleivorans_MIL-1
Marinomonas_posidonica_IVIA-Po-18
Marinomonas_mediterranea_MMB_1
Kangiella_koreensis_DSM_16069
Alcanivorax_borkumensis_SK2
Alcanivorax_dieselolei_B-5
Pseudomonas_aeruginosa_B136-33
100
98
96
93
100
99
50
79
98
100
100
0.1

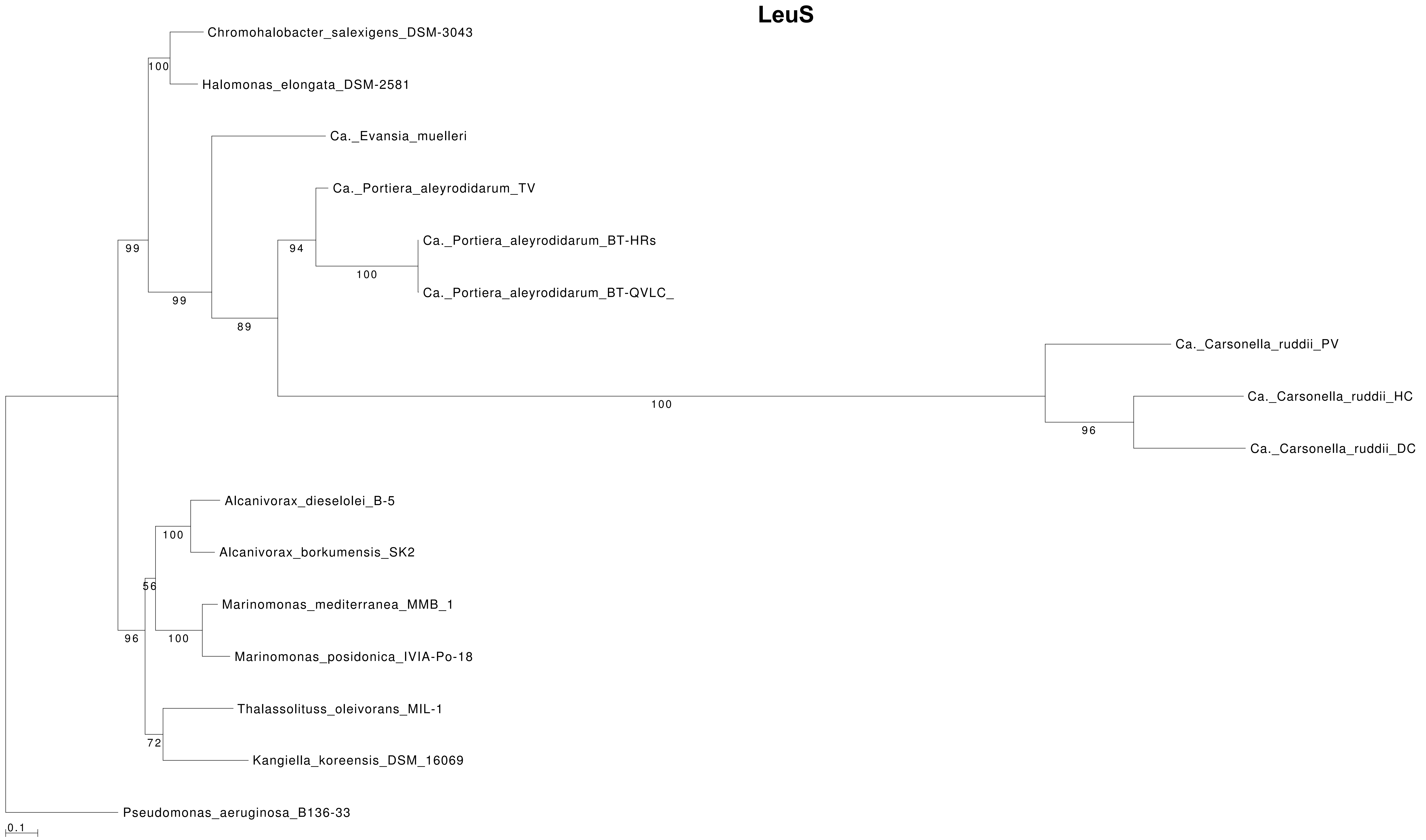
LeuS
Chromohalobacter_salexigens_DSM-3043
100
Halomonas_elongata_DSM-2581
Ca._Evansia_muelleri
Ca._Portiera_aleyrodidarum_TV
99
94
Ca._Portiera_aleyrodidarum_BT-HRs
100
Ca._Portiera_aleyrodidarum_BT-QVLC_
99
89
Ca._Carsonella_ruddii_PV
Ca._Carsonella_ruddii_HC
100
96
Ca._Carsonella_ruddii_DC
Alcanivorax_dieselolei_B-5
100
Alcanivorax_borkumensis_SK2
56
Marinomonas_mediterranea_MMB_1
96
100
Marinomonas_posidonica_IVIA-Po-18
Thalassolituss_oleivorans_MIL-1
72
Kangiella_koreensis_DSM_16069
Pseudomonas_aeruginosa_B136-33
0.1

LysC

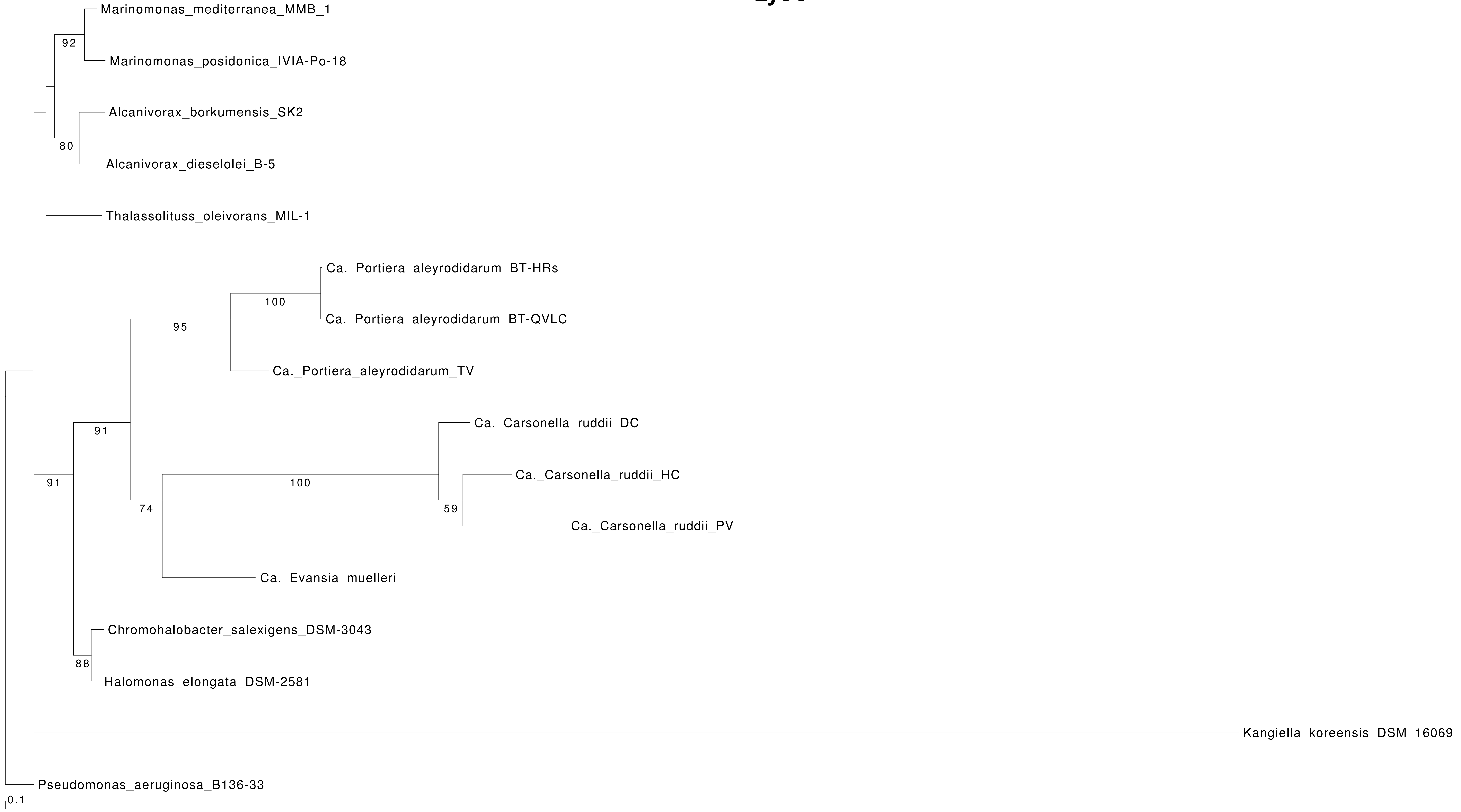

Marinomonas_mediterranea_MMB_1
92
Marinomonas_posidonica_IVIA-Po-18
Alcanivorax_borkumensis_SK2
80
Alcanivorax_dieselolei_B-5
Thalassolituss_oleivorans_MIL-1
Ca._Portiera_aleyrodidarum_BT-HRs
100
Ca._Portiera_aleyrodidarum_BT-QVLC_
95
Ca._Portiera_aleyrodidarum_TV
Ca._Carsonella_ruddii_DC
91
Ca._Carsonella_ruddii_HC
91
100
74
59
Ca._Carsonella_ruddii_PV
Ca._Evansia_muelleri
Chromohalobacter_salexigens_DSM-3043
88
Halomonas_elongata_DSM-2581
Kangiella_koreensis_DSM_16069
Pseudomonas_aeruginosa_B136-33
0.1

MnmE

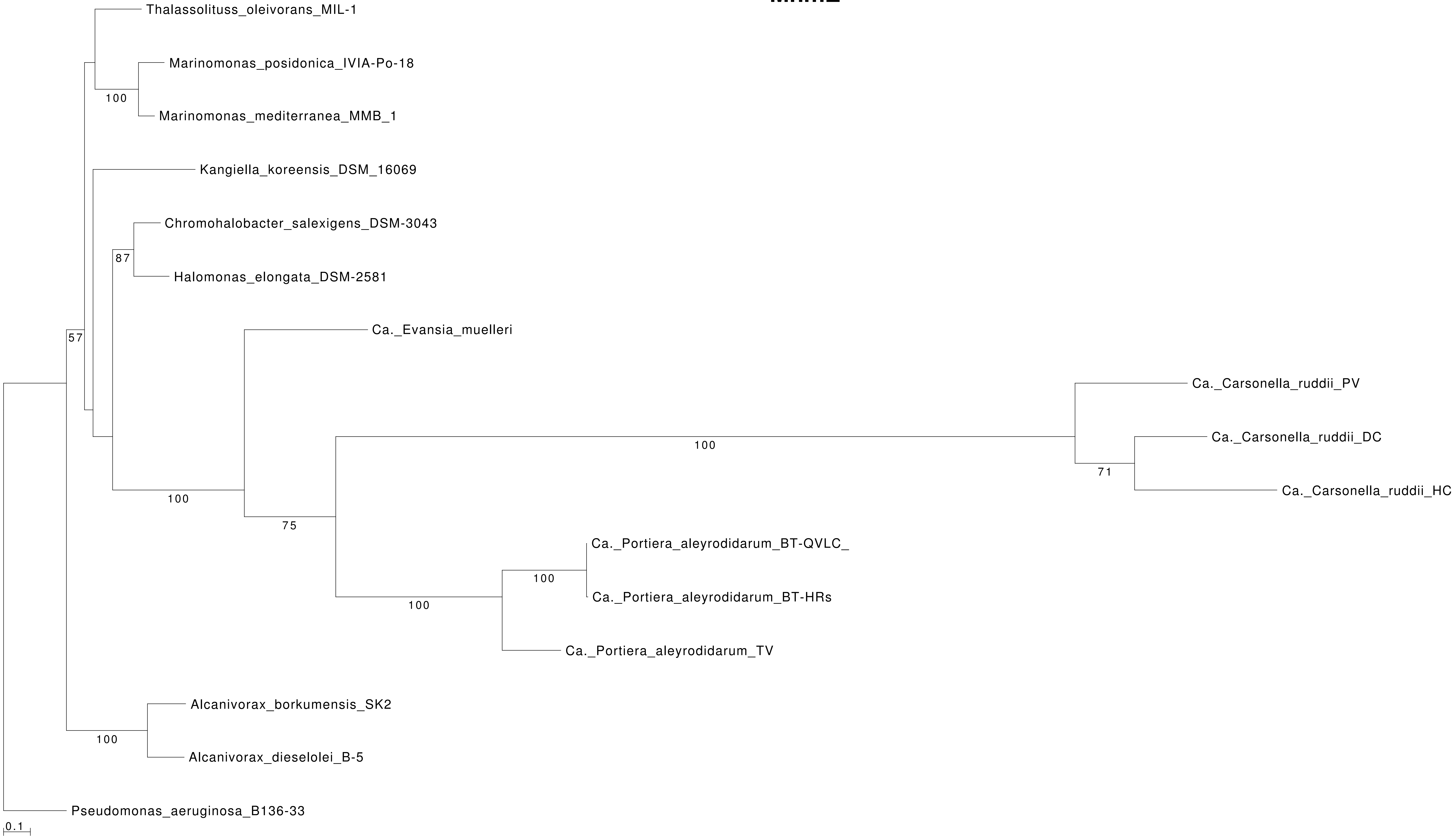
Thalassolituss_oleivorans_MIL-1
Marinomonas_posidonica_IVIA-Po-18
100
Marinomonas_mediterranea_MMB_1
Kangiella_koreensis_DSM_16069
Chromohalobacter_salexigens_DSM-3043
87
Halomonas_elongata_DSM-2581
57
Ca._Evansia_muelleri
Ca._Carsonella_ruddii_PV
100
Ca._Carsonella_ruddii_DC
71
Ca._Carsonella_ruddii_HC
100
75
Ca._Portiera_aleyrodidarum_BT-QVLC_
100
Ca._Portiera_aleyrodidarum_BT-HRs
100
Ca._Portiera_aleyrodidarum_TV
Alcanivorax_borkumensis_SK2
100
Alcanivorax_dieselolei_B-5
Pseudomonas_aeruginosa_B136-33
0.1

MnmG

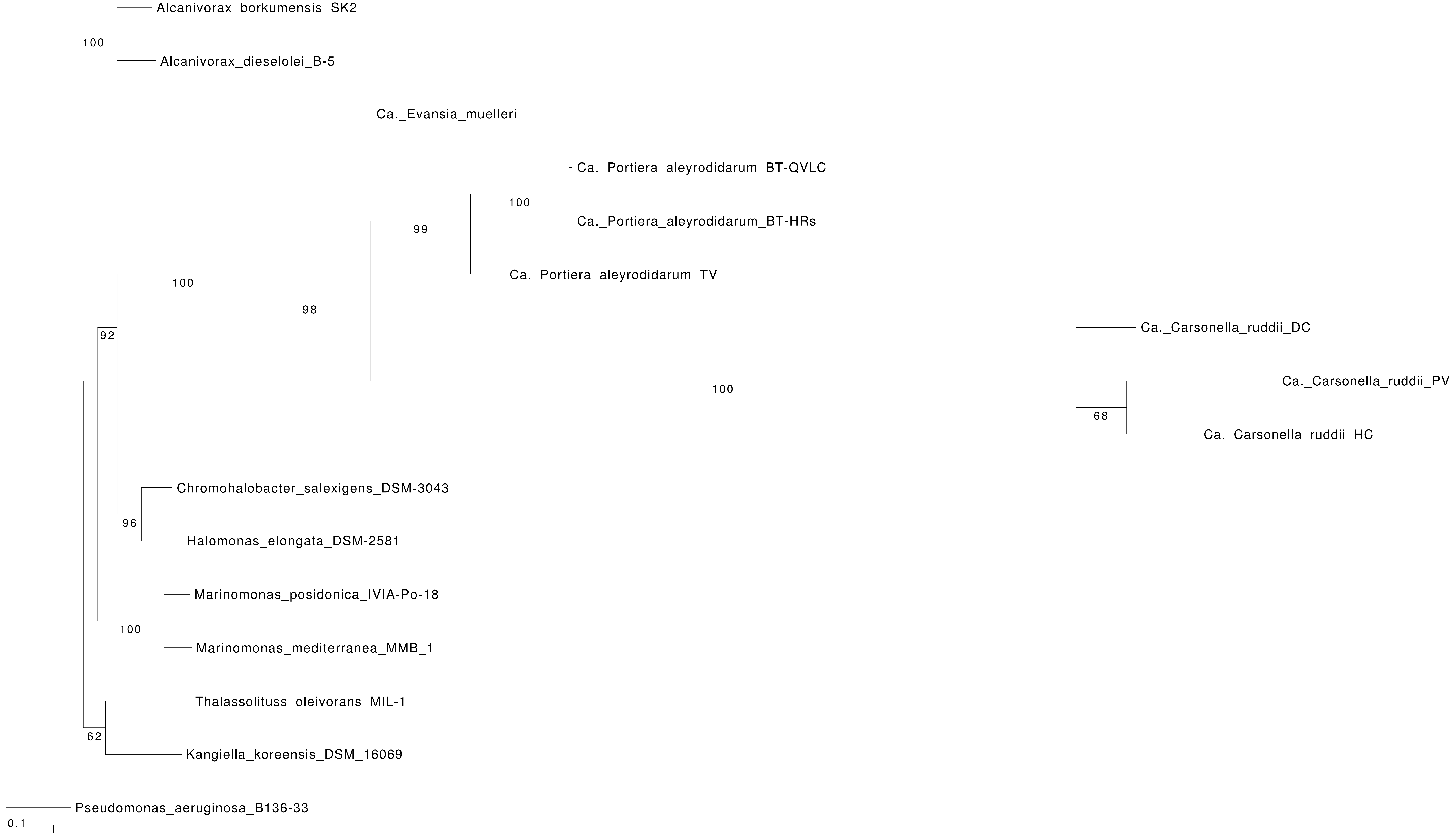

Alcanivorax_borkumensis_SK2
Alcanivorax_dieselolei_B-5
Ca._Evansia_muelleri
Ca._Portiera_aleyrodidarum_BT-QVLC_
Ca._Portiera_aleyrodidarum_BT-HRs
Ca._Portiera_aleyrodidarum_TV
Ca._Carsonella_ruddii_DC
Ca._Carsonella_ruddii_PV
Ca._Carsonella_ruddii_HC
Chromohalobacter_salexigens_DSM-3043
Halomonas_elongata_DSM-2581
Marinomonas_posidonica_IVIA-Po-18
Marinomonas_mediterranea_MMB_1
Thalassolituss_oleivorans_MIL-1
Kangiella_koreensis_DSM_16069
Pseudomonas_aeruginosa_B136-33
100
100
100
99
98
92
100
68
96
100
62
0.1

PrfA

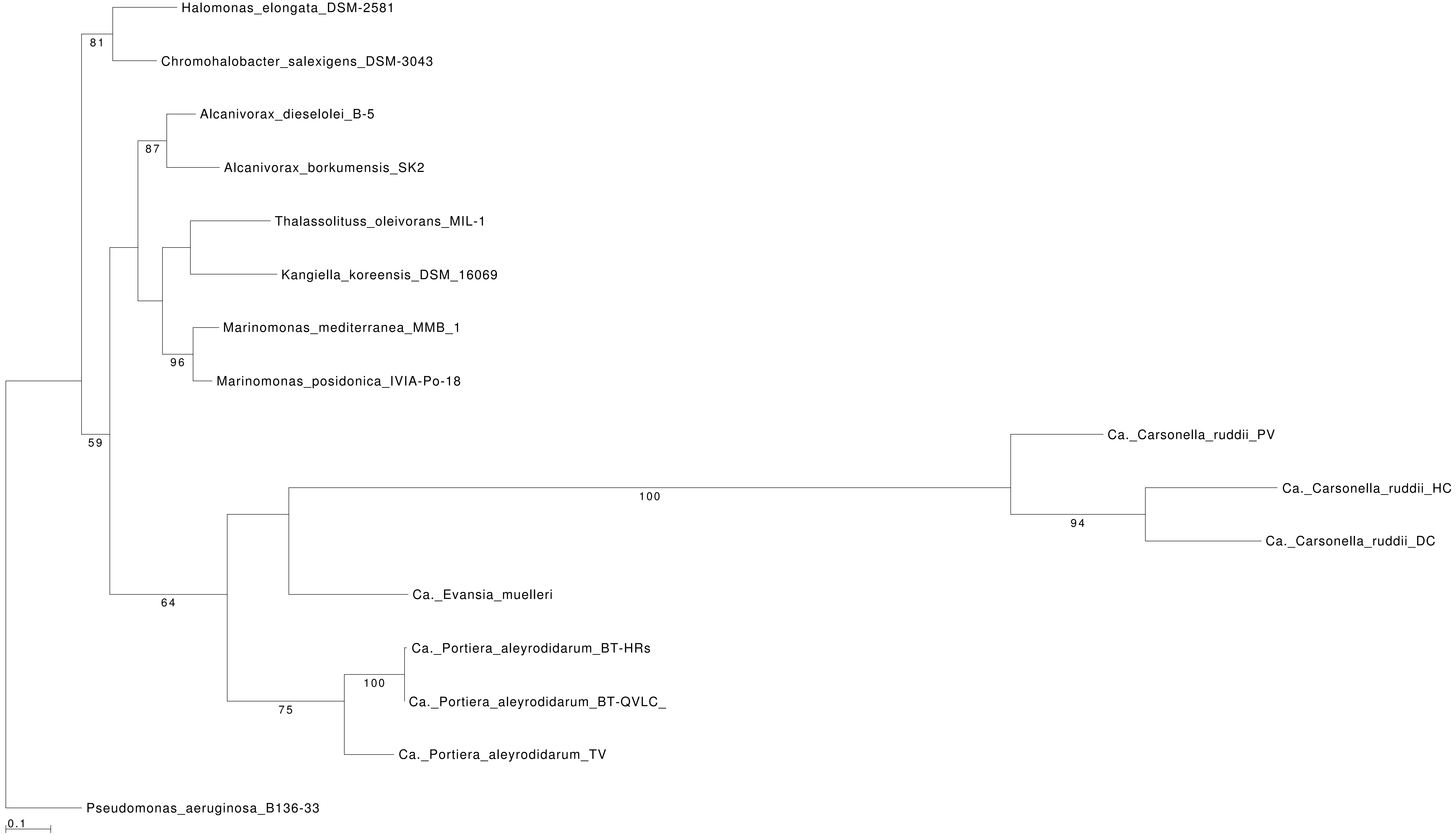
Halomonas_elongata_DSM-2581
Chromohalobacter_salexigens_DSM-3043
Alcanivorax_dieselolei_B-5
Alcanivorax_borkumensis_SK2
Thalassolituss_oleivorans_MIL-1
Kangiella_koreensis_DSM_16069
Marinomonas_mediterranea_MMB_1
Marinomonas_posidonica_IVIA-Po-18
Ca._Carsonella_ruddii_PV
Ca._Carsonella_ruddii_HC
Ca._Carsonella_ruddii_DC
Ca._Evansia_muelleri
Ca._Portiera_aleyrodidarum_BT-HRs
Ca._Portiera_aleyrodidarum_BT-QVLC_
Ca._Portiera_aleyrodidarum_TV
Pseudomonas_aeruginosa_B136-33
81
87
96
59
100
94
64
100
75
0.1

RplB

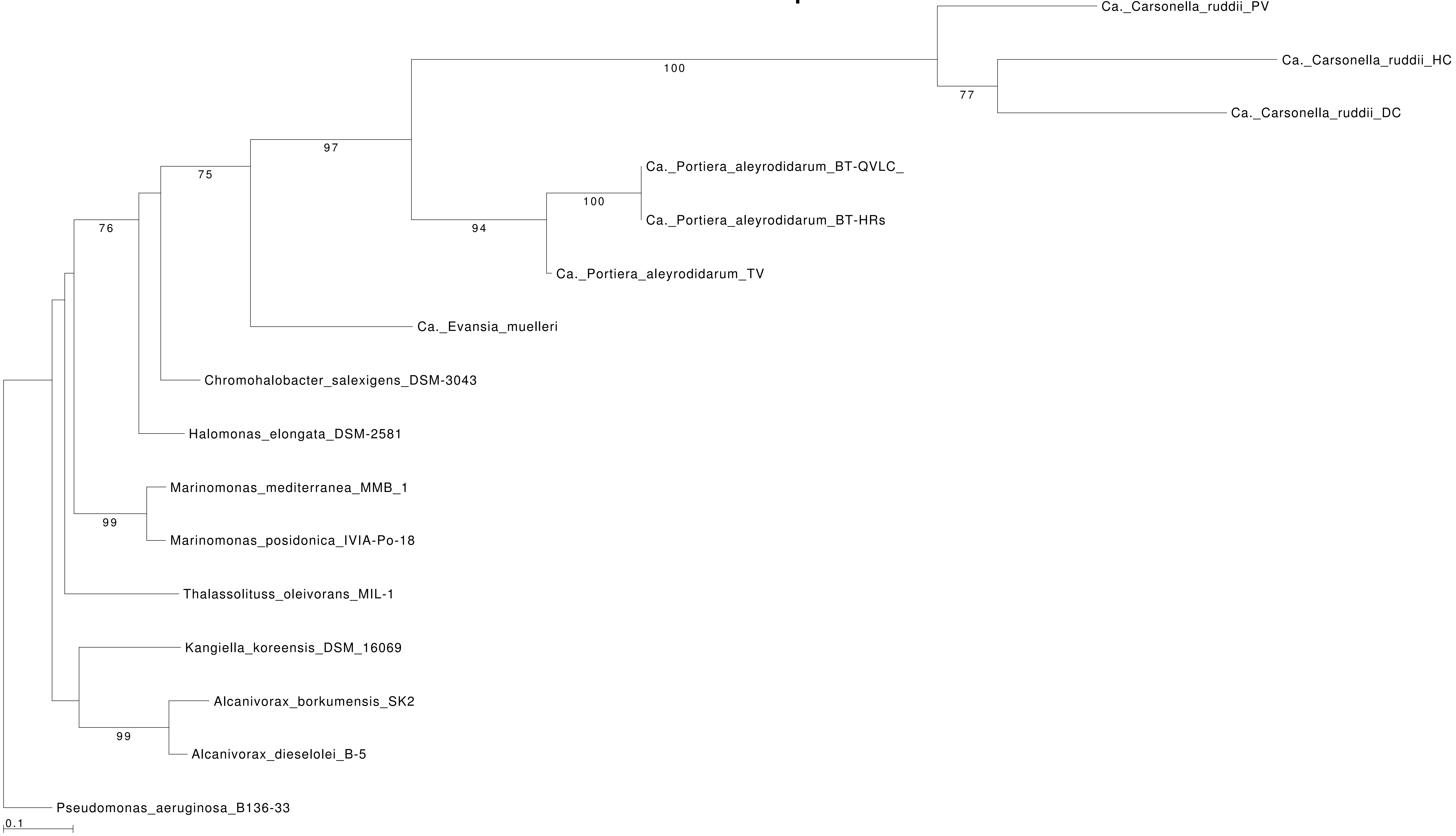

Ca._Carsonella_ruddii_PV
Ca._Carsonella_ruddii_HC
Ca._Carsonella_ruddii_DC
100
77
97
75
Ca._Portiera_aleyrodidarum_BT-QVLC_
100
Ca._Portiera_aleyrodidarum_BT-HRs
94
76
Ca._Portiera_aleyrodidarum_TV
Ca._Evansia_muelleri
Chromohalobacter_salexigens_DSM-3043
Halomonas_elongata_DSM-2581
Marinomonas_mediterranea_MMB_1
99
Marinomonas_posidonica_IVIA-Po-18
Thalassolituss_oleivorans_MIL-1
Kangiella_koreensis_DSM_16069
Alcanivorax_borkumensis_SK2
99
Alcanivorax_dieselolei_B-5
Pseudomonas_aeruginosa_B136-33
0.1

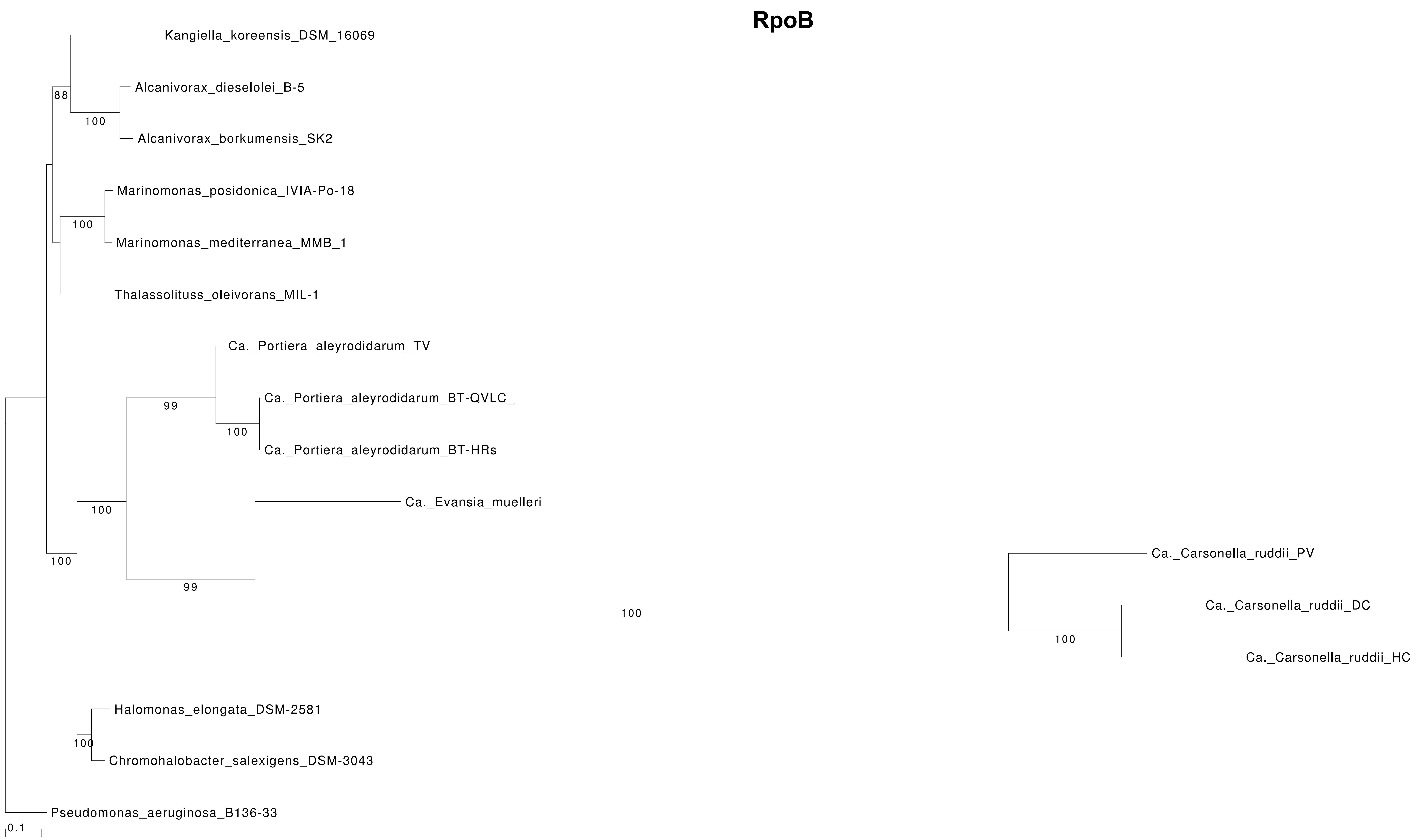
RpoB
Kangiella_koreensis_DSM_16069
Alcanivorax_dieselolei_B-5
Alcanivorax_borkumensis_SK2
Marinomonas_posidonica_IVIA-Po-18
Marinomonas_mediterranea_MMB_1
Thalassolituss_oleivorans_MIL-1
Ca._Portiera_aleyrodidarum_TV
Ca._Portiera_aleyrodidarum_BT-QVLC_
Ca._Portiera_aleyrodidarum_BT-HRs
Ca._Evansia_muelleri
Ca._Carsonella_ruddii_PV
Ca._Carsonella_ruddii_DC
Ca._Carsonella_ruddii_HC
Halomonas_elongata_DSM-2581
Chromohalobacter_salexigens_DSM-3043
Pseudomonas_aeruginosa_B136-33
88
100
100
99
100
100
99
100
100
100
100
0.1

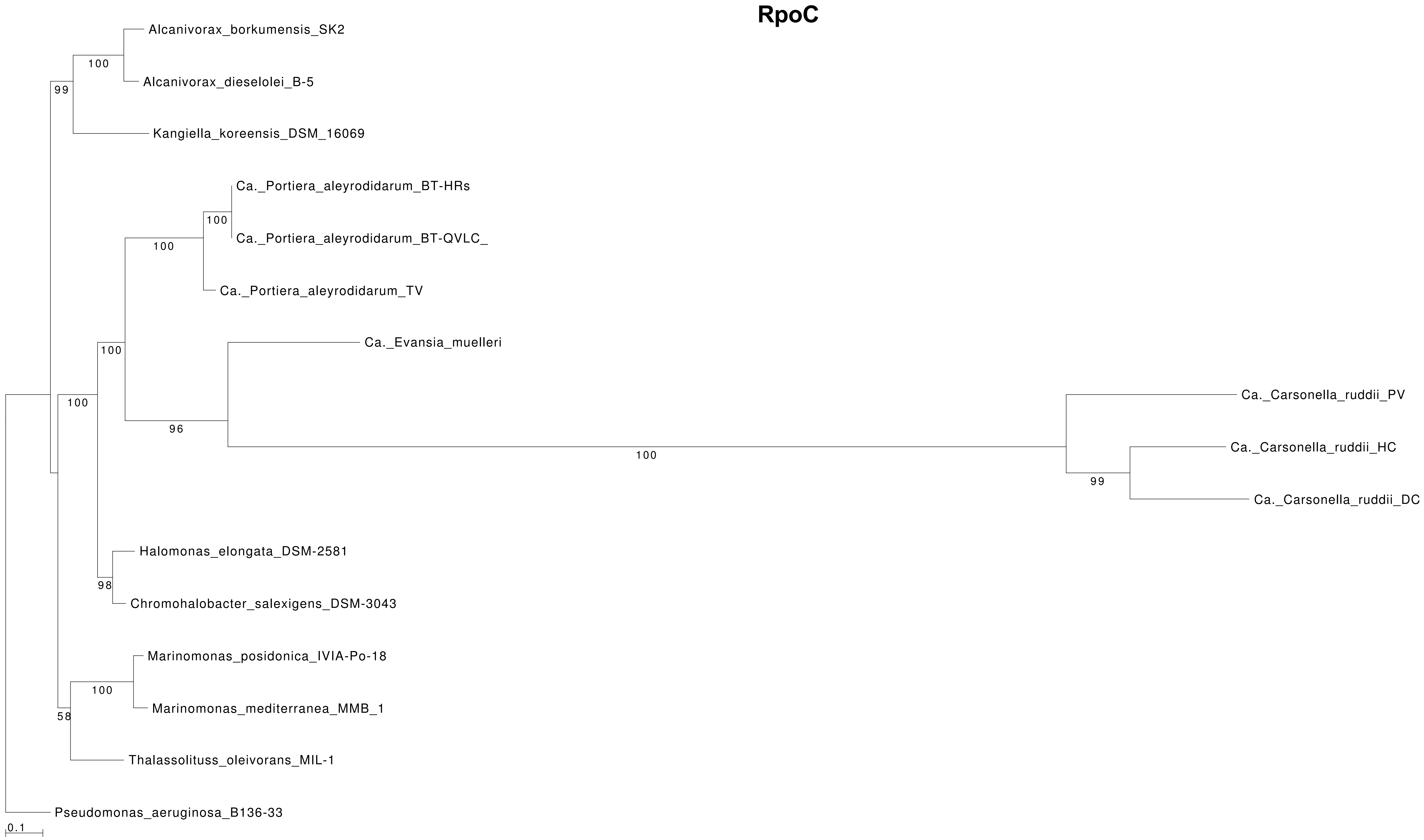
RpoC
Alcanivorax_borkumensis_SK2
Alcanivorax_dieselolei_B-5
Kangiella_koreensis_DSM_16069
Ca._Portiera_aleyrodidarum_BT-HRs
Ca._Portiera_aleyrodidarum_BT-QVLC_
Ca._Portiera_aleyrodidarum_TV
Ca._Evansia_muelleri
Ca._Carsonella_ruddii_PV
Ca._Carsonella_ruddii_HC
Ca._Carsonella_ruddii_DC
Halomonas_elongata_DSM-2581
Chromohalobacter_salexigens_DSM-3043
Marinomonas_posidonica_IVIA-Po-18
Marinomonas_mediterranea_MMB_1
Thalassolituss_oleivorans_MIL-1
Pseudomonas_aeruginosa_B136-33
100
99
100
100
100
100
96
100
99
98
100
58
0.1

Tu

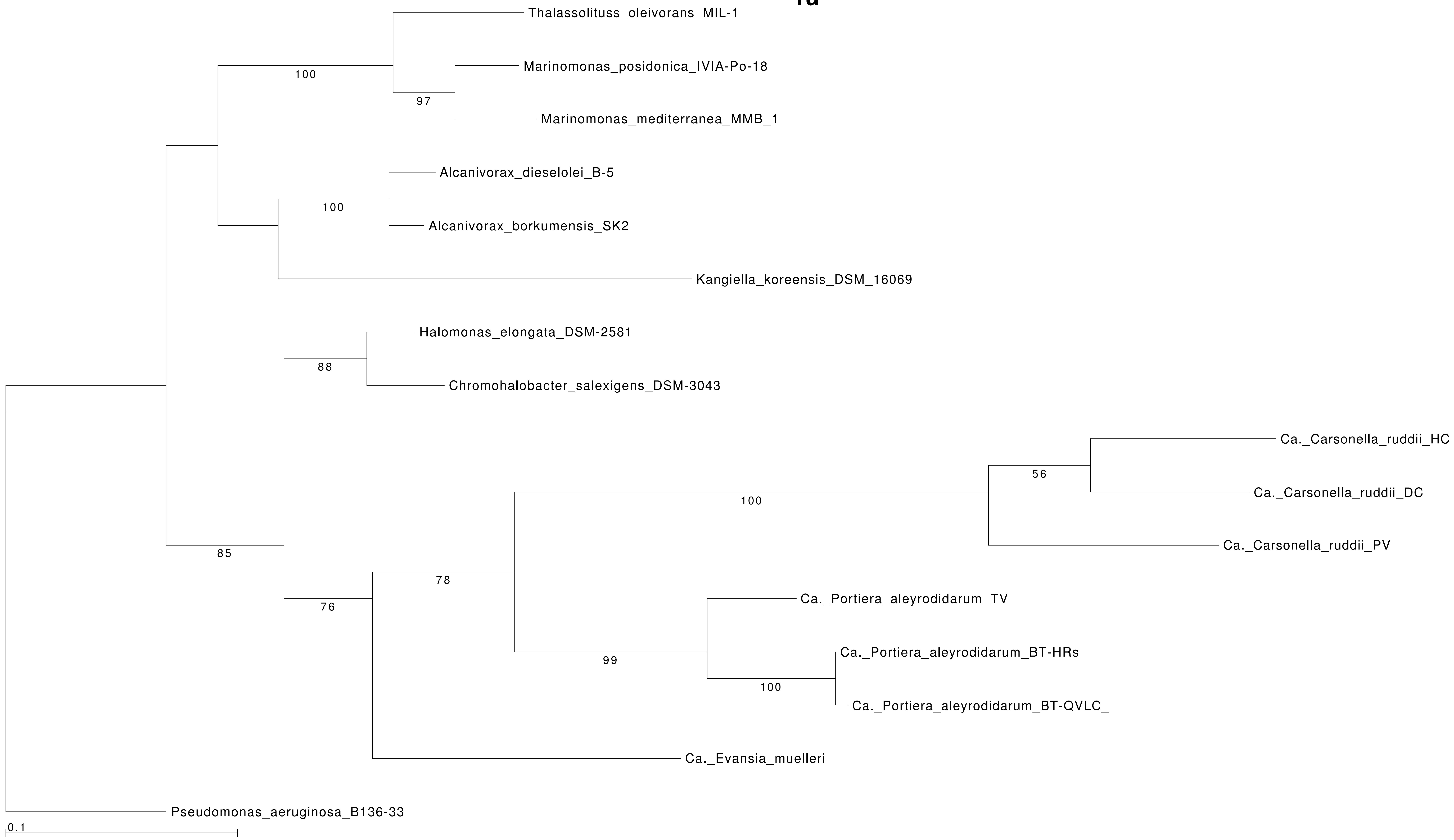
Thalassolituss_oleivorans_MIL-1
100
Marinomonas_posidonica_IVIA-Po-18
97
Marinomonas_mediterranea_MMB_1
Alcanivorax_dieselolei_B-5
100
Alcanivorax_borkumensis_SK2
Kangiella_koreensis_DSM_16069
Halomonas_elongata_DSM-2581
88
Chromohalobacter_salexigens_DSM-3043
Ca._Carsonella_ruddii_HC
56
Ca._Carsonella_ruddii_DC
100
Ca._Carsonella_ruddii_PV
85
78
Ca._Portiera_aleyrodidarum_TV
76
Ca._Portiera_aleyrodidarum_BT-HRs
99
100
Ca._Portiera_aleyrodidarum_BT-QVLC_
Ca._Evansia_muelleri
Pseudomonas_aeruginosa_B136-33
0.1

ValS

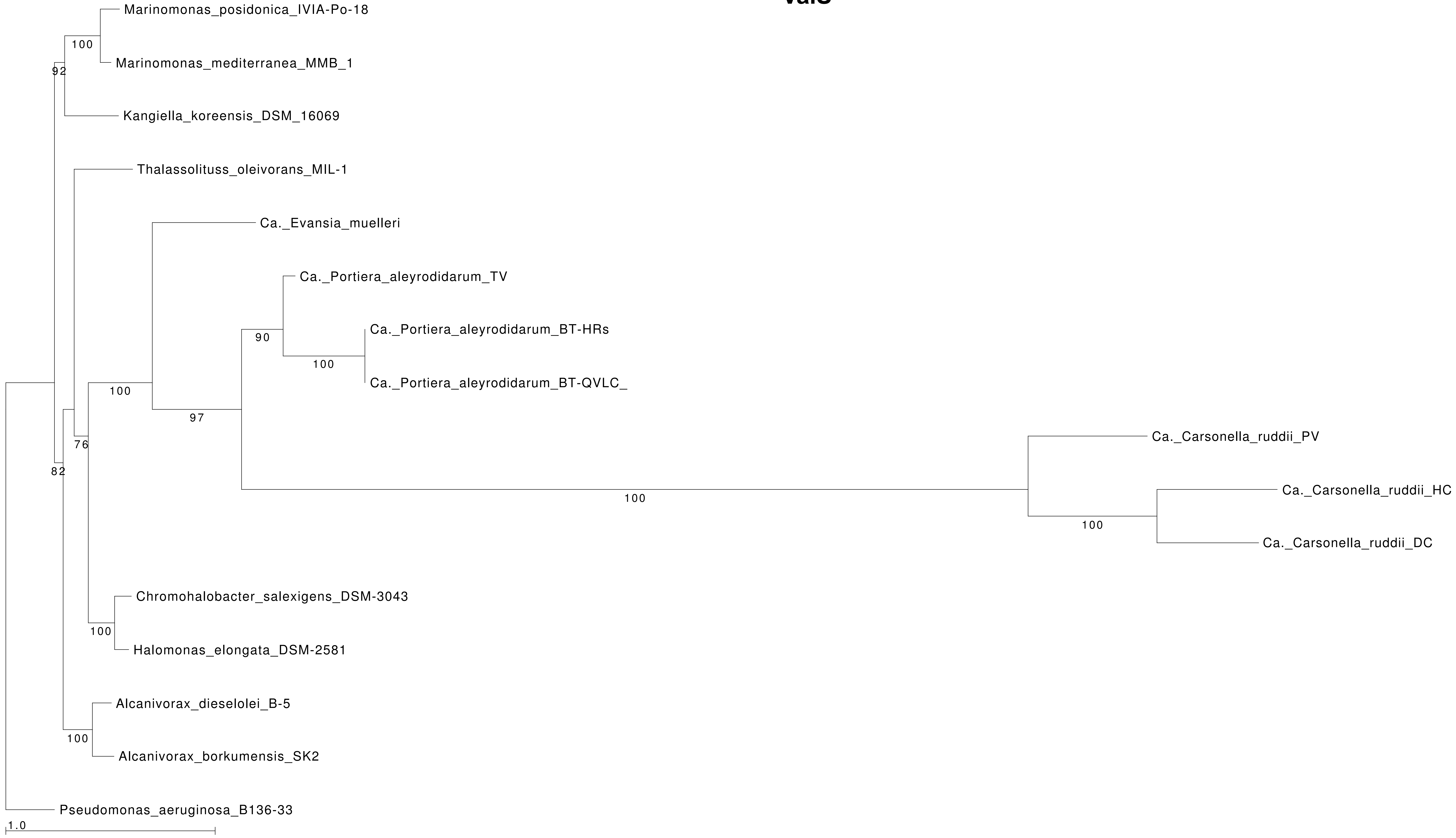
Marinomonas_posidonica_IVIA-Po-18
Marinomonas_mediterranea_MMB_1
Kangiella_koreensis_DSM_16069
Thalassolituss_oleivorans_MIL-1
Ca._Evansia_muelleri
Ca._Portiera_aleyrodidarum_TV
Ca._Portiera_aleyrodidarum_BT-HRs
Ca._Portiera_aleyrodidarum_BT-QVLC_
Ca._Carsonella_ruddii_PV
Ca._Carsonella_ruddii_HC
Ca._Carsonella_ruddii_DC
Chromohalobacter_salexigens_DSM-3043
Halomonas_elongata_DSM-2581
Alcanivorax_dieselolei_B-5
Alcanivorax_borkumensis_SK2
Pseudomonas_aeruginosa_B136-33
100
92
90
100
100
97
76
82
100
100
100
100
100
1.0
